# Direct air capture enables sustainable methanol production in water-scarce regions

**DOI:** 10.1038/s41467-026-76865-x

**Published:** 2026-08-24

**Authors:** Henrik Wenzel, Thomas Schöb, David S. Sholl, Jochen Linßen, Detlef Stolten, Jann M. Weinand

**Affiliations:** 1 Forschungszentrum Jülich GmbH, Institute of Climate and Energy Systems, Jülich Systems Analysis (ICE-2), 52425 Jülich, Germany; 2 RWTH Aachen University, Chair for Fuel Cells, Faculty of Mechanical Engineering, 52062 Aachen, Germany; 3 University of Tennessee - Oak Ridge Innovation Institute, Oak Ridge National Laboratory, Oak Ridge, TN 37830 USA; 4 Present Address: Department of Chemical & Biomolecular Engineering, Rice University, Houston, TX 77005 USA

**Keywords:** Carbon capture and storage, Energy modelling, Renewable energy, Energy and society, Water resources

## Abstract

Methanol is an essential chemical for the global economy, but its production typically requires substantial water inputs. Here we investigate the techno-economic potential of water-conscious methanol production across more than 20,000 regions worldwide by integrating direct air capture (DAC) with electrolysis, methanol synthesis and a renewable energy supply system. In 97% of these regions, water availability from ambient air is sufficient, and constraints in the remaining regions can be mitigated through adjusted system design and operation. Our results further highlight the importance of modeling DAC’s weather dependency, as system energy efficiency fluctuates between 39 to 49%, primarily due to variations in DAC energy demand. Air cooling – often overlooked in prior assessments – is identified as a viable strategy for water-conscious cooling, incurring only minor cost increases. Ultimately, the proposed system enables sustainable methanol production in arid regions worldwide by overcoming the water-energy nexus and sourcing all necessary feedstocks directly from ambient air.

## Abstract (web summary)

A global assessment shows that direct air capture can harvest both CO_2_ and water from ambient air across diverse climates, enabling renewable methanol production even in water-scarce regions.

## Introduction

Direct air capture (DAC) has gained significant attention in recent years, not only for its potential to provide negative carbon dioxide (CO_2_) emissions^1,2^, but also as a carbon source for renewable fuel production^3,4^. Within the scope of renewable DAC-based fuels, long chained hydrocarbons produced by Fischer-Tropsch synthesis, such as sustainable aviation fuels (SAFs), are often discussed^3,5^ alongside renewable methanol^6,7^. While large quantities of SAF will potentially be needed in the future^8^, this study focuses on DAC-based methanol (MeOH) production, given that methanol is one of the most essential chemicals in the global economy^9^. Current applications are spanning from formaldehyde and olefin production^10^ to its emerging role as a clean fuel for maritime transport^11^. Global demand has nearly doubled over the past decade, reaching an estimated 110 Mt a^−1^ by 2025^10^ with market prices ranging between 300 and 600 $ t_MeOH_^−1,^ ^12^. Additionally, projections suggest that demand could exceed 500 Mt a^−1^ by 2050^11^. However, conventional methanol production is heavily reliant on fossil feedstocks such as natural gas and coal, accounting for greenhouse gas emissions of ~0.3 Gt_CO2_ a^−1^, corresponding to 10% of the total chemical industry’s emissions^10^. Therefore, to meet global climate targets, transitioning towards renewable methanol production is imperative.

In addition, methanol production requires substantial water input – both as a feedstock and for cooling – yet many studies assume water to be freely available. This assumption is increasingly problematic, as water-stress intensifies worldwide^13^. Currently, around 40% of planned green hydrogen projects are located in water-stressed regions^14^, where water supply constraints could lead to operational limitations or even plant shutdowns, as frequently observed in the thermal power industry^15^. Nevertheless, these generally arid regions are often abundant in renewable energy resources, making them theoretically well-suited for energy-intensive production of methanol or other DAC-based fuels^16^, if water constraints can be addressed.

Despite numerous assessments of renewable methanol production pathways, most studies remain limited in scope, often neglecting factors such as feedstock provision and energy supply^17^. In our previous review we analyzed 62 green methanol production pathways from 46 publications regarding their system boundaries and utilized technologies^17^. While most assessments consider hydrogen supply, CO_2_ is often assumed to be available at a fixed cost. Out of the 18 pathways that consider CO_2_ supply, the majority models carbon capture from industrial processes. We find that only eight out of 62 analyzed pathways consider the complete process chain including feedstock provision and the necessary energy supply system^17^. However, water-conscious production is not in the scope of any of these assessments. While some more holistic evaluations have been performed recently^6^, water availability remains an overlooked factor. Additionally, water supply from DAC for other downstream processes has only been investigated in a few studies^18–20^. To the best of the authors’ knowledge, no comprehensive assessments for water-conscious production of methanol or other hydrocarbons exist to date.

**Fig. 1:**
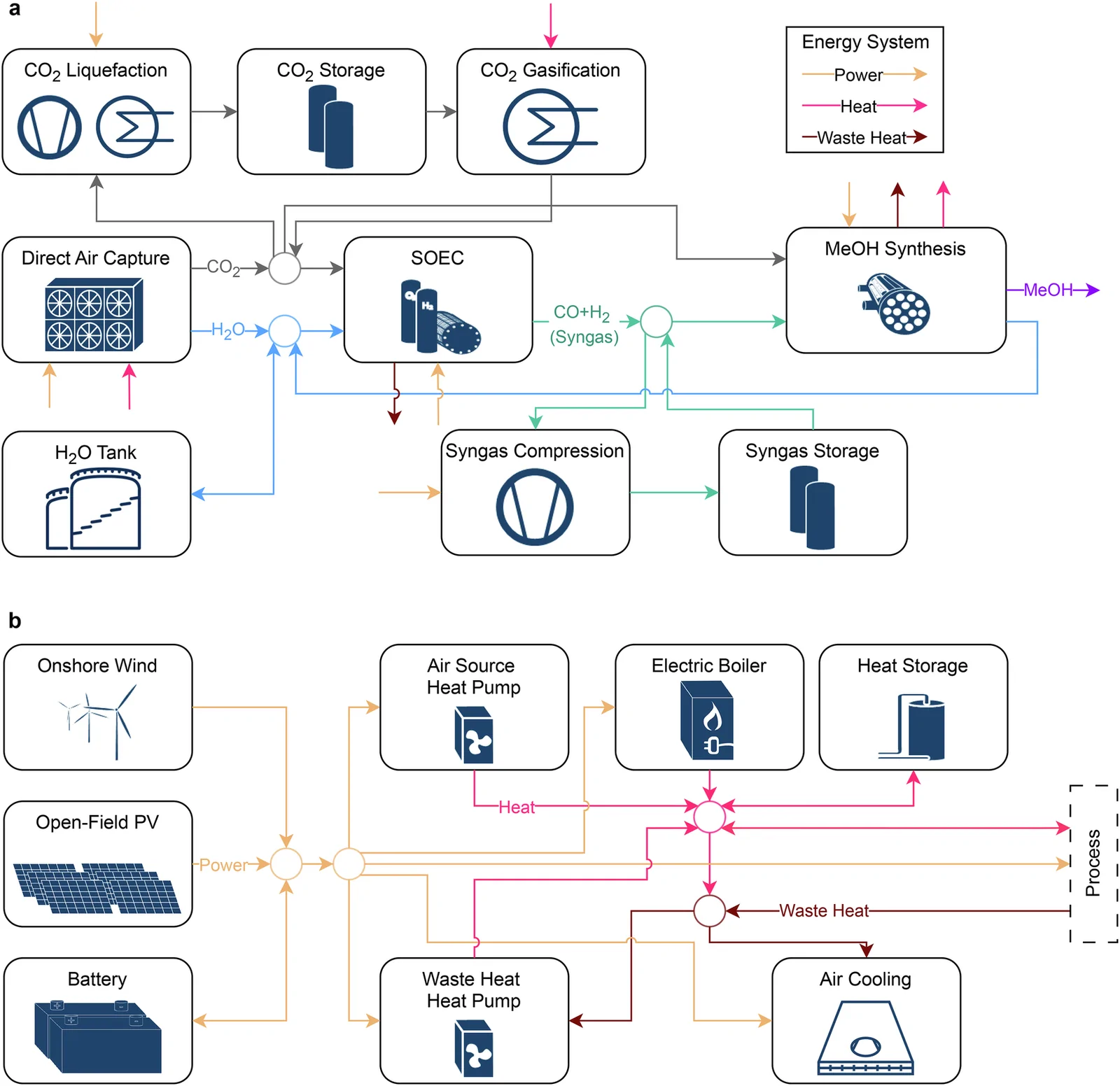
Overview of the process chain investigated and the connected energy system. All potential commodity flows are indicated by arrows. **a** Overview of the process chain including all necessary components for intermediate storage. Additionally, CO_2_ and H_2_O purge are allowed, if necessary. The heat required by the CO_2_ gasification unit could also be supplied by waste heat, as the temperature is low enough. **b** Overview of the connected renewable energy supply system. Heat can be supplied by heat pumps or an electric boiler. The waste heat can either be used in a heat pump or it has to be dissipated by an air cooling system. SOEC Solid Oxide Electrolysis Cell, PV Photovoltaic.

Therefore, in this study, a system for water-conscious green methanol production is assessed, which could enable renewable energy-rich but water-scarce regions to participate in the energy transition. The considered process chain (Fig. 1a) utilizes solid sorbent DAC for supply of both CO_2_ and water (H_2_O) directly from ambient air^16^. While water co-adsorption is typically seen as a burden as it substantially increases the energy needed for desorption, it could be used for water-conscious production of methanol or other hydrocarbons, supplying the necessary feedstocks without depleting freshwater resources. Thus, in the system investigated, all required water is either directly provided by the DAC plant or sourced from buffer storage during times of insufficient water supply from ambient air. The captured CO_2_-H_2_O mixture is subsequently processed via solid oxide electrolysis (SOEC), followed by methanol synthesis. To evaluate the feasibility of water-conscious green methanol production, a detailed techno-economic model is developed, which integrates the process chain and a renewable energy supply system (Fig. 1b). The energy supply system is modeled utilizing potentials of single-axis tracking open-field photovoltaic (OFPV) systems and onshore wind plants derived from prior land eligibility analysis in cooperation with the International Energy Agency (see Methods)^14,21^. Hourly resolved power generation profiles based on historic weather data are employed and the weather dependency of DAC is considered by combining a process model^22^ with hourly resolved temperature and relative humidity data for each region (see Methods). This way, the fluctuating energy demand, water co-adsorption and productivity of DAC systems under varying weather conditions are included. Finally, to avoid the significant water consumption of conventional cooling systems^23,24^, a weather-dependent air cooling system is modeled (see Methods) and employed to satisfy the cooling demand of the process chain.

For the coupled system, techno-economic optimization^25^ is performed to estimate the methanol production cost as well as the optimized system designs and operational strategies. In a first step, the maximum technical potential is derived for each region individually by utilizing all available renewable energy potential. Subsequently, a share of this potential is exogenously set as methanol demand, and the system is cost-optimized to derive the required capacities of all system components, as well as the methanol production cost (see Methods). The analysis has been conducted for more than 20,000 regions, corresponding to the second administrative level as defined in the global administrative boundaries dataset^26^, in 78 countries across the globe. The selected countries are projected to face significant water-stress by 2050, reflected by a water-stress index of at least medium-high according to the World Resources Insitute^13^, making water-conscious production essential.

By systematically analyzing production costs, water dependencies, and system performance across varying climatic conditions, this work provides a comprehensive assessment of the potential for large-scale, water-autarkic green methanol production. The detailed investigation of methanol serves as a boundary case and allows for general statements about the feasibility of water supply from DAC for hydrocarbon production, given that methanol has the highest hydrogen-to-carbon ratio. However, detailed studies for other DAC-based fuels, such as Fischer-Tropsch products or synthetic methane, need to be performed to investigate not only the technical potential but also the economic feasibility of the various possible process chains for sustainable hydrocarbon production.

## Results

### Levelized cost of water-conscious green methanol production

**Fig. 2:**
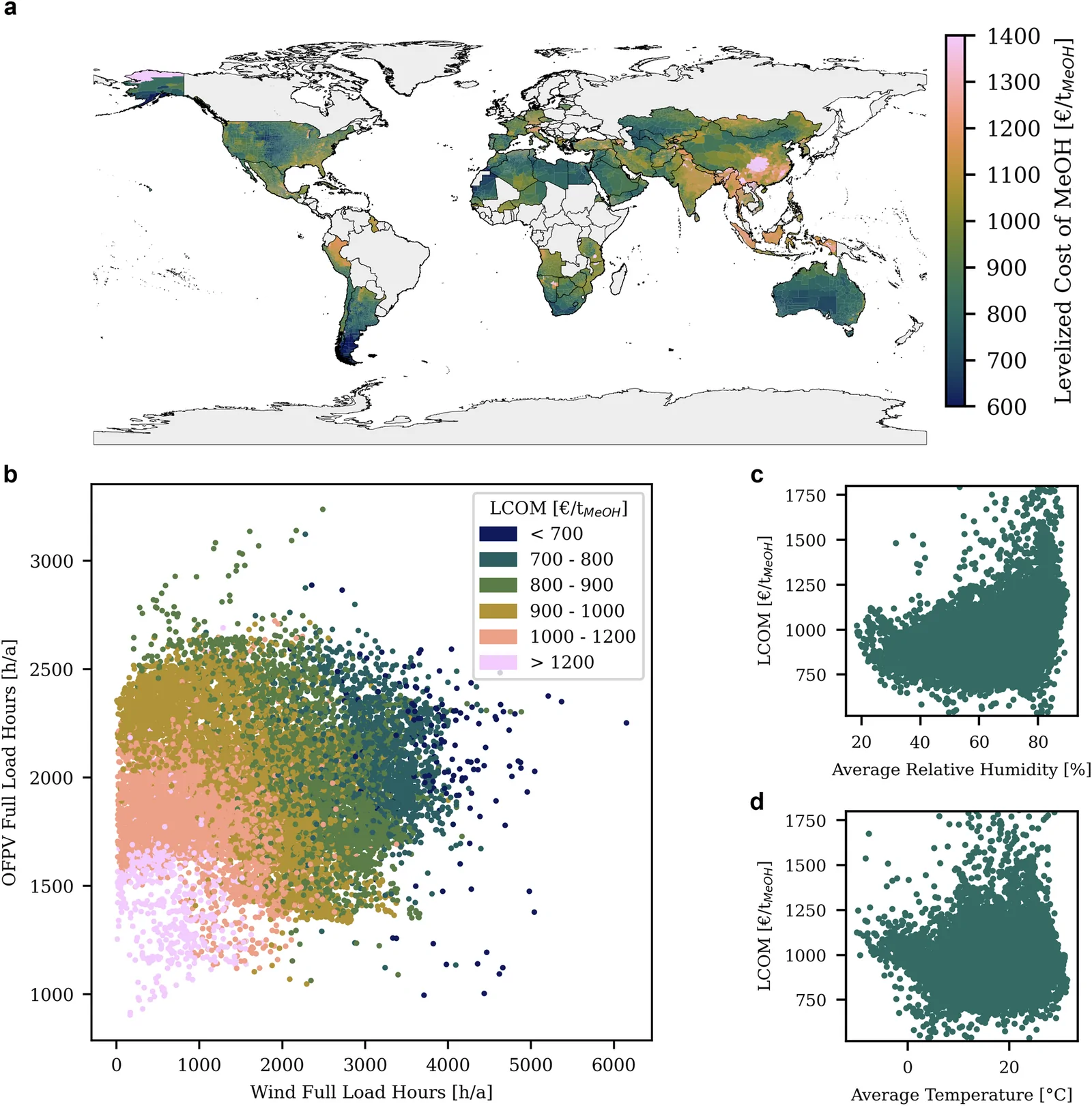
Levelized cost of water-conscious green methanol production (LCOM) by 2050 at 10% of the maximum technical potential. The maximum technical potential is derived by utilizing all available renewable energy potential for methanol production (see Methods). **a** Spatial distribution of the LCOM worldwide. The considered regions are expected to face significant levels of water-stress by 2050^13^, making water-conscious production essential. All regions with LCOM greater or equal to 1400 € t_MeOH_^−1^ are colored the same, as are all regions with cost less than or equal to 600 € t_MeOH_^−1^. **b** Analysis of the influence of open-field photovoltaic (OFPV) and wind full load hours (FLH) on the LCOM. The FLH of OFPV and wind plants are evaluated based on the utilized input supply profiles. **c**, **d** Analysis of the effect of average relative humidity and temperature on LCOM. The yearly average temperature and relative humidity are derived from the hourly resolved weather input data for the considered weather year 2018. A scientifically developed color scheme^85^ is used in this and the following figures to fairly represent the data and to make the figures universally readable. Country shapes from GADM^26^.

The geospatial analysis of over 20,000 regions reveals a wide range of levelized cost for water-conscious green methanol production by 2050, from ~537 € t_MeOH_^−1^ in favorable regions with low production cost to over 2000 € t_MeOH_^−1^ in more expensive regions. In half of the evaluated regions, levelized cost of methanol (LCOM) is estimated at 976 € t_MeOH_^−1^ or lower, while only 165 and 22 regions achieve LCOM below 700 and 600 € t_MeOH_^−1^, respectively. Low-cost production potential is identified across all continents (Fig. 2a), though costs below 600 € t_MeOH_^−1^ are exclusively found in the USA, Argentina, Chile and Northwestern Africa (Mauritania & Morocco). Additional regions with production costs below 700 € t_MeOH_^−1^ are found in various regions across the globe including, but not limited to, South Africa, Spain and Australia. Overall, the analysis of geospatial distribution reveals no distinct geographical clusters.

The assessment of input data effects on LCOM reveals the full load hours (FLH) of the energy supply system as the most influential factor, with wind FLH being decisive for reaching the lowest LCOM. Regions with both low wind and OFPV FLH exhibit the highest production costs, while an increase in OFPV FLH can significantly reduce LCOM, reaching values as low as 800 € t_MeOH_^−1^. However, regions with poor wind resources are not able to reach the lowest production costs, independent of the OFPV FLH. Only when wind FLH exceed ~2500 h a^−1^, LCOM in a range of 700–800 € t_MeOH_^−1^ are observed. The lowest production costs are predominantly found in regions benefiting from both high wind and OFPV FLH, particularly those exceeding 3000 wind FLH and 1750 OFPV FLH (Fig. 2b). In contrast, the influence of average relative humidity and ambient temperature on LCOM is generally less severe (Fig. 2c, d). Although the highest LCOM values are observed in regions with particularly high relative humidity, some of the lowest costs also occur under such conditions. A slight trend suggests that increasing relative humidity tends to lead to higher LCOM, which can be attributed to the increased energy demand of DAC in humid regions, as detailed in Fig. 9 and further analyzed later.

**Fig. 3:**
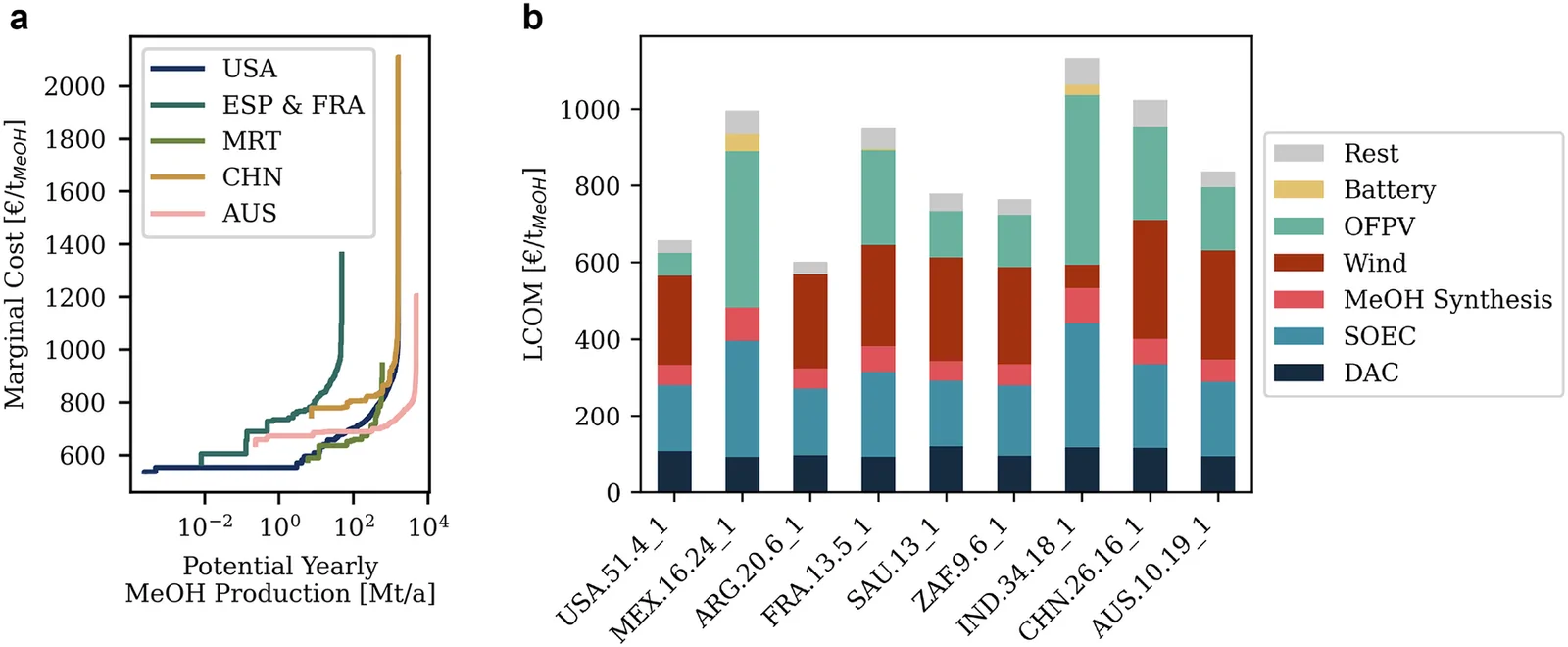
Specific analysis of selected regions. **a** Supply curves showing the (cost-) potential for six countries. **b** Cost contributions of the different system components to the total LCOM in nine regions across the globe (USA – United States of America, MEX – Mexico, ARG – Argentina, FRA – France, SAU – Saudi Arabia, ZAF – South Africa, IND – India, CHN – China, AUS – Australia, ESP – Spain, MRT – Mauretania). The regions are encoded according to the second administrative level, as defined in the global administrative boundaries dataset^26^. SOEC Solid Oxid Electrolysis Cell, OFPV open-field photovoltaic, DAC direct air capture.

Several countries exhibit significant potential for water-conscious green methanol production exceeding 1 Gt_MeOH_ a^−1^, such as the USA, China and Australia (Fig. 3a). A global demand of 500 Mt_MeOH_ a^−1^, as forecasted for 2050^11^, can be met at average production costs of 604 € t_MeOH_^−1^. Figure 3a presents merit order curves for three regions with particularly low production costs – USA, Mauritania (MRT), and Australia (AUS) – as well as China (CHN), which currently accounts for 65% of global methanol demand, with further growth expected^10^. Spain (ESP) and France (FRA) are included to assess the potential for domestic European production. Potential in Spain and France is limited to about 48 Mt_MeOH_ a^−1^ at 10% of the maximum technical potential, with an average cost of 872 € t_MeOH_^−1^, making them the least competitive among the selected regions. In contrast, Australia has the highest potential, reaching about 4.8 Gt_MeOH_ a^−1^ at 10% of the maximum technical capacity. It is found that Australia alone could meet the projected global methanol demand of about 500 Mt_MeOH_ a^−1^ by 2050^11^ at an average cost of 693 € t_MeOH_^−1^ and a marginal cost of 700 € t_MeOH_^−1^. However, considering all regions, the projected demand of 500 Mt a^−1^ could be supplied at an average cost of 604 € t_MeOH_^−1^ (Supplementary Note 2). The USA and China are found to have a comparable production potential, though costs are generally lower in the USA. Among the investigated regions, Mauritania emerges as the most cost-competitive location, with marginal (average) cost of 656 (636) € t_MeOH_^−1^ at production of 100 Mt_MeOH_ a^−1^.

The cost contribution of the process chain – including DAC, SOEC and methanol synthesis – varies between about 300 € t_MeOH_^−1^ in a selected US region to over 500 € t_MeOH_^−1^ in an Indian region (Fig. 3b). While the cost contribution of the DAC unit remains relatively consistent across regions, the SOEC contribution shows significant variation, suggesting substantial oversizing in some regions. The cost of the energy supply system, comprising OFPV and onshore wind, varies significantly depending on the region, ranging from 200 to 500 € t_MeOH_^−1^. In most cases, hybrid OFPV and wind systems are implemented to minimize costs. However, notable exceptions include the selected region in Mexico where wind potential is absent, leading to an exclusively OFPV-powered system.

In OFPV-dominated regions, substantial oversizing of the SOEC and methanol synthesis unit is observed. Generally, battery storage is most implemented in OFPV-dominated regions. When the share of installed PV capacity exceeds 80% of the total power supply capacity, battery storage deployment increases significantly, along with notable SOEC oversizing (Supplementary Fig. 13). The methanol synthesis unit is typically oversized in regions where SOEC oversizing occurs. This is attributed to the assumed flexibility of the system, where operating the energy-intensive electrolysis during periods of high power availability and shutting it down during periods of low supply proves to be the most cost-effective strategy. Due to the high cost and energy demand of syngas compression and storage, the SOEC and methanol synthesis units operate in a coupled manner. In wind-rich regions, all system components generally follow wind power availability, with additional operational peaks during OFPV generation periods (Supplementary Fig. 17a, c). Large-scale battery storage is not found to be economically viable under any conditions. If built, battery storage is primarily used to increase the capacity factor of the DAC unit (Supplementary Fig. 17b). While the DAC unit is capital intensive and an increased capacity factor lowers costs, this might also be explained by potentially less energy-intensive conditions for DAC operation during times of low or no power supply, or by the need to shift DAC operation due to water co-adsorption restrictions. These aspects are more deeply investigated in the following section.

### Weather dependency of direct air capture

Since the only material input to DAC is ambient air, its energy requirements and costs depend strongly on atmospheric conditions such as CO_2_ concentration, pressure, temperature, and humidity^22,27–29^. These parameters vary substantially across both space and time, with pronounced fluctuations not only seasonally but also on an hourly scale. Thus, in this analysis, we account for hourly variations in temperature and humidity at each location. While site-specific studies have demonstrated the value of incorporating hourly CO_2_ concentration dynamics^29^, such data is not yet available on a global scale. We therefore begin by examining how weather impacts DAC performance and system costs, before investigating its implications for water supply under worldwide weather variability.

**Fig. 4:**
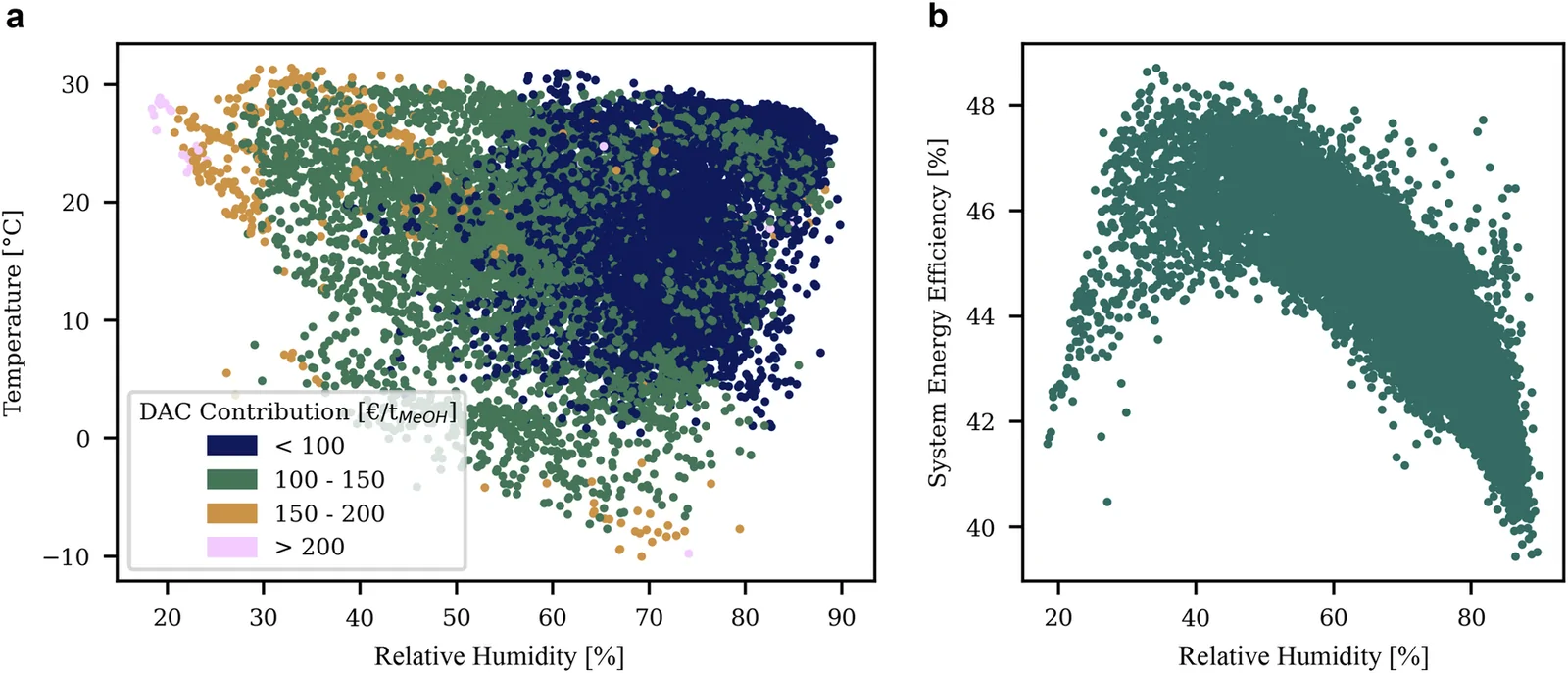
Influence of the prevailing weather conditions on the direct air capture (DAC) unit. **a** DAC cost contribution (without energy costs) to the total levelized cost of methanol (LCOM). **b** Influence of the relative humidity on total system energy efficiency. As the DAC plant is the only component of the process chain directly affected by weather conditions, the variations in system efficiency are mainly attributed to the DAC unit.

Varying weather conditions significantly impact the cost contribution of the DAC plant, with high relative humidity leading to contributions below 100 € t_MeOH_^−1^, while low-humidity conditions can increase contributions to over 200 € t_MeOH_^−1^ (Fig. 4a). This can be attributed to the higher productivity of the DAC plant under humid conditions, where water adsorption facilitates CO_2_ uptake (see Methods). The lowest cost contributions are observed in regions with average relative humidity above 60%, spanning a broad temperature range. In contrast, the highest costs are primarily found in regions with low average humidity (<30%) and high temperatures (>20 °C). This is a direct result of reduced DAC productivity under such conditions (Fig. 9), necessitating significant oversizing of the DAC unit in these regions. Additionally, elevated costs are observed in regions with relative humidity around 70% and temperatures below −5 °C. However, since these conditions do not negatively impact DAC productivity, the oversizing in these regions cannot be attributed to the weather dependency of the DAC plant and is instead explained by poor conditions for the renewable energy supply system.

The overall energy efficiency of the system is strongly influenced by relative humidity, which significantly impacts energy demand of the DAC unit (Fig. 4b). Total system efficiencies range from 39% to 49% (based on the lower heating value of methanol), emphasizing the importance of modeling DAC’s weather dependency. Since the energy demand of other process chain components is not affected by the prevailing weather conditions, variations in system efficiency are primarily driven by the fluctuating energy demand of the DAC unit. Other factors, such as the efficiency of the air-source heat pump, have only a minor effect. In regions with average relative humidity below 25%, low system efficiencies in a range of 41–43% are reached. This is primarily due to the elevated temperature in these regions which leads to an increased power demand for DAC operation, particularly for fans and vacuum pumps (Fig. 9). As relative humidity increases, system efficiency generally improves, peaking in regions with average relative humidity between 30% and 60%. However, beyond 60%, a clear trend emerges where higher relative humidity correlates with decreasing efficiency. The lowest observed system efficiency, ~39.5%, occurs in a region where the average relative humidity approaches 90%. This is explained by the substantial increase in DAC’s heat demand at higher relative humidities (Fig. 9a). While the geospatial analysis reveals clear relationships between weather conditions, cost contributions and system efficiency, these observations should primarily be interpreted as correlations rather than direct causal relationships. The proposed explanations are based on the underlying process mechanisms represented in the model; however, multiple interdependent factors simultaneously influence system design and operation, meaning that individual parameter effects cannot always be isolated clearly. This limitation should also be considered in the following assessment of water supply from DAC.

To produce the required synthesis gas for methanol synthesis, the SOEC needs water and CO_2_ as a feedstock, both directly provided from ambient air by the DAC unit. However, as discussed in the Methods section, a water supply ratio below 0.841 t_H2O_ t_CO2_^−1^ leads to insufficient water availability for synthesis gas production and, consequently, methanol synthesis (Fig. 9c, e). Notably, only 590 out of the 20,265 analyzed regions are affected by an average water supply ratio below 0.841 t_H2O_ t_CO2_^−1^ (Fig. 9e). Although DAC plants in these regions would be unable to supply sufficient water if operated continuously throughout the year, they nonetheless experience periods with desorption ratios exceeding 0.841 t_H2O_ t_CO2_^−1^ (Supplementary Fig. 8). Since no alternative water source is available in the analyzed system, a shortfall in water supply necessitates either drawing from buffer storage or shifting DAC operation to periods with sufficiently high water co-adsorption. In any case, the DAC unit must provide the total water requirement for methanol production over the course of a year. To assess potential operational constraints related to DAC water supply, the yearly excess water production per produced unit of methanol is analyzed.

**Fig. 5:**
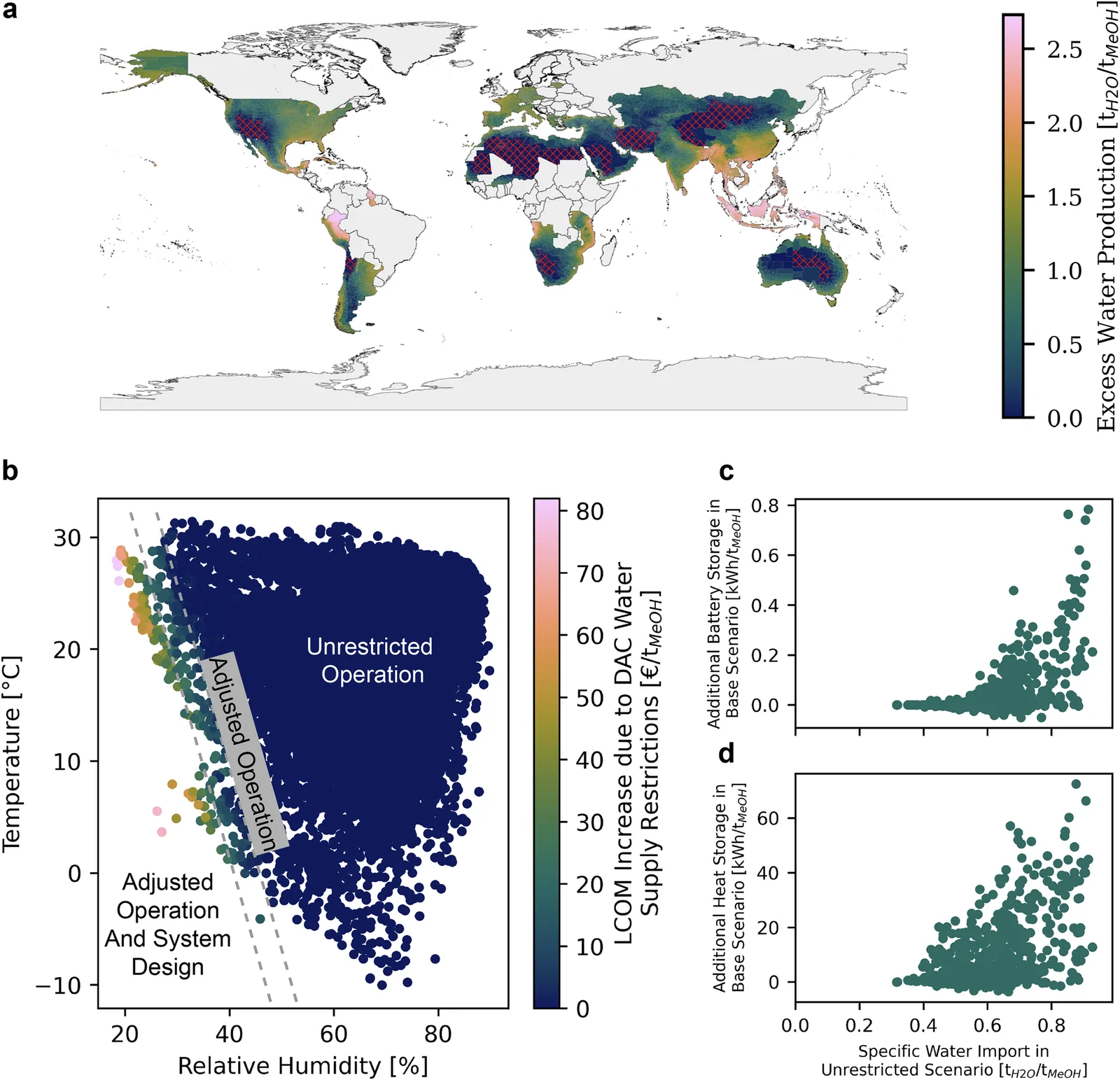
Analysis of the water supply by direct air capture (DAC) and the effect of insufficient water supply on the system. **a** Specific excess water production in all considered regions. Regions with no excess water production are indicated by red hatches. **b** Analysis of the levelized cost of methanol (LCOM) increase due to water supply restrictions. To quantify the increase, regions with zero excess water production were again optimized, this time in an unrestricted scenario allowing for free water import. **c**, **d** Additional built battery and heat storage to overcome water supply restrictions. Country shapes from GADM^26^.

In most regions, water supply by DAC does not impose constraints on water-conscious green methanol production, and excess water production is generally observed. In these regions, an additional water source, i.e. seawater desalination, would not have changed the system design, operation and resulting cost, since sufficient water from DAC operation is available anyway. Therefore, these regions are considered suitable for DAC-based production, as the water demand can be fully met through atmospheric capture without additional water-related constraints. The highest excess water production, exceeding 2.5 t_H2O_ t_MeOH_^−1^, is found in Indonesia and Peru (Fig. 5a), driven by the high relative humidity in these regions (Supplementary Fig. 7b). Regions with low excess water production are distributed globally, including parts of China, Chile, Africa and the USA. In regions where excess water production is exactly zero, system design and operational adjustments – such as building additional energy storage and shifting DAC operation to periods of higher water co-adsorption – may be necessary to ensure sufficient water supply, given that no other water source is available in the investigated system. Among the 20,265 analyzed regions, 606 show no excess water production, primarily located in the USA, Northern Africa, the Middle East and Australia (Fig. 5a). These 606 regions are nearly identical to the 590 regions where the average water co-adsorption ratio of a continuously operated DAC plant is below 0.841 t_H2O_ t_CO2_^−1^ (Fig. 9e). Additionally, some regions with a theoretically sufficient average water supply ratio are affected, since cost-optimal DAC operation deviates from continuous operation and often is observed to be during times of high power supply and lower water co-adsorption, e.g. during daytime in summer. To quantify the impact of water supply constraints, these 606 regions were again optimized, this time in an unrestricted scenario, allowing for free water import. In the following, the resulting system configurations and LCOM values from this scenario are compared to the base scenario to evaluate potential differences.

Regions experiencing extremely low levels of relative humidity, especially in combination with high temperatures, are potentially less suited for water-conscious green methanol production with LCOM increases up to 80 € t_MeOH_^−1^ resulting from water supply constraints (Fig. 5b). Notably, regions with high LCOM increases due to water supply restrictions are exclusively landlocked regions far from the shore, such as southern Algeria, northern Niger, Afghanistan or northern China (Supplementary Fig. 14). Three distinct operational zones for DAC-based water supply can be revealed (Fig. 5b). In most regions, average temperature and relative humidity do not impose significant constraints, allowing unrestricted operation. A second zone, where only a slight LCOM increase (<20 € t_MeOH_^−1^) is observed, corresponds to regions with relative humidity levels of ~30–35% at 20 °C or 40–45% at 0 °C. Here, DAC can generally provide sufficient water, requiring only minor operational adjustments and system design modifications. In contrast, regions with even lower relative humidity at these temperature levels face severe constraints on DAC water supply. This necessitates substantial system design adaptations and operational shifts, especially increased battery and heat storage capacity (Fig. 5c, d), leading to significantly higher LCOM. These system modifications enable a shift in DAC operation to periods of higher relative humidity, ensuring sufficient water adsorption. As a result, areas experiencing these specific temperature and relative humidity conditions may be less suitable for water-conscious green methanol production enabled by solid sorbent DAC.

### Water supply remains robust to DAC model choice

Multiple DAC process models have been developed by research groups worldwide, differing in sorbent selection, process design, and scope of investigation. However, only a limited number of studies report comprehensive data on energy demand, productivity and, most importantly, water desorption ratio under a wide range of ambient conditions^22,28,30^. Given the differences in modeling approaches and underlying assumptions, the choice of DAC model can substantially influence overall system performance. This is particularly relevant since the DAC plant supplies all necessary feedstocks for downstream processes – variations in water co-adsorption behavior might therefore render the entire system infeasible. To investigate the influence of DAC model selection on the system performance, we utilize data from a second, alternative DAC model^28^ and integrate this into the system under investigation (see Methods). By keeping all other system components constant and re-optimizing the total system for all 20,265 regions, the influence of DAC model choice can be evaluated. The results of this alternative scenario, from here on referred to as the altDAC scenario, are compared against the base scenario.

**Fig. 6:**
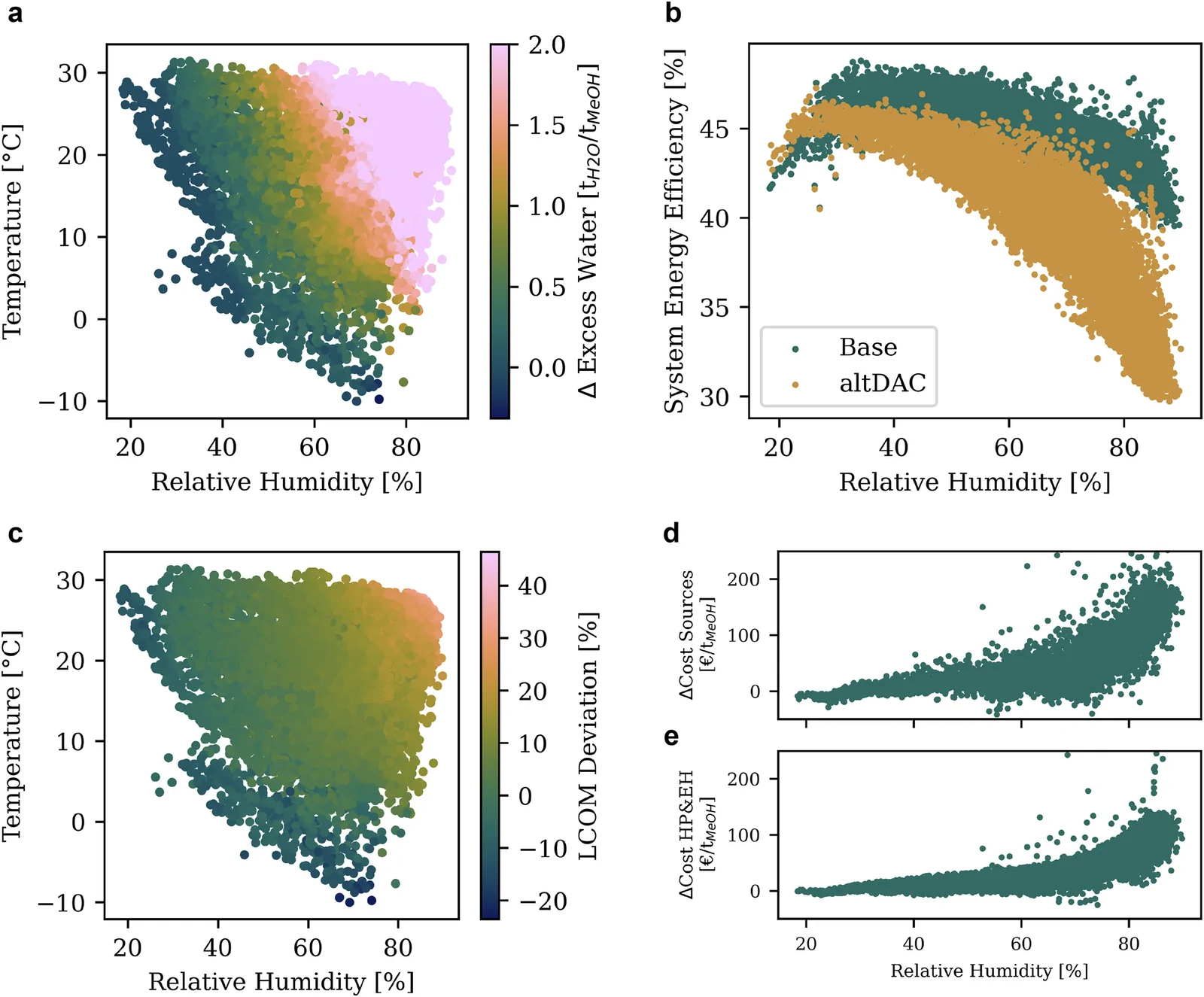
Evaluation of direct air capture (DAC) model choice on system performance and feasibility. The scenario with an alternative DAC model (altDAC) is compared against the base scenario. **a** Additonal excess water production in the altDAC scenario compared to the base scenario at varying ambient conditions. **b** Total system efficiency of the altDAC and base scenario depending on average relative humidity. **c** Deviations in resulting levelized cost of methanol (LCOM) when comparing altDAC and base scenario. **d**, **e** Additional cost contributions by different system components (Sources include wind & open-field photovoltaic plants, HP includes all heat pumps and EH all electric heaters) in the altDAC scenario.

In the altDAC scenario, substantial additional excess water production is observed relative to the base scenario across nearly all regions and ambient conditions (Fig. 6a). Only 13 out of the 20,265 regions exhibit lower excess water production relative to the base scenario, all located at high elevations in central Asia. These regions experience high average relative humidity (60–80%) at low average temperatures approaching −10 °C, yet they are not subject to insufficient water supply. Regions facing insufficient water supply in the base scenario generally experience no or a reduced shortfall when the alternative DAC model is applied. Specifically, while 606 regions exhibit potential water insufficiency in the base scenario, only 261 regions show zero excess water production in the altDAC scenario. A comparison of the weather-dependent water desorption ratio of the base DAC model (Fig. 9) and the alternative DAC model (Supplementary Fig. 11) confirms that the alternative DAC model consistently supplies more water from ambient air. Consequently, the proposed system remains feasible and direct water supply from ambient air via DAC for hydrocarbon production is validated as a robust approach.

Nevertheless, a comparison of total system efficiency and the resulting LCOM for both scenarios emphasizes the significant impact of DAC model choice. At low relative humidity, particularly in combination with elevated temperatures, the alternative DAC model requires less heat (Supplementary Fig. 11), resulting in an overall higher system efficiency and lower LCOM (Fig. 6b, c). However, at higher relative humidity, the heat demand of the alternative DAC model increases severely, resulting in an overall reduction of the total system efficiency (Fig. 6b, Supplementary Fig. 11). The reduced system efficiency at higher relative humidity translates into a significant increase in LCOM, reaching ~40% under extreme conditions (Fig. 6c). The additional cost is primarily driven by the need for greater energy input from renewable sources (wind and PV) and by the integration of additional conversion technologies, including heat pumps and electric heaters (Fig. 6d, e). Despite these substantial deviations under extreme ambient conditions, more than 71% of regions exhibit LCOM variations of less than 15%.

The pronounced differences between the two DAC models can largely be attributed to the substantially higher predicted water uptake and higher resulting heat demand of the alternative model compared to the base DAC model. The reasons for these deviations are manifold and cannot be attributed to a single factor, since both DAC models are based on different sorbents, adsorption formulations, and process assumptions. In particular, the alternative DAC model^28^ employs humidity-dependent reaction mechanisms and adsorption kinetics for a proprietary amine-functionalized sorbent^28,31^. In contrast, the base scenario model^22^ applies a co-adsorption model^32^, where adsorbed water directly influences the equilibrium CO_2_ uptake of Lewatit^22^. In addition, the operational strategies of both studies differ considerably. Cai et al. evaluate DAC performance using fixed cycle times under all ambient conditions^28^, whereas Jajjawi et al. optimize adsorption and desorption durations as a function of temperature and humidity to minimize the specific energy demand. As discussed by Cai et al., fixed cycle times under humid conditions lead to incomplete desorption steps, reducing effective CO_2_ productivity^28^. This results in higher water-to-CO_2_ ratios and substantially larger specific heat demands. The base model, in contrast, can partially compensate for unfavorable adsorption conditions through dynamic cycle adaptation, reflected by longer desorption durations under humid conditions^22^, leading to a lower co-adsorption ratio and specific heat demand.

Overall, the observed differences reflect the sensitivity of DAC performance predictions to the underlying sorbent properties, co-adsorption formulations, and operational assumptions. Importantly, despite the considerable deviations in energy demand and LCOM under extreme climatic conditions, the central conclusion of this work remains robust: both DAC models consistently indicate that DAC-based water supply is sufficient for water-conscious methanol production across a wide range of regions and environmental conditions.

### Air cooling as a water-conscious opportunity

To enable water-conscious methanol production, a cooling system that operates without water consumption is crucial, besides supply of the water feedstock. Generally, waste heat is primarily generated in the SOEC and methanol synthesis unit, totaling in about 1.34 MWh t_MeOH_^−1^. This heat must either be integrated into the process, i.e. by upgrading it via a heat pump for use in the DAC unit, or dissipated through an air cooling system (see Methods).

**Fig. 7:**
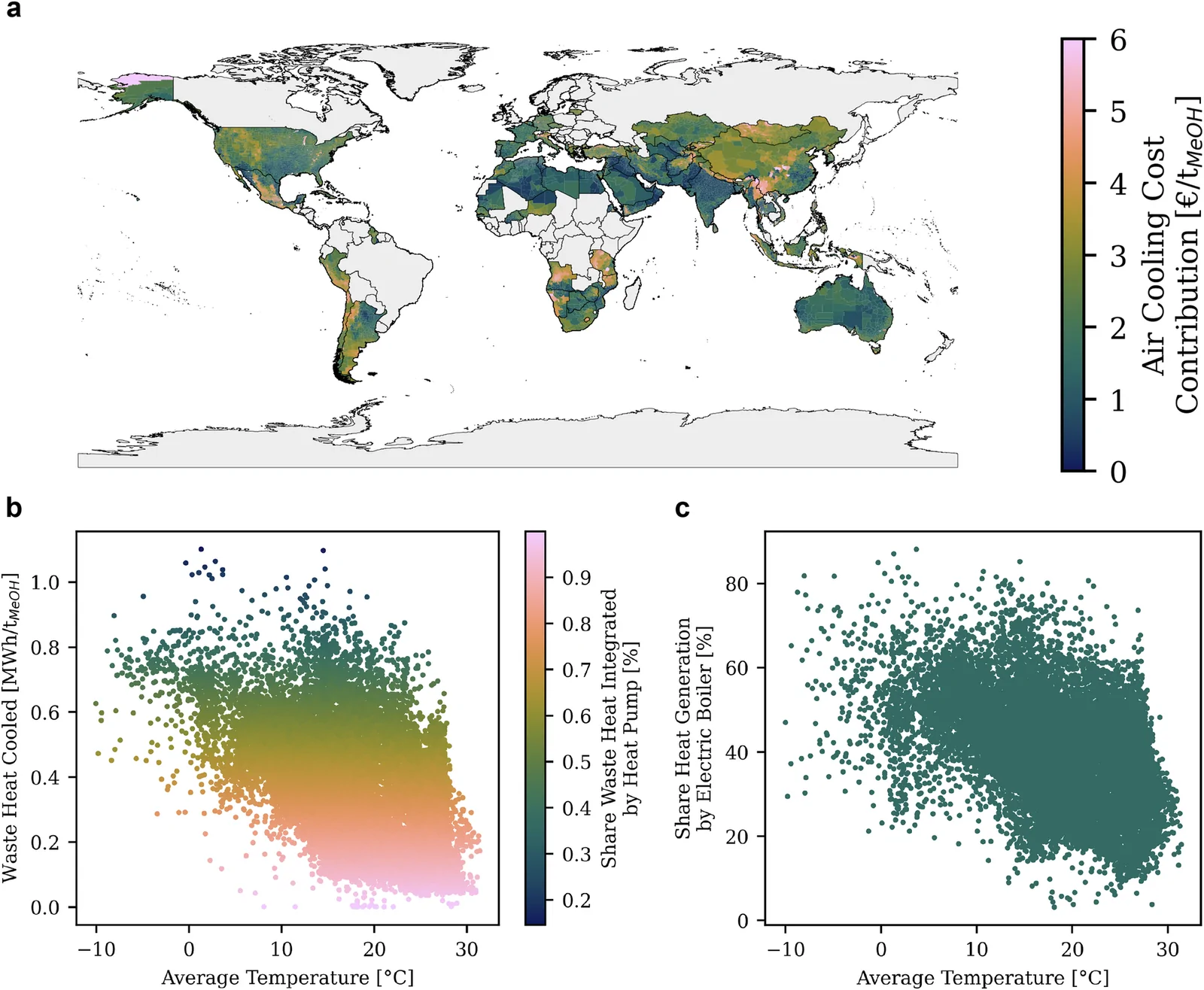
Analysis of the air cooling and heat supply system. **a** Cost contribution of the air cooling system (capex and fixed opex, without energy cost) to the total levelized cost of methanol (LCOM). All regions with cost contribution greater than or equal to 6 € t_MeOH_^−1^ are colored the same. The highest observed cost contribution is about 30 € t_MeOH_^−1^. **b** Analysis of the waste heat pathways in all considered regions dependent on the average ambient air temperature. Besides being integrated by a heat pump, the waste heat could also directly be used for CO_2_ gasification. **c** Share of the electric boiler in total heat generation. Country shapes from GADM^26^.

Air cooling has only a minor impact on the total LCOM and appears to be a promising cooling solution (Fig. 7a). Overall, the cost contribution remains below 5 (3) € t_MeOH_^−1^ in 18,538 (14,200) out of the 20,265 analyzed regions. Notably, the lowest cost contributions are observed in Northern Africa and the Middle East. While these regions experience high average air temperatures (Supplementary Fig. 7a), which could suggest a need for increased air cooling capacity (see Methods), the opposite is observed. Instead, less waste heat is dissipated through air cooling, and a larger share is integrated into the process via a heat pump (Fig. 7b). This indicates that in regions with elevated temperatures, waste heat is preferentially upgraded and utilized rather than cooled, as the efficiency and cost-effectiveness of the air cooling system declines at higher temperatures (see Supplementary Methods). In some regions, more than 90% of the total generated heat is from upgraded waste heat, demonstrating that a substantial portion of the DAC unit’s heat requirement – ranging from 1.5 to 2.7 MWh t_MeOH_^−1^, depending on the region – can be covered by waste heat integration. In colder regions, generally, a significant share of total heat generation comes from electric boilers (Fig. 7c). In these cases, a combination of air cooling and electric boilers is more cost-effective than utilizing waste heat pumps, as the air cooling system operates efficiently at lower temperatures, and electric boilers have substantially lower capital costs compared to heat pumps. The lower efficiency of electric boilers does not present a major drawback, as they are primarily operated during periods of high power supply (Supplementary Figs. 17, 18). To balance heat supply and waste heat integration, relatively large heat storage is used (Supplementary Fig. 18).

### Sensitivity analysis and alternative process chains

**Fig. 8:**
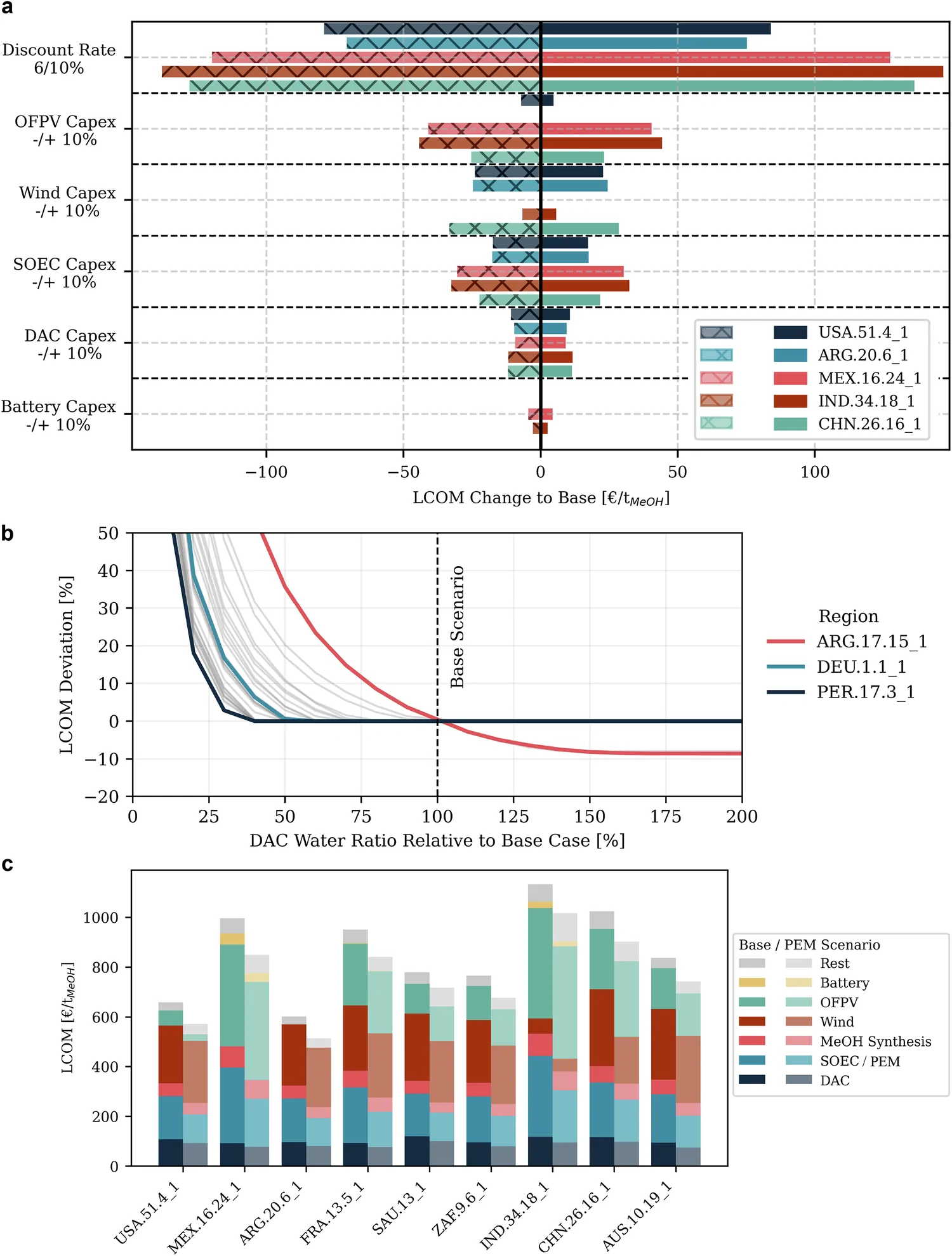
Sensitivity analysis for the investigated system. **a** Variation of input parameters and resulting differences in levelized cost of methanol (LCOM) compared to the base case. The capex of key system components, such as open-field photovoltaic (OFPV), onshore wind, direct air capture (DAC), solid oxide electrolysis (SOEC), and battery, were varied by ±10%. Additionally, the impact of different discount rates was evaluated, with scenarios considering a discount rate of 6% and 10%, compared to the base discount rate of 8%. Sensitivity analysis was performed across five distinct regions. Three of these regions are powered by hybrid OFPV-wind systems (USA.51.4_1, IND.34.18_1, CHN.26.16_1), one region relies exclusively on OFPV (MEX.16.24_1), and one region is solely powered by wind (ARG.20.6_1). **b** Variation of the DAC water supply ratio and influence on the resulting LCOM. The regions with the lowest average water ratio (ARG.17.15_1) the one with the highest (PER.17.3_1) and one with a moderate ratio (DEU.1.1_1) are colored. Additionally, 30 regions with varying humidity are depicted in gray to highlight the variance. **c** Comparison to the alternative process chain with polymer electrolyte membrane (PEM) electrolysis and direct CO_2_-hydrogenation for selected regions (USA – United States of America, MEX – Mexico, ARG – Argentina, FRA – France, SAU – Saudi Arabia, ZAF – South Africa, IND – India, CHN – China, AUS – Australia). The regions are encoded according to the second administrative level, as defined in the global administrative boundaries dataset^26^.

Figure 8a illustrates the influence of changes in various parameters on the resulting LCOM compared to the base case. The discount rate has the most significant impact, with changes of ~150 € t_MeOH_^−1^ for the corresponding region in India and around 75 € t_MeOH_^−1^ for the selected region in Argentina. As anticipated, regions with higher overall costs are more significantly affected by changes in the discount rate. The second strongest influence comes from changes in the capital expenditures (capex) of OFPV. Interestingly, the region exclusively powered by OFPV (MEX.16.24_1) is not the most sensitive to changes in the capex of OFPV units. While a ± 10% change in OFPV capex results in a shift of about ±40 € t_MeOH_^−1^ for this region, it causes a ± 45 € t_MeOH_^−1^ shift in the Indian region. This is explained by the high share of OFPV in IND.34.18_1, combined with fewer FLH than in MEX.16.24_1 (Supplementary Fig. 9), which requires more installed capacity. A change in wind capex leads to LCOM variations of up to ±30 € t_MeOH_^−1^ across the analyzed regions, with CHN.26.16_1 experiencing the greatest impact – greater than the exclusively wind-powered region in Argentina – again due to the difference in FLH. Changes in the capex of SOEC lead to LCOM variations of 15 to 30 € t_MeOH_^−1^, while changes in DAC capex result in relatively consistent LCOM differences of about 10 € t_MeOH_^−1^. The smaller variation between regions can be attributed to the generally lower oversizing of DAC units, as discussed above. Finally, changes in the capex of the battery affect only OFPV-dominated regions, with relatively minor effects on LCOM, about 5 € t_MeOH_^−1^.

In addition to the capex sensitivities shown in Fig. 8a, a separate sensitivity analysis was conducted to assess the influence of potentially increased maintenance and replacement requirements by increasing the annual operational expenditures (opex) of both DAC and SOEC systems from 4% in the base scenario to 6%. For SOEC systems, the increased opex can, for instance, be interpreted as a simplified representation of higher stack replacement costs resulting from intermittent operation and associated degradation. For DAC systems, it may reflect increased maintenance requirements under harsh environmental conditions, such as enhanced filter maintenance due to dust exposure. The results show a comparatively moderate impact on LCOM, with increases of ~1–2% for higher DAC opex and 3–4% for higher SOEC opex. While these effects are not negligible, they indicate that even increased maintenance and replacement requirements remain economically manageable within the investigated system configurations.

Given the study’s focus on evaluating water supply from DAC under varying ambient conditions and the associated uncertainties, Fig. 8b shows the effect of reduced or increased water-to-CO_2_ ratios on the resulting LCOM across multiple regions. In the region with the lowest average water ratio in the base scenario (ARG.17.15_1, 0.48 t_H2O_ t_CO2_^−1^), reducing the water supply ratio to 75% of the base case increases the LCOM by about 12% due to water constraints and the required system adaptations discussed above. In contrast, the humid region with the highest average water ratio (PER.17.3_1) remains unaffected even when the water ratio is reduced to 40% of the original value. Regions with moderate climates, such as DEU.1.1_1 in central Europe, are also insensitive to substantial reductions in the water ratio. Overall, most regions maintain sufficient water supply even under reduced water ratios, supporting the potential of DAC-based water supply despite uncertainties in future developments.

Besides changes of single parameters, the system under investigation is compared against an alternative process chain, consisting of polymer electrolyte membrane (PEM) electrolysis in combination with direct CO_2_-hydrogenation. This combination is often found in scientific literature^7,18^ and might be a relevant alternative, especially considering the higher maturity and lower capital cost of PEM electrolysis compared to SOECs^33^.

The PEM-based system achieves lower LCOM in all regions, particularly in regions dominated by OFPV, where reductions of up to 150 € t_MeOH_^−1^ are observed (Fig. 8c). The primary reason for these significant reductions in cost is the cost contribution of PEM electrolysis compared to SOEC. This is attributed to the generally lower specific capex of PEM electrolysis, as well as the reduced capacity required, given that PEM electrolysis only performs water electrolysis, unlike SOEC, which performs co-electrolysis necessitating more installed capacity. Additionally, the DAC unit contributes less to the total LCOM in the PEM-based system, as the required capacity is lower. This is because the direct CO_2_-hydrogenation unit requires less carbon input compared to the CO-hydrogenation process used in the SOEC-based system (see Methods). Besides the obvious cost reduction potential, it should be noted that large-scale CO_2_-hydrogenation (>500 kt a^−1^) has not yet been implemented^12^. While the PEM-based process seems promising, the significantly higher hydrogen-to-carbon ratio required for direct CO_2_-hydrogenation could lead to a substantially increased needed water ratio of up to 1.23 t_H2O_ t_CO2_^−1^, potentially resulting in water supply restrictions. Additionally, PEM electrolysis requires ultra-pure water^34^, necessitating additional purification steps. Although the associated energy demand and cost increase are relatively small – ~0.2% additional energy demand and 0.3–0.5% additional cost (Supplementary Fig. 16) – the purification process introduces further water losses, which can increase the overall LCOM in arid and water-constrained regions by more than 10% (Supplementary Fig. 16). Combined with the higher cooling demand of PEM electrolysis, this process chain may therefore be less suitable for water-conscious production in hot and dry climates. Consequently, while PEM-based production appears promising at first glance, these aspects require further detailed investigation to fully evaluate its potential.

## Discussion

In this study, we showed that water-conscious green methanol production enabled by solid sorbent DAC is feasible across most water-stressed regions globally. In the following, we will discuss production costs in a broader context, highlighting the influence of weather dependency and some further noteworthy aspects before concluding with limitations and an outlook.

The expected production costs span a broad range, with global methanol demand in 2050 potentially being met at costs as low as 604 € t_MeOH_^−1^. This estimate falls within the more optimistic range of prior assessments (~400–800 € t_MeOH_^−1^)^17,35–37^, yet remains more conservative than a recent projection of 315–370 € t_MeOH_^−1^ by 2050^6^. However, it is important to note that no prior study has accounted for the substantial influence of weather variability on DAC performance or examined water-autarkic systems. For context, fossil-based methanol production costs have historically ranged between 100 to 250 $ t_MeOH_^−1,^ ^10^, although recent price spikes up to 800 $ t_MeOH_^−1^ have been observed^38^. In 2024, for example, methanol prices were ~300 $ t_MeOH_^−1^ in Asia, ~550 $ t_MeOH_^−1^ in Europe, and in the US rose from ~600 $ t_MeOH_^−1^ in February to over 800 $ t_MeOH_^−1^ in December^38^. These recent trends suggest that green, water-conscious methanol production is approaching economic viability – particularly as fossil-based methanol costs are likely to increase further under tightening CO₂ pricing. An alternative pathway could involve continued fossil-based methanol production combined with direct air carbon capture and storage (DACCS) for emission offsetting, as recently discussed for SAFs^3^. However, this route appears economically unattractive. Fossil methanol production generates emissions of 0.5–1.5 t_CO2_ t_MeOH_^−1^ during synthesis, with combustion adding another 1.375 t_CO2_ t_MeOH_^−1^, for a total emission burden of 1.875–2.875 t_CO2_ t_MeOH_^−1,^ ^6^. At projected DACCS costs of about 340 $ t_CO2_^−1^ by 2050^1^, offsetting these emissions alone would amount to 637–978 $ t_MeOH_^−1^, significantly higher than renewable production. Finally, although production costs are the primary cost driver, transportation costs must also be considered when matching supply and demand. A recent study shows that maritime shipping is a cost-effective option for methanol transport, adding ~23–41 € t_MeOH_^−1^ to methanol costs in 2050^39^. Additional costs for pipeline transport, for example between production sites and ports, are expected to be comparatively low, with reported costs of 2.3–3.5 € MWh^−1^ per 1000 km^39^.

By incorporating the weather dependency of DAC into our modeling, we find that water supply restrictions only arise in regions facing extremely low relative humidity, particularly in combination with elevated temperatures. Specifically, regions with an average annual relative humidity below 30% at 20 °C may face increased costs due to DAC water supply limitations. However, these challenges can often be mitigated through system adjustments, such as additional energy storage and shifting DAC operation to periods of higher relative humidity. Thus, solid sorbent DAC is generally capable of supplying sufficient water for methanol production. To validate the robustness of this finding, we conducted a detailed comparison with an alternative DAC model, which confirmed that solid sorbent DAC can reliably supply sufficient water for methanol production. This underscores its broader potential in enabling water-conscious synthesis of other carbon-based hydrogen derivatives, given that methanol is the hydrocarbon with the highest hydrogen-to-carbon ratio. Consequently, regions with high renewable energy potential are also well positioned for green hydrocarbon production, as all essential feedstocks (CO_2_ and H_2_O) can be directly captured from ambient air using solid sorbent DAC. Beyond water supply, the strong influence of local weather conditions on DAC performance significantly impacts both cost and system efficiency. Depending on location, the cost contribution of DAC ranges from below 100 € t_MeOH_^−1^ to above 200 € t_MeOH_^−1^, while system efficiency varies between 39% and 49%. These weather-induced variations can significantly affect production costs, underscoring the importance of integrating weather-dependent DAC modeling into assessments of green methanol production. Comparison with the alternative DAC model further demonstrates that model choice significantly affects both system efficiency and the resulting LCOM. Nevertheless, the impact of climate-dependent DAC operation has typically been overlooked in previous cost estimates of DAC-based fuels.

Our assessment of an air cooling system for water-conscious operation further supports the feasibility of the approach, as cooling-related costs have only a minor impact on total production costs. We find that at lower ambient temperatures, direct electric heating is more cost-effective than waste heat integration via heat pumps, leading to a preference for dissipating waste heat through air cooling. However, in regions with elevated air temperatures, air cooling becomes increasingly energy-intensive and expensive, making heat pump-based waste heat utilization the preferred strategy.

The conducted sensitivity analysis underscores the pronounced importance of the discount rate, which can vary significantly across regions^40^. Considering spatially resolved discount rates, based on political or environmental factors, could severely affect the resulting costs^41,42^, improving the reliability of techno-economic assessments. However, these variations are unlikely to affect the fundamental constraints of water supply and cooling, which were the primary focus of this study. Nevertheless, in future research these findings should be integrated. By comparing the system against an alternative process based on direct CO_2_-hydrogenation supplied by hydrogen from PEM electrolysis, the initial assumption that the high efficiency of SOECs is preferable was proven wrong. The slightly higher cost of the energy system is offset by the significantly lower cost of the process chain, resulting in lower LCOM for the PEM-based process. However, the higher hydrogen-to-carbon ratio required by the methanol synthesis unit in this pathway may pose challenges in terms of water supply, particularly in arid regions. Furthermore, large-scale deployment of PEM electrolysis involves considerable demand for critical materials, which may introduce additional restrictions^43^. These aspects highlight the need for further research.

Our comparison shows that DAC model selection can substantially influence both system efficiency and cost. However, we do not consider one DAC model to be inherently superior to another, as the employed models represent different process designs, sorbent materials, and underlying adsorption isotherm datasets. To enable more reliable cost assessments, advanced DAC models that incorporate state-of-the-art research – i.e., on weather-dependent mass transfer coefficients^28,44,45^ and validated adsorption behavior – are needed. At present, however, only a limited number of studies provide the necessary performance data across a broad range of ambient conditions^28^. We therefore emphasize the importance of evaluating DAC performance under diverse climatic settings and of publishing detailed process model data to enable independent integration into techno-economic assessments.

While our study provides a detailed system-level evaluation with a focus on DAC-enabled production, it does not account for the effects of intermittently switching electrolysis on and off. Frequent cold starts, in particular, could reduce SOEC lifetime due to accelerated degradation^46,47^. The flexible operation of the SOEC is therefore an optimistic assumption, and the resulting LCOM should be interpreted as not including potential additional replacement costs in regions with strongly intermittent operation. Future work should address these effects, for example by modeling the hot standby power requirements of SOEC systems or explicitly incorporating cold-start behavior.

Given the substantial heat demand and associated cost of heat pumps and electric heaters, future research should explore the integration of alternative renewable heat sources, such as geothermal energy or concentrated solar power, to assess the potential role of direct renewable heat integration. Additionally, integrating geothermal energy could enhance capacity factors through baseload operation^48,49^, potentially driving significant cost reductions. For instance, data from a previous assessments of global geothermal potential suggest that such integration could be feasible in more than 9400 of the 20,265 regions analyzed^49^, offering potential opportunities for reducing overall system costs.

Besides technology-specific limitations, our study applies uniform techno-economic assumptions across all regions to isolate the effects of weather conditions and renewable energy availability. Consequently, regional differences in labor costs, financing conditions and potential scale-dependent technology performance are not explicitly represented. In reality, economies of scale and regional economic conditions may affect both system cost and efficiency. Therefore, the presented results should primarily be interpreted as a comparative assessment of the feasibility of DAC-based feedstock provision across different regions.

Finally, enabling water-conscious methanol production expands the participation of water-stressed regions in the renewable energy transition. This approach not only ensures sustainable resource utilization but may also enhance public acceptance of large-scale projects, as excess water can be generated, and depletion of local freshwater resources is avoided. By addressing both water-conscious and green production, DAC-based production methods could play a key role in the global shift towards sustainable chemical and fuel production. Consequently, future studies should also investigate other DAC-based fuels, such as SAFs, in detail.

## Methods

### Techno-economic optimization

To determine the maximum potential and cost of water-conscious green methanol production, as well as the optimal system design, techno-economic optimization is employed. For each of the more than 20,000 considered regions, a single-node off-grid optimization problem is formulated by utilizing the ETHOS.FINE framework^25,50^ for energy system optimization. The below described process chain, energy supply system and all other components are translated into mathematical equations, serving as constraints during optimization. The optimization variables include the capacity of each component as well as the hourly operation rate. In a first step, the maximum technical potential, utilizing all available energy potential to produce methanol, is derived by maximizing the methanol production. In a second step, expansion shares of 5 and 10% of the maximum technical potential are exogenously set as the yearly methanol demand. A maximum of 10% was chosen, to account for electrification and potential production of other fuels or chemicals in each region. For the expansion shares, cost-optimization was performed for each region individually, utilizing the best available renewable energy potential and deriving the optimal capacity of each component, i.e. the installed capacity of the DAC unit, PV system and all other components, as well as the hourly optimal operation. During cost-optimization, the total annual system cost (TAC) is minimized. The levelized cost of methanol (LCOM) can then be derived by dividing the TAC by the total produced quantity of methanol, i.e. the sum of all operating variables of the methanol synthesis unit in each hour of the year ${{op}}_{{MeOH},t}$ (see Eq. (1)).

1 $${{\rm{LCOM}}}=\frac{{{\rm{TAC}}}}{{\sum }_{t=0}^{T}{{op}}_{{MeOH},t}}$$

For cost-optimization, a constant discount rate of 8% is applied. The influence of the discount rate is considered in a sensitivity analysis. All currency values have been converted to € and are inflation adjusted to January 2024 values by using the harmonized consumer price index of the European Central Bank^51^. The techno-economic assumptions of the main system components are listed in Table 1. All further assumptions, i.e. for storage or conversion components, are listed in the Supplementary Methods. All techno-economic parameters are representative for the year 2050, which is in the focus of this study.

**Table 1:** Techno-economic assumptions for the main system components based on the indicated sources

Technology	Capex	Fixed Opex (% of Capex)	Economic lifetime	Further aspects
DAC	316 € t^−1^a^17^	4%^17^	20 a^17^	Energy demand see Fig. 9
SOEC	776 € kW_el_^−1,^^17^	4%^17^	20 a^17^	Efficiency: 80% LHV^17^
CO-hydrogenation	177 € t^−1^a^17^	10%^17^	20 a^17^	Feedstock consumption see Process Chain Model
Horizontal single-axis tracking OFPV	611 € kW^−1,^^84^	2%^84^	30 a^84^	Potential and hourly capacity factors from prior analyses^14,21^
Onshore wind	893 € kW^−1,^^84^	2.5%^84^	30 a^84^	Potential and hourly capacity factors from prior analyses^14,21^

### Process chain model

The considered process chain model comprises a solid sorbent DAC plant, which supplies CO_2_ and H_2_O to a downstream SOEC for co-electrolysis, producing syngas (Fig. 1a). The syngas, supplemented with additional CO_2_, is subsequently utilized in a conventional CO-hydrogenation methanol synthesis unit to produce methanol as the final product. Storage options are integrated for all intermediate products. CO_2_ can be stored in liquid tanks, combined with liquefaction and regasification units^52^. Syngas storage is enabled by high-pressure tanks in combination with a syngas compressor. In an alternative process chain, PEM electrolysis is employed to generate H_2_ from the captured H_2_O. The produced hydrogen is then directly combined with CO_2_ in a CO₂-hydrogenation methanol synthesis unit to synthesize methanol. In the following, the individual model components are described.

The DAC model utilized for the main analysis is based on a one-dimensional process model of a solid sorbent or low-temperature DAC system, previously developed using Aspen Adsorption^22^. This process model simulates the cyclic temperature vacuum swing adsorption (TVSA) process, which consists of alternating adsorption and desorption phases and is widely employed in process simulations^53–55^. A solid sorbent DAC approach is chosen due to its advanced development status, with a reported technology readiness level of 9, making it the most mature DAC technology^56^. Furthermore, it is a DAC process with the potential to supply water rather than consume it^57^, a key attribute for our purposes. During the adsorption phase, ambient air is drawn through the adsorption column by a fan, allowing CO_2_ as well as H_2_O to be adsorbed. In the consecutive desorption phase, a combination of a partial vacuum and a temperature gradient serve as the driving force for desorption of the CO_2_/H_2_O mixture. The desorbed CO_2_ and H_2_O are then separated and can be utilized in downstream process steps. The DAC process requires power for operating the fans and vacuum pump, as well as heat for the desorption stage^17^. The required heat is supplied at a temperature of 90 °C^22^, which allows for integration of renewable energy by either utilizing a heat pump or an electric boiler. Additionally, waste heat from downstream processes could be integrated.

**Fig. 9:**
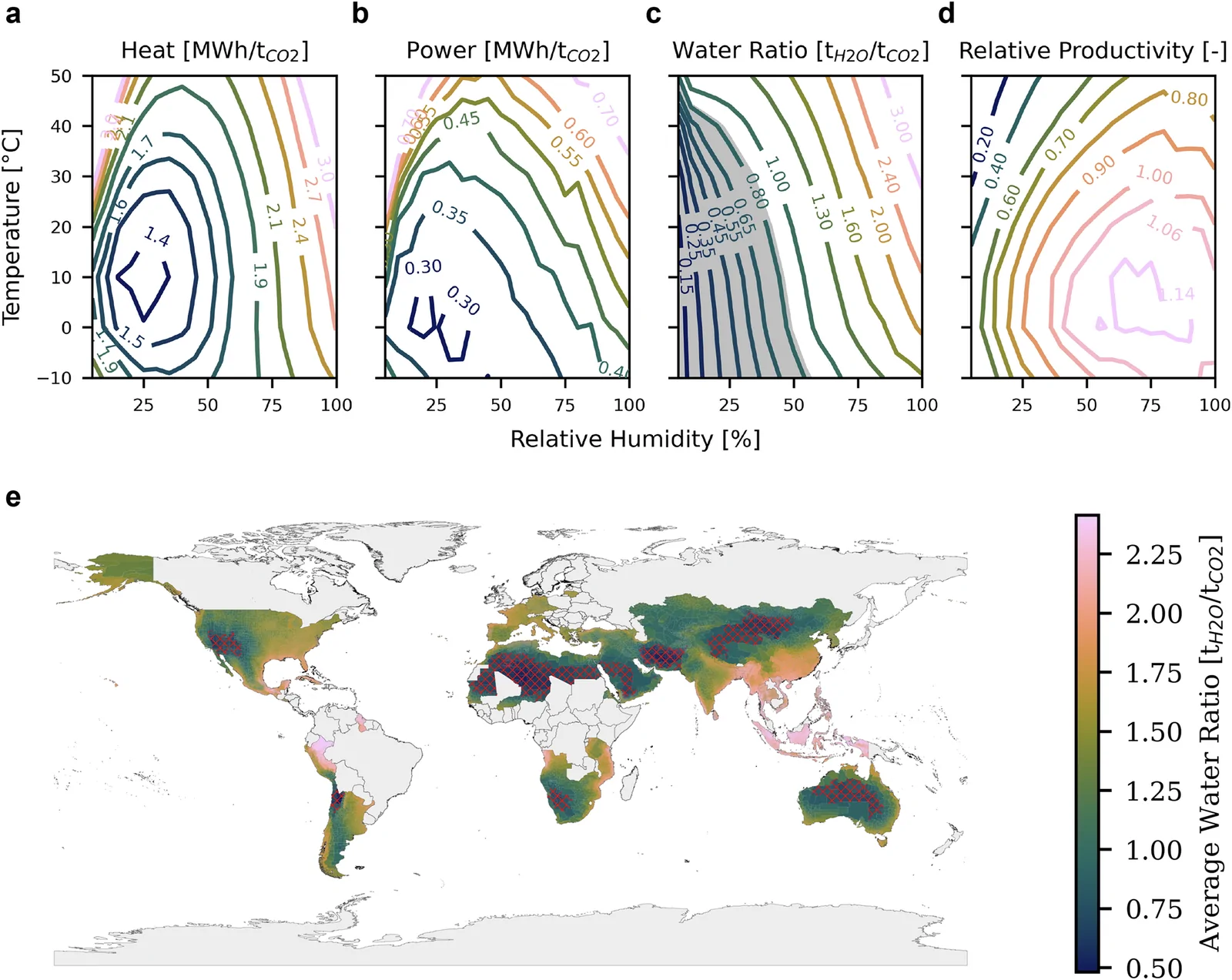
Weather-dependent key performance indicators from the utilized direct air capture (DAC) model^22^. **a**, **b** Specific heat and power demand. The data was scaled to incorporate future energy demand reductions. **c** Water supply ratio. The indicated gray area for water supply is the area in which no sufficient water desorption for methanol production is available, corresponding to a water ratio below 0.841 t_H2O_ t_CO2_^−1^. The water ratio refers to the desorbed water in the product stream. **d** Relative productivity. **e** Average water ratio in all investigated regions for the weather year 2018. Regions with insufficient average water co-adsorption are marked by red hatches. Country shapes from GADM^26^.

The DAC process model has been optimized for multiple environmental conditions, as ambient temperature and relative humidity significantly influence process productivity, energy requirements and the quantity of co-adsorbed H_2_O^22^. Based on the optimized process model, key performance indicators (KPIs) for the specific heat and power demand per produced CO_2_ as well as for the productivity and the quantity of desorbed water were derived for a variety of temperature and relative humidity combinations^22^. In this study, these weather-dependent KPIs are employed to model the influence of the prevailing weather conditions on DAC operation. However, the KPIs reflect the characteristics of a current DAC plant and do not account for future reductions in energy demand resulting from advancements in sorbent materials or process design. Since various studies predict a significant decrease in energy consumption^1,58,59^, a scaling factor has been applied to the weather-dependent KPIs to incorporate expected improvements. This factor is derived from a previous systematic literature review of solid sorbent DAC^17^. The review indicates a potential reduction in specific power demand to 64.6% of its current value and a decrease in specific heat demand to 64.7%, based on the median values of the present and long-term energy demands. While future reductions of the specific heat demand are potentially enabled by improved process design and heat integration^54^, water co-adsorption is anticipated to also decrease^60^. Thus, the same scaling factor is applied to the water co-adsorption KPI. Figure 9a–d presents the scaled weather-dependent KPIs used to model the DAC plant in this study.

The specific heat demand is influenced by both temperature and relative humidity, with higher values of either parameter generally leading to an increase in specific heat consumption (Fig. 9a). Particularly, higher humidity leads to higher water co-adsorption, which, in turn, results in a higher specific heat demand required to evaporate and desorb the water during the desorption cycle^22,61^. In contrast, the specific power demand is primarily affected by temperature (Fig. 9b). Higher temperatures result in greater specific power requirements, mainly driven by the lower working capacity of the sorbent, which necessitates increased air flow and therefore fan power^61^. As anticipated, increased relative humidity leads to higher water co-adsorption. A water-to-CO_2_ ratio below 0.841 t_H2O_ t_CO2_^−1^, indicates insufficient water capture for methanol synthesis, necessitating operational adjustments or buffer storage (Fig. 9c, e). Finally, relative productivity, which measures actual productivity under operating conditions compared to nominal productivity under design conditions, is highly dependent on prevailing weather conditions (Fig. 9d). This metric constrains the maximum operational capacity relative to the design capacity and is conceptually similar to the capacity factor of photovoltaic or wind power plants. In general, cold and humid conditions enhance relative productivity, as the working capacity of the sorbent is increased at low temperature and high humidity. Particularly, the presence of water leads to the formation of different species, e.g. water-stabilized carbamic acid, which increase the CO_2_ uptake capacity of amine-functionalized sorbents^32,44^. Additionally, an improved mass transfer under humid conditions is expected^44^.

To investigate the influence of DAC model choice, we utilize data provided by a second study^28,31^. The scaled, weather-dependent KPIs of this second DAC model are provided in Supplementary Fig. 11. Unlike most existing models, this alternative DAC model explicitly accounts for the effect of humidity on mass transfer, rather than assuming a constant mass transfer coefficient^28^. While we acknowledge the importance of additionally considering the temperature-dependency for mass transfer^45,62^, to the best of our knowledge no comprehensive study has yet quantified these effects while simultaneously reporting the required KPIs (energy demand, productivity, and water co-adsorption) across a broad range of ambient conditions.

The DAC model KPIs serve as an input for our assessment and are combined with hourly resolved weather data for each considered region to generate timeseries data for the DAC plant’s specific energy demand, relative productivity and water supply. The utilized ERA5 weather data^63^ is available at a spatial resolution of 0.25° × 0.25°. In cases where multiple grid points fall within the corresponding region, the data is averaged. If no grid point is within the current region, the four closest grid points are utilized and averaged. While the weather data used in this study is globally available at hourly resolution, potential effects of local microclimates, particularly in mountainous or coastal regions, cannot be captured due to the spatial resolution of the dataset. Such effects may, however, become relevant when determining the exact placement of DAC facilities within individual regions.

For future solid sorbent DAC plants, no significant cooling demand is anticipated, as effective heat integration is expected to be implemented^54^. For instance, heat from water desorption could be recovered through vapor recompression and integrated into the process^19^. Any residual cooling requirements could be met by a simple air flow through the contactor^64^. Consequently, the DAC plant model considers only power and heat demands, without accounting for external cooling requirements.

In the base assessment, SOEC technology is selected for electrolysis due to its ability to directly produce syngas and its lower water quality requirements, particularly compared to PEM electrolysis^34^. In addition, previous studies suggest that DAC products can be directly utilized in the SOEC^16^ and that water recovered from DAC is of comparable purity to deionized water^65^. The SOEC is modeled with a fixed energy efficiency of 80%, based on a previous systematic literature review where a total of 65 references for SOECs were examined^17^. The produced syngas is tailored to meet the required composition for downstream methanol synthesis and operates with a co-electrolysis ratio of 2.82 mol_H2_ mol_CO_^−1^. The necessary H_2_O and CO_2_ flows are determined based on this co-electrolysis ratio and the respective molar masses. While the SOEC operates at high temperature (700–800 °C) and typically incorporates heat integration between outlet and inlet streams^16,66^, additional cooling is necessary for compressors, water condensation and off-gas cooling. The cooling demand ${\dot{{{\rm{Q}}}}}_{{{\rm{cool}}}}$ is determined by applying an energy balance to the overall electrolysis system, taking into account the input power P, the resulting mass flows $\dot{{{\rm{m}}}}$ and enthalpies h as in Eq. (2) (for an overview of the system boundaries and assumptions see Supplementary Methods).

2 $${\dot{Q}}_{{cool}}=({{\rm{P}}}+{\dot{m}}_{H2O}\cdot {h}_{H2O}+{\dot{m}}_{{CO}2}\cdot {h}_{{CO}2}-{\dot{m}}_{{Syngas}}\cdot {h}_{{Syngas}}-{\dot{m}}_{O2}\cdot {h}_{O2})\cdot {\eta }_{{HX}}$$

For the SOEC, the specific cooling demand is calculated to be 48.5 kWh per MWh of electricity input, assuming a heat exchanger efficiency ${{{\rm{\eta }}}}_{{{\rm{HX}}}}$ of 85%^67,68^.

In the alternative process chain, PEM electrolysis is modeled with a system efficiency of 70%^33^. The resulting cooling demand for PEM electrolysis is 145 kWh per MWh electricity, which is significantly higher than the cooling demand of the SOEC. This difference is primarily attributed to the lower system efficiency of PEM electrolysis, which leads to increased waste heat generation.

The CO-hydrogenation unit is also parameterized based on a previous systematic literature review^17^. The plant requires 2.57 mol_H2_ mol_MeOH_^−1^, 0.91 mol_CO_ mol_MeOH_^−1^ and 0.34 mol_CO2_ mol_MeOH_^−1^, which is significantly more than the gross reaction would suggest. This increase is, in part, due to the necessity of a purge gas stream to prevent accumulation of impurities^69^. However, this purge gas is typically burned, and the released heat of 0.92 kWh kg_MeOH_^−1^ is considered for integration with upstream processes^17^. The associated carbon loss is accounted for through the optimization-based sizing of the DAC unit, ensuring that the overall system does not result in net CO_2_ emissions. During reaction, water formation occurs, and the crude methanol needs to be separated from the water in the distillation process^69^. This water, totaling 0.12 mol_H2O_ mol_MeOH_^−1^, can potentially be used as a feedstock for upstream processes. Finally, the methanol synthesis unit requires power for operation of the compressors and pumps, totaling 0.45 kWh kg_MeOH_^−1,^^17^_._ The methanol synthesis unit is modeled as fully flexible, not restricted by any operational constraints^70,71^. Generally, heat integration between hot and cold streams is performed in methanol synthesis units. However, excess cooling, often at lower temperatures, is usually required for the distillation process and possibly for compressors and product cooling^72,73^. As with the electrolysis process, the cooling demand of the methanol synthesis unit is calculated using an energy balance for the entire system. For the CO-hydrogenation process, a cooling demand of 0.875 kWh kg_MeOH_^−1^ is derived. The underlying assumptions and system boundaries are elucidated in the Supplementary Methods.

For the alternative process chain, a CO_2_-hydrogenation unit is modeled with a feedstock consumption of 3.2 mol_H2_ mol_MeOH_^−1^ and 1.06 mol_CO2_ mol_MeOH_^−1^, along with a water production of 1.03 mol_H2O_ mol_MeOH_^−1^, based on an evaluation of various literature sources (see Supplementary Methods). The power consumption totals 0.72 kWh kg_MeOH_^−1^, which is higher than that of the CO-hydrogenation unit, primarily due to the increased feedstock flow, which requires compression. The heat released from burning the purge gas is 0.49 kWh kg_MeOH_^−1^, lower than in the case of CO-hydrogenation due to the lower over-stochiometric feedstock consumption. The required cooling demand totals 1.47 kWh kg_MeOH_^−1^, significantly higher than that of the CO-hydrogenation process. While CO_2_-hydrogenation is less exothermal, the increased cooling demand is attributed to the greater cooling requirements for compressors and the higher cooling needs in the purification system, as the crude methanol contains a significantly higher water content that must be separated.

### Air cooling model

For water-conscious methanol production, cooling is as crucial as supply of the necessary water feedstock. Most techno-economic assessments do not consider cooling^6^ or assume unrestricted availability of cooling water^35,74,75^. However, such assumptions are not valid in arid regions and could become increasingly problematic globally, as rising occurrences of droughts and extreme temperature events are reducing water availability and potentially leading to plant shutdowns, as has been observed in the power industry^15^. Therefore, a water-conscious cooling approach is essential.

While cooling systems for thermal power plants have received significant attention in the past^24,76–78^, few assessments have focused on cooling systems for renewable energy plants^23,79^. There are various cooling options available, such as once-through or evaporative cooling systems, but these require an external water supply to dissipate heat. The only system that does not depend on an external water source is the dry or air cooling system^23^. This approach employs a closed-loop cooling fluid circuit in which heat is transferred from the plant to a heat exchanger and subsequently dissipated to the environment by an air flow (Supplementary Fig. 3).

Typically, A-frames with a finned surface are used for the cooling system^76^, as they offer a high surface area and enable effective heat transfer to the air. Fans are employed to blow air through the A-frame and increase heat transfer. The required air flow can be calculated as in Eq. (3) using an energy balance and depends on the cooling demand ${\dot{Q}}_{{cool}}$, the heat capacity of the air ${c}_{p,{air}}$, the temperature of the heat ${T}_{{cool}}$ with a heat transfer temperature difference ($\Delta T=\,5{{\rm{K}}}$), and the ambient air temperature ${T}_{{air}}$ (see Supplementary Methods for further assumptions).

3 $${\dot{m}}_{{air}}=\frac{{\dot{Q}}_{{cool}}}{{c}_{p,{air}}\cdot \left({T}_{{cool}}-\Delta T-{T}_{{air}}\right)}$$

4 $${P}_{{Fan}}=\frac{{\dot{m}}_{{air}}\cdot \Delta p}{{\rho }_{{air}}\cdot {{{\rm{\eta }}}}_{{Fan}}\,}$$

The fan power P_Fan_ is subsequently calculated by Eq. (4) utilizing the air density ρ_air_, the fan efficiency ${{{\rm{\eta }}}}_{{Fan}}$, typically 70%^23^, and the air side pressure drop ∆p. The latter is depending on the exact geometry of the air cooler and influenced by factors such as the flow velocity and air temperature^77^. As we do not intend to design an exact air cooling system, we employ a constant pressure drop of 261 Pa from the optimized air cooler design of a previous study^77^. However, it should be noted that the pressure drop can vary considerably, ranging from ~100 to 500 Pa^77^, strongly effecting the power demand.

The cooling fluid is circulated by a pump, which must overcome the pressure drop within the cooling circuit as well as the height of the A-frame. The power demand for the pump can be calculated using Eqs. (3) and (4) by substituting the air properties with those of the cooling fluid, i.e. water. The pressure drop on the water side is assumed to be 200 kPa^24^, considering a pump efficiency of 70%^23^.

The required area of the A-frame can be calculated based on the heat transfer in heat exchangers by considering the heat transfer coefficient ${\alpha }_{{air}}$ of 1135 W m^−2^ K^−1^ for the air-cooled system^24^ and the cooling load (see Eq. (5)).

5 $$A=\frac{{\dot{Q}}_{{cool}}}{{\alpha }_{{air}}\cdot \Delta {{\rm{T}}}}$$

The cost of the air cooling system depends on the size of the A-frame as well as on the necessary fan and pump capacities. The capex are derived by using Supplementary Eqs. (1)–(3) from a previous study^79^ (see Supplementary Methods).

As shown in Eqs. (3) and (4), the power demand of the air cooling system depends on the ambient air temperature. Therefore, the air cooling model is combined with hourly resolved temperature data for each considered region to generate timeseries data of the power demand. The effect of the air temperature on power demand is further illustrated in Supplementary Fig. 6.

The air cooling system can only provide cooling when the ambient air temperature is lower than the temperature of the heat load minus the necessary temperature difference for heat transfer. During periods of excessively high ambient temperatures, the air cooling system must shut off. Moreover, the system’s operation is constrained by the ambient air temperature, as higher temperatures reduce the amount of heat that can be transferred to the air. This interaction is accounted for by integrating the model with hourly resolved weather data for each region. The influence of air temperature on the air cooling system’s operation is also detailed in Supplementary Fig. 5.

### Renewable energy supply system

To power the process chain, a renewable energy supply system is utilized. This system provides power and heat to the process and handles the generated waste heat by providing cooling utility (Fig. 1b). Generally, the process chain requires power for operation of the DAC plant, the SOEC, the methanol synthesis unit as well as for operation of compressors and liquefaction units (Fig. 1a). Heat is primarily required by the DAC plant for the desorption process. The temperature needed for this process is 90 °C; however, to account for the heat transfer driving force, the energy supply system provides heat at 100 °C. This heat can either be supplied directly from power by an electric boiler or an air source heat pump can be utilized to achieve higher efficiency. The coefficient of performance (COP) of the air source heat pump is calculated by Eq. (6) with the hot side temperature ${T}_{H}$ = 100 °C and the cold side temperature$\,{T}_{C}$ being equal to the ambient air temperature, considering a 2^nd^ law efficiency of 50%^27^.

6 $${{\rm{COP}}}=\frac{{T}_{H}}{{T}_{H}-{T}_{C}}\cdot {\eta }_{2{nd}}$$

By combining Eq. (6) with the hourly resolved air temperature data in each considered region, the COP of the air source heat pump can be determined for each hour and region. The heat generated from burning the purge gas of the methanol synthesis unit can be directly integrated for the desorption phase of the DAC unit or it could be stored together with other generated heat in a heat storage. The waste heat generated by the SOEC and methanol synthesis unit is assumed to be available at 40 °C. This assumption is generally conservative, as the actual temperature levels of the waste heat are ~50 °C for the SOEC^80^ and between 30 and 40 °C for the methanol synthesis unit^72,73^. However, differentiating between the two waste heat source would add complexity to the model with only slight improvements in accuracy. The waste heat can either be utilized in a heat pump with a constant COP = 3.11 (see Eq. (6)) to generate heat at 100 °C or it has to be cooled by the air cooling system. As described above, the air cooling system’s power demand and maximal operation depend on the ambient air temperature and cooling at an air temperature above 35 °C is not possible. In that case, all heat needs to be integrated or stored for later utilization.

The power supply by onshore wind and horizontal single-axis tracking open-field PV is modeled based on spatially-explicit analyses carried out in corporation with the International Energy Agency for their Global Hydrogen Review 2024^14,21^. For each considered region, the installable capacity is limited by the maximum potential derived from land eligibility analysis and the operational output in each hour is limited by hourly resolved supply profiles based on simulations with historic weather data^21^. For all analyses, the weather year 2018 was chosen as it is a representative weather year^81^ and in line with the utilized renewable energy potentials and supply profiles^14^. The resulting full load hours for each region as well as the maximum installable potential are detailed in Supplementary Figs. 9, 10.

## Supplementary information

Supplementary Information

Transparent Peer Review file

## Data Availability

All optimization results and source data for the figures are provided with this paper under the following: 10.6084/m9.figshare.30664253.

## Code availability

The frameworks required to reproduce this study are available open source. Weather-dependent simulations of direct air capture plants and air-cooling systems were performed using ETHOS.RESKit^82^ (https://github.com/FZJ-IEK3-VSA/RESKit), while regional energy system models were implemented using ETHOS.FINE^83^ (https://github.com/FZJ-IEK3-VSA/FINE). All model parameters required to reproduce the analyses are provided in the manuscript and Supplementary Information.
