# Supplementary material for: Direct air capture enables sustainable methanol production in water-scarce regions
Source: Nat Commun. 2026 Aug 24;17:8495. doi: 10.1038/s41467-026-76865-x (PMC13503827; doi:10.1038/s41467-026-76865-x)
Supplement: Supplementary file 1 [file 41467_2026_76865_MOESM1_ESM.pdf]

Supplementary Information - Direct air capture enables sustainable methanol production in water-scarce regions

Henrik Wenzel[1,2,*], Thomas Schöb[1], David S. Sholl[3,4], Jochen Linßen[1], Detlef Stolten[2] and Jann M. Weinand[1]

[1] Forschungszentrum Jülich GmbH, Institute of Climate and Energy Systems – Jülich Systems Analysis (ICE-2), 52425 Jülich, Germany

[2] RWTH Aachen University, Chair for Fuel Cells, Faculty of Mechanical Engineering, 52062 Aachen, Germany

[3] University of Tennessee-Oak Ridge Innovation Institute, Oak Ridge National Laboratory, Oak Ridge, TN 37830, USA

[4] Present Address: Dept. of Chemical & Biomolecular Engineering, Rice University, Houston, TX 77005, USA

* corresponding author: h.wenzel@fz-juelich.de

Supplementary Items

Supplementary Methods **3**
Calculation of Waste Heat Generation 3
Supplementary Figure 1 3
CO_2-hydrogenation 3
Supplementary Table 1 4
Supplementary Figure 2 4
Air Cooling Model 4
Supplementary Figure 3 4
Supplementary Figure 4 5
Supplementary Figure 5 5
Supplementary Figure 6 6
Techno-economic Assumptions 6
Supplementary Table 2 6
Supplementary Note 1 .... **7**
Supplementary Figure 7 8
Supplementary Figure 8 9
Supplementary Figure 9 9
Supplementary Figure 10 10
Supplementary Figure 11 11
Supplementary Note 2 .... **11**
Global Analyses 11
Supplementary Figure 12 11
Supplementary Figure 13 12
Supplementary Figure 14 13
Supplementary Figure 15 13
Supplementary Figure 16 14
Operation in Selected Regions 14
Supplementary Figure 17 15
Supplementary Figure 18 16
Supplementary References **17**

Supplementary Methods

Calculation of Waste Heat Generation

For calculating the waste heat generation by the electrolysis system, the first law is applied to the total system (see Supplementary Figure 1). By adding all power demands, the total generated waste heat can be calculated as shown in the main document. Sweep gas air flow is not included, as the inlet and outlet mass and enthalpy stay the same. It is assumed that the generated oxygen can be purged at the operating temperature of the SOEC (800 °C) and does not need to be cooled down. The inlet as well as the outlet are assumed to be at 25 °C. All enthalpy data is taken from substance data tables[1]. The heat exchanger efficiency is included to account for heat losses to the environment.

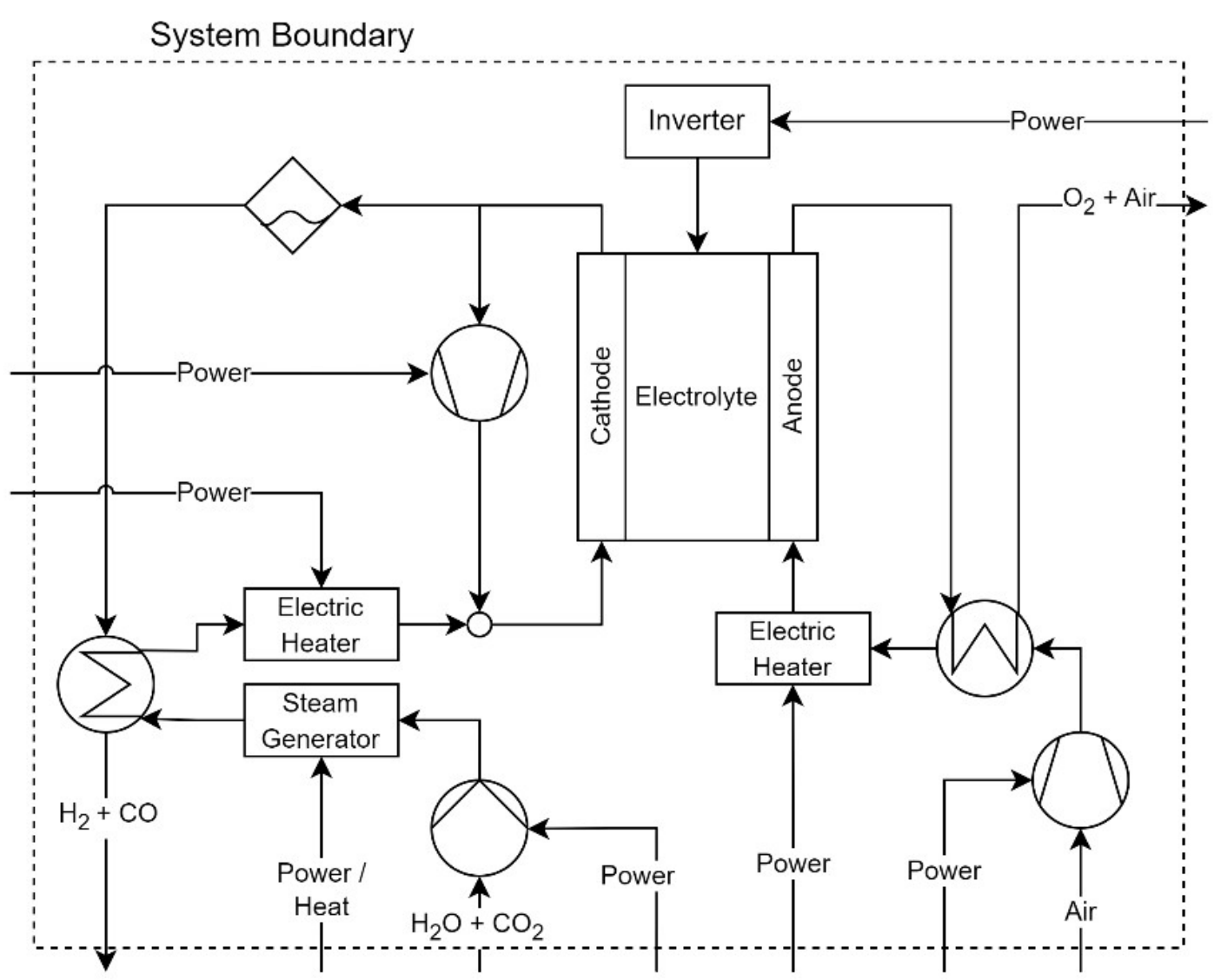

Supplementary Figure 1: System boundary of the considered solid oxide electrolysis (SOEC) system. The air flow is depicted but not included in the calculation, as inlet and outlet mass and enthalpy are the same. Waste heat generation can occur in all components but is not depicted. Process symbols adapted from the draw.io Process Engineering library.

The calculation of the methanol synthesis unit's waste heat generation is the same as for the electrolysis, considering all input and output mass flows and corresponding enthalpies. For methanol synthesis all input and output flows are assumed to be at 25 °C, including the purge gas stream.

CO_2-hydrogenation

To parameterize the direct CO_2-hydrogenation unit, a literature review was conducted in the same order as for CO-hydrogenation in our previous systematic literature review[2]. A total of 37 sources[3–39] were evaluated and the data was harmonized to account for differences in the corresponding sources[2]. The literature review was conducted by searching web of science with the search strings and settings as detailed in Supplementary Table 1.

Supplementary Table 1: Search strings and filters for the conducted systematic literature review on CO_2-hydrogenation parameters.

techno-economic AND (methanol synthesis OR methanol reactor) AND (CO2 OR green OR CO2 hydrogenation OR carbon dioxide)	All fields, 2020-2024, Concluded at 05.11.2024	No. results 209, Included in review 37

All abstracts were scanned for relevant information. In case the abstract seemed promising, the article was read and, if relevant information was found, it was added to the review.

The resulting specific capex and specific consumption are given in Supplementary Figure 2. All data employed in this study is based on the median values from the literature review and detailed in section Techno-economic Assumptions.

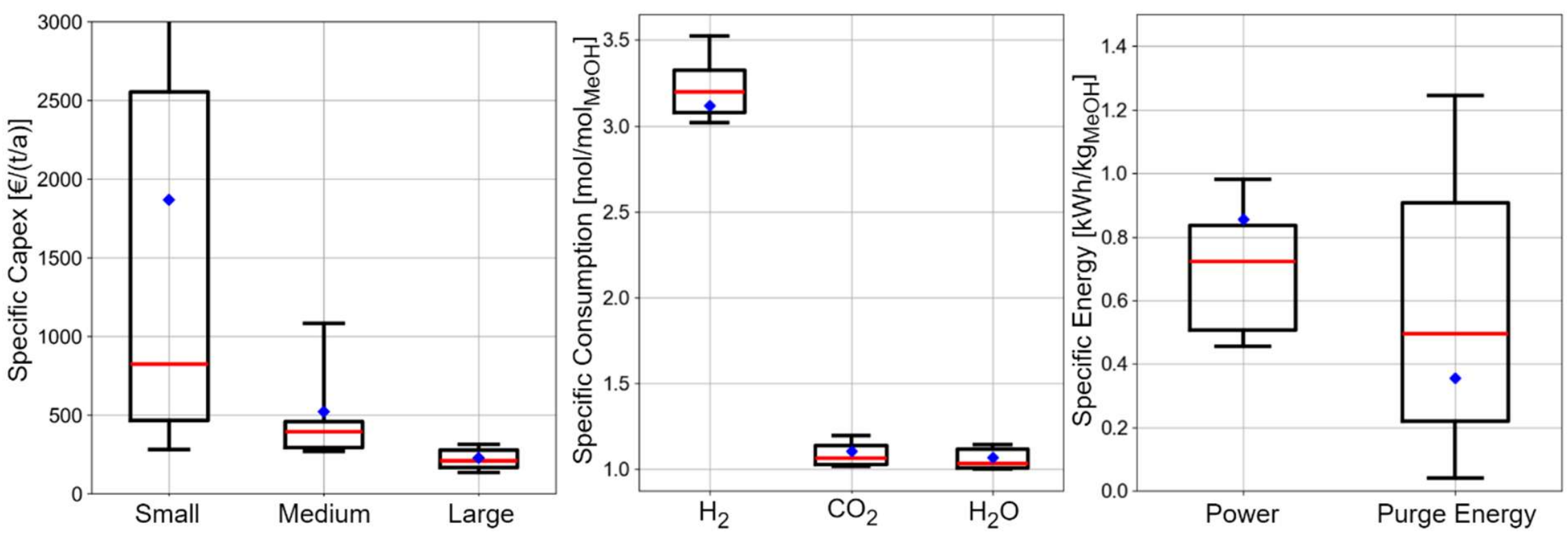

Supplementary Figure 2: Results of the conducted literature review for CO_2-hydrogenation after harmonization based on 37 sources[3–39]. The capex are given for different system sizes, where small corresponds to less than 100 kt a^{-1}, medium is 100-500 kt a^{-1} and large is >500 kt a^{-1}. The red line indicates the median value utilized for the assessment, while the blue diamond corresponds to the average value.

Air Cooling Model

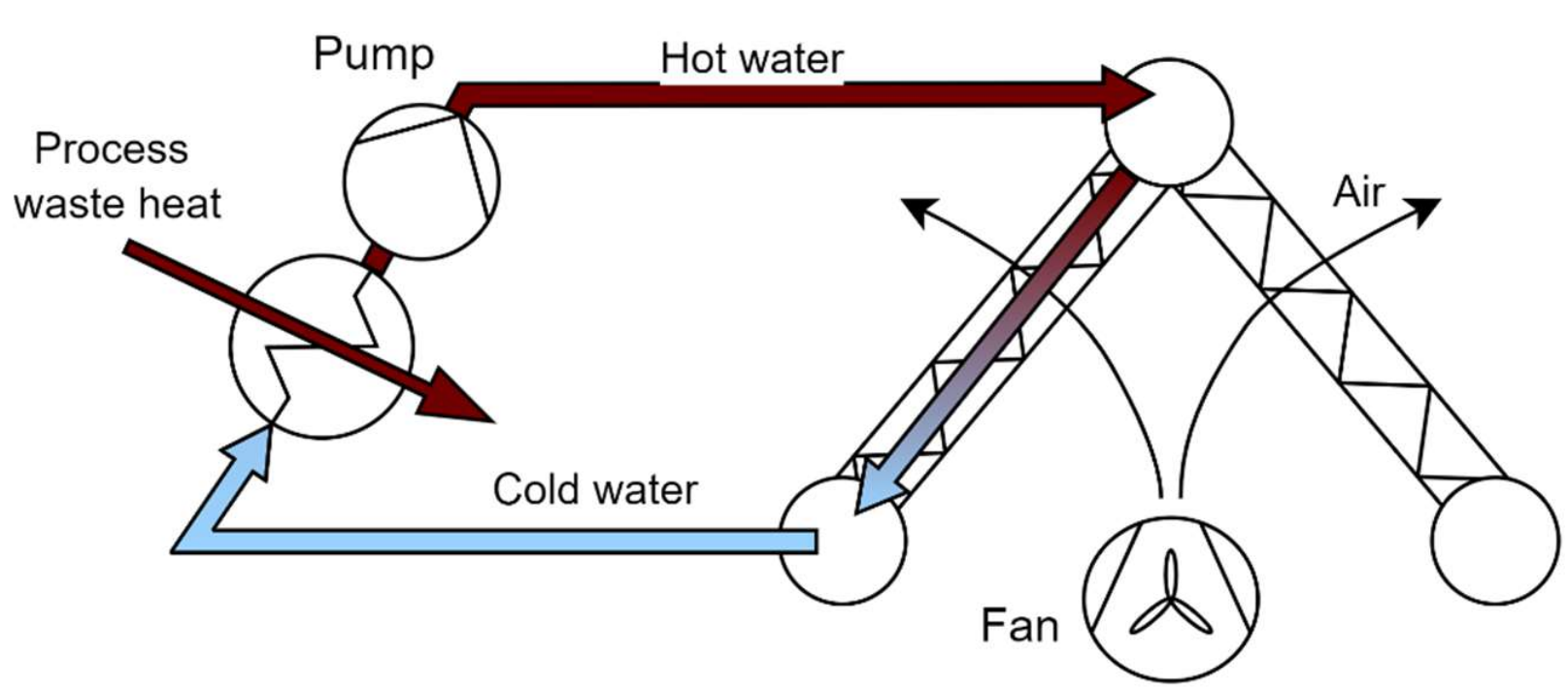

Supplementary Figure 3: Schematic representation of the air cooling system. The cooling water circuit is closed loop and transfers the heat from the process to the A-frame for cooling. A pump is used for water circulation and fans are utilized for generating the necessary air flow. Process symbols adapted from the draw.io Process Engineering library.

The cost of the air cooling system is calculated based on supplementary equations (1)-(3) from a previous study[40]:

$$PEC_{Pump} = 3540 \cdot \mathrm{P[kW]}^{0.71} \quad (1)$$

$$PEC_{Fan} = 12300 \cdot (\frac{P[kW]}{50})^{0.76} \quad (2)$$

$$PEC_{A-frame} = 156000 \cdot (\frac{A[m^2]}{200})^{0.89} \quad (3)$$

To derive the capex based on the purchase equipment cost (PEC) a Lang-factor of 2.8 is applied[40]. The capex is dependent on the needed installed power of fans and pumps and the size of the A-frame. While the A-frame size only depends on the cooling load, the needed power also depends on the air temperature. For simplification, the capex of the air cooling system have been calculated for a cooling load of 300 MW_{th} and a design ambient air temperature of 20 °C, which results in specific capex of about 200 € kW_{th}^{-1}. However, it should be noted that the specific capex heavily depends on the system size:

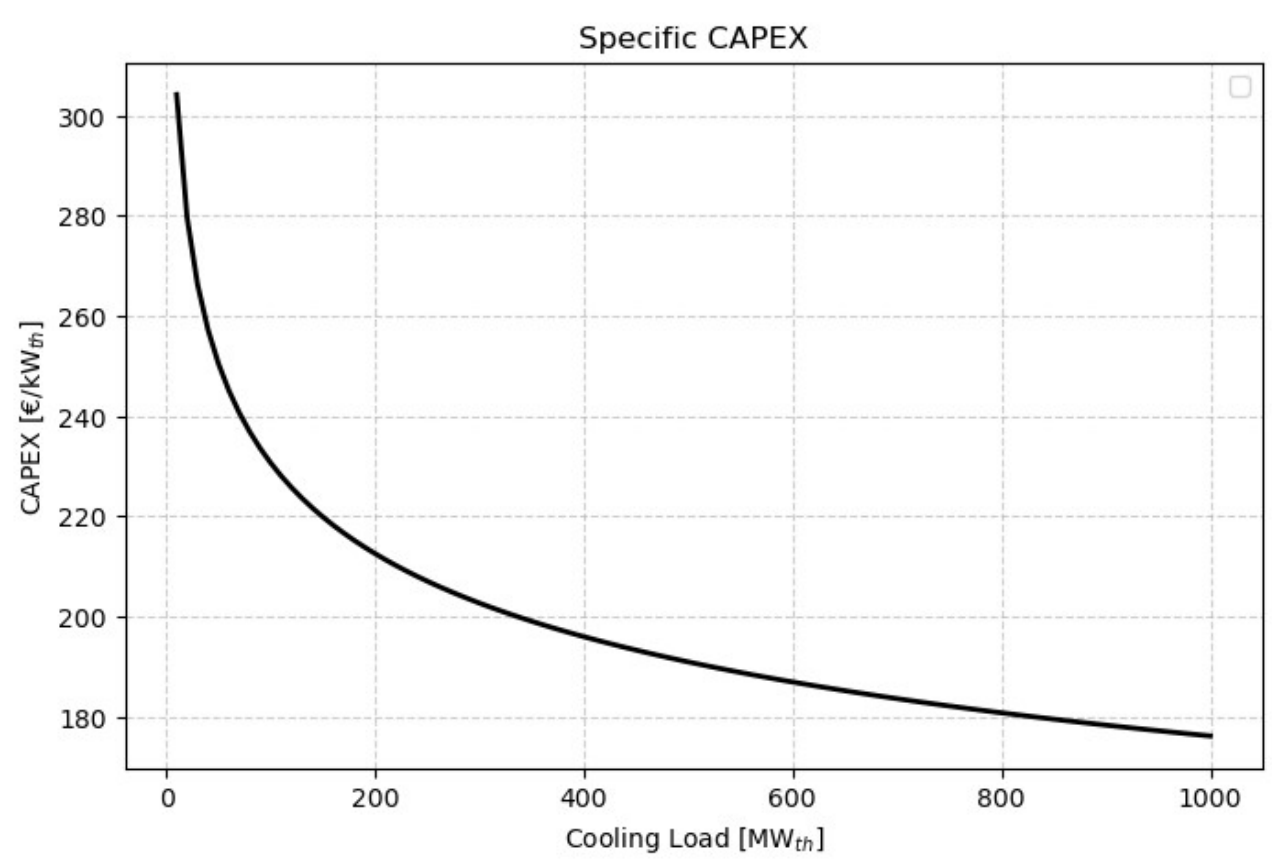

Supplementary Figure 4: Specific capex of the air cooling system depending on the ambient air temperature.

At higher air temperature higher specific capex results, as more fan power needs to be installed. This relationship is incorporated into the model by limiting the maximum operation rate at ambient air temperatures differing from the design temperature:

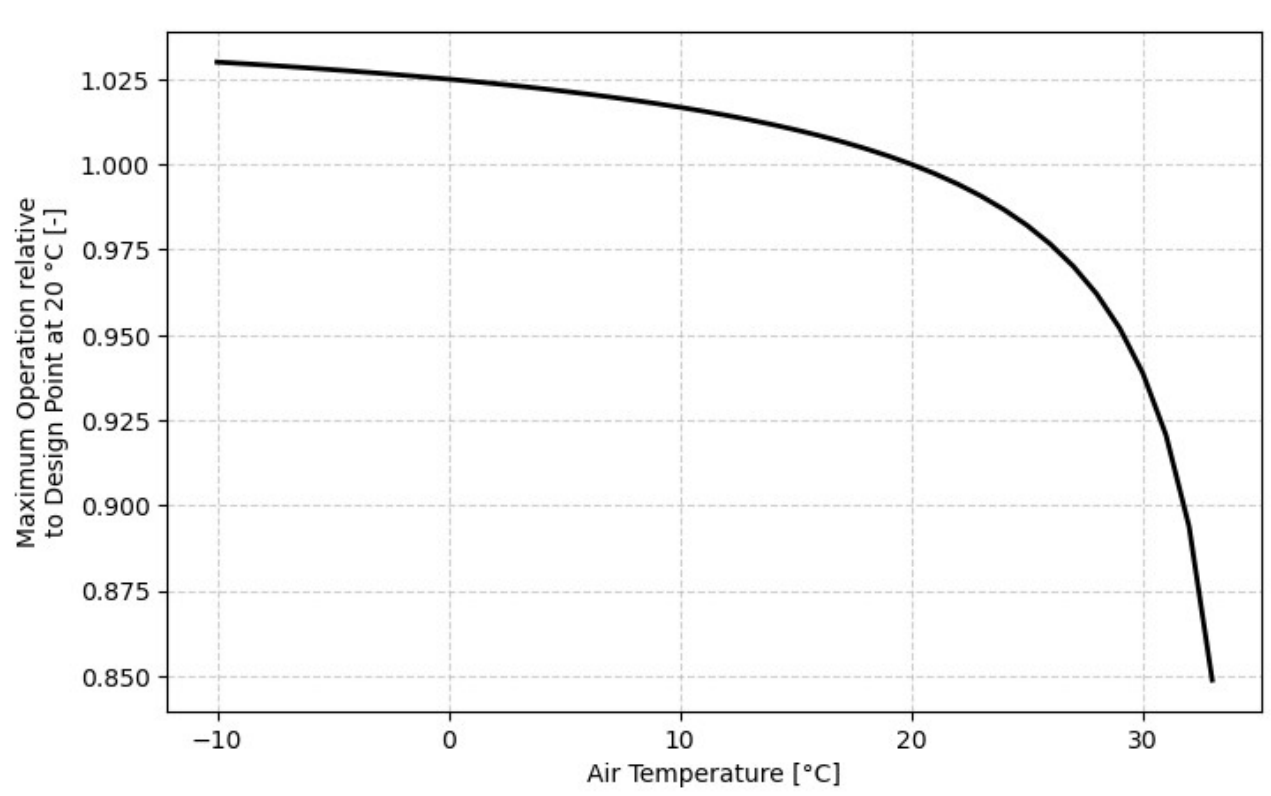

Supplementary Figure 5: Maximum possible operation of the air cooling system relative to the installed capacity.

The influence of different ambient temperatures on the specific power demand of the air cooling system is illustrated in Supplementary Figure 6.

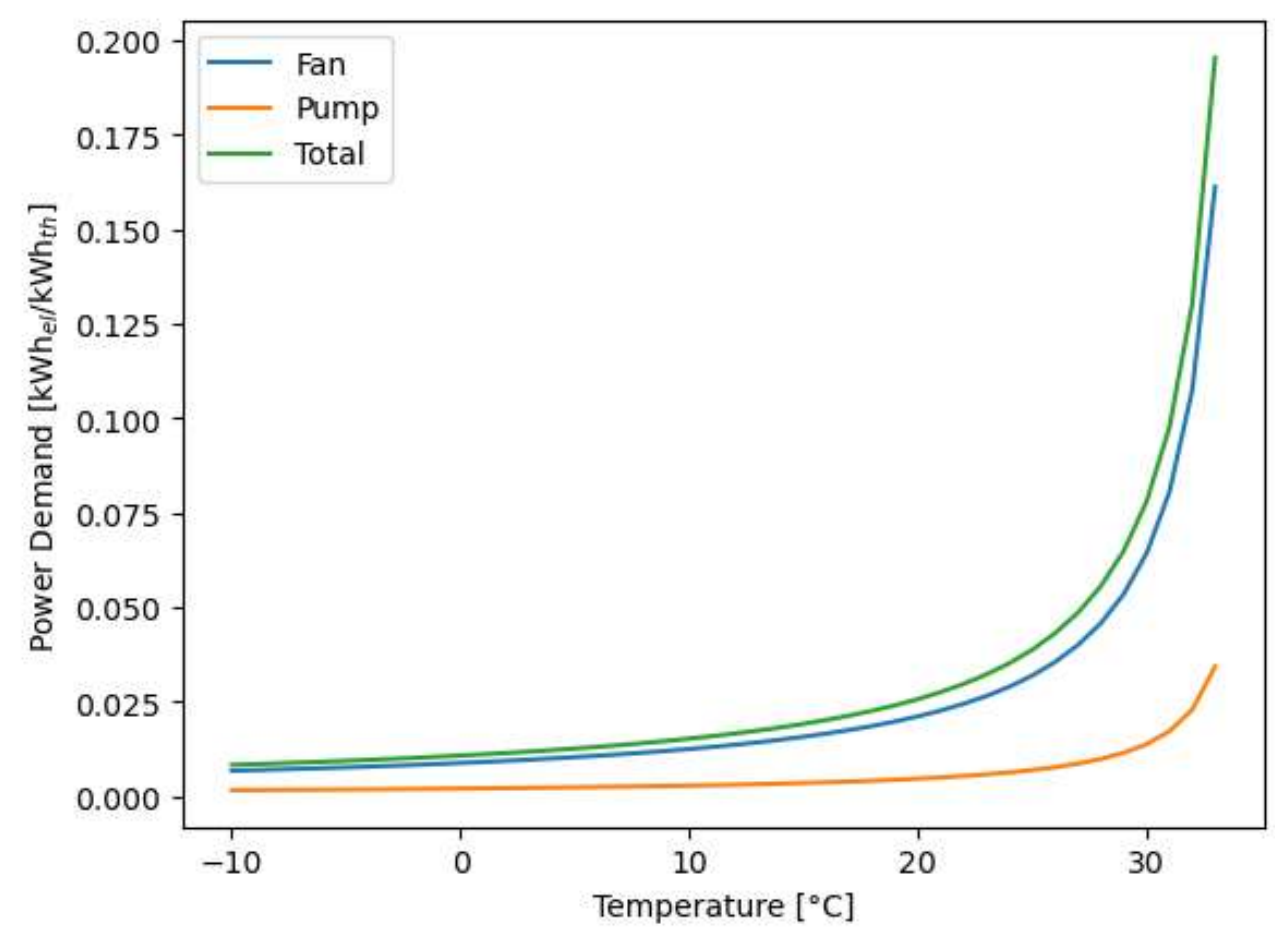

Supplementary Figure 6: Specific power demand of the air cooling system dependent on the air temperature.

Techno-economic Assumptions

All techno-economic parameters have been converted to € and inflation adjusted as stated in the main document. All values are based on refs[2,41–54] and shown in Supplementary Table 2.

Supplementary Table 2: Techno-economic parameters based on the indicated literature sources. The data was harmonized, i.e. inflation adjusted and converted to €.

Technology	Capex	Fix Opex (% of Capex)	Efficiency / Energy	Economic Lifetime	Remarks
DAC	316 € t^{-1} a[2]	4%[2]	See main document	20 a[2]	
SOEC	776 € kW_{el}^{-1}[2]	4%[2]	80% LHV[2]	20 a[2]	
CO-hydrogenation	177 € t^{-1} a[2]	10%[2]	See main document	20 a[2]	
PEM	553 € kW_{el}^{-1}[41]	2%[42]	70% LHV[41]	20 a[42]	
CO_2-hydrogenation	210 € t^{-1} a	3.5%	See main document	20 a	Based on review, see CO2-hydrogenation
Horizontal single-axis tracking OFPV	611 € kW^{-1}[43]	2%[43]		30 a[43]	
Onshore wind	893 € kW^{-1}[43]	2.5%[43]		30 a[43]	
Heat pump	665 € kW_{th}^{-1}[44]	2%[44]	See main document	25 a[44]	
Electric boiler	148 € kW^{-1}[44]	0.4%[44]	99%[44]	20 a[44]	

Air cooling	200 € kW_{th}^{-1}	2%[45]		25 a[45]	See Air Cooling Model
CO_2 liquefaction	21 € t^{-1} a [46]	10%[46]	83.1 kWh t^{-1} [46]	25 a[47]	
CO_2 gasification	1.1 € t^{-1} a [46]		75.7 kWh t^{-1}	25 a[47]	Energy demand calculated by 1st law. Assumed liquid storage at 30 bar & -6 °C and heat up to 25 °C.
Syngas compression	460 € kW^{-1} [48]	5.2%[49]	1.32 MWh t^{-1}	20 a[48]	Energy demand calculated based on isentropic compression with an efficiency of 85%[50] and a compression ratio of 300
Battery	170 € kWh^{-1} [51]	2.5%[51]	Charge- / Discharge: 96%/96%[51] Self-discharge: 1.3% h^{-1}	15 a[51]	Self-discharge calculated based on round trip efficiency of 85% for 6h batteries[43]
Heat storage	1 € kWh^{-1} [52]	2%[51,52]	98%/98%[51] 0.01% h^{-1} [51]	20 a[51]	
Water storage	27 € t^{-1} [53]			30 a	No upper limit is imposed on the retention time, and evaporation losses are not considered, assuming closed storage tanks.
CO_2 storage	1100 € t^{-1} [46]	5%[46]		25 a[47]	Liquid storage
Syngas storage	60 € kg^{-1} [54]	3.65%[54]		10 a	300 bar pressure tanks

Supplementary Note 1

The yearly average values of all region-specific input data are given in this section. Since the model employs an hourly resolution, the yearly average does only give a general perspective about the input data. However, as described in the main document, significant correlations between the average data and different metrics have been observed.

Supplementary Figure 7 shows the average yearly temperature and relative humidity for all analyzed regions based on ERA5 data[55] and visualized using country shapes from GADM[56]. Yearly average temperatures as low as -10 °C in parts of China and up to more than 30 °C in parts of Africa, India and Australia can be observed. The yearly average relative humidity varies from below 20% in Northern Africa to close to 90% in Indonesia and Peru.

Supplementary Figure 9 shows the yearly full load hors (FLH) of OFPV systems and onshore wind plants across the globe. While OFPV FLH vary between about 1000 h a^{-1} to approximately 3200 h a^{-1}, an even greater variation is observed for wind plants with FLH less than 1000 h a^{-}

[1] to more than 5000 h a^{-1} in Patagonia. It should be noted that for each region all individual placements have been aggregated to receive FLH of the total installable capacity. Especially for wind turbines, individual placements can experience significantly higher FLH. To account for the differences between individual locations within one region, 10 different supply profiles for wind plants and 3 different supply profiles for OFPV systems have been included for each considered region. The supply profiles have been derived from simulation with historic weather data[57,58].

Supplementary Figure 10 shows the maximal installable capacity for onshore wind turbines and horizontal single axis tracking (HSAT) OFPV systems in each region. These potentials have been derived in cooperation with the International Energy Agency for their Global Hydrogen Review 2024[58] and are based on land eligibility analysis performed for each region[57].

Supplementary Figure 11 shows the weather-dependent KPIs of the alternative DAC model[59].

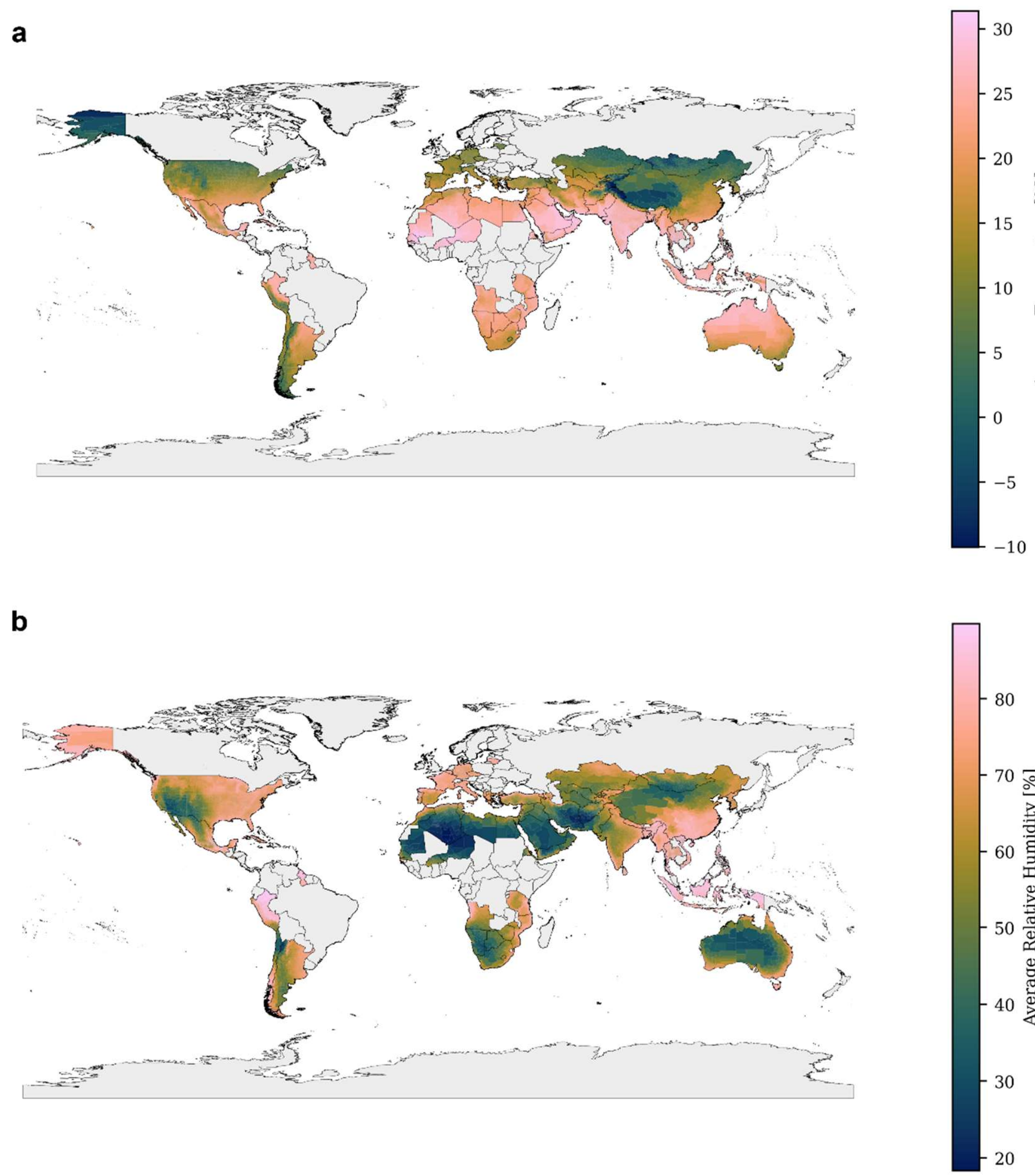

Supplementary Figure 7: Average temperature (a) and relative humidity (b) in all considered regions for the considered weather year 2018[55]. Country shapes from GADM[56].

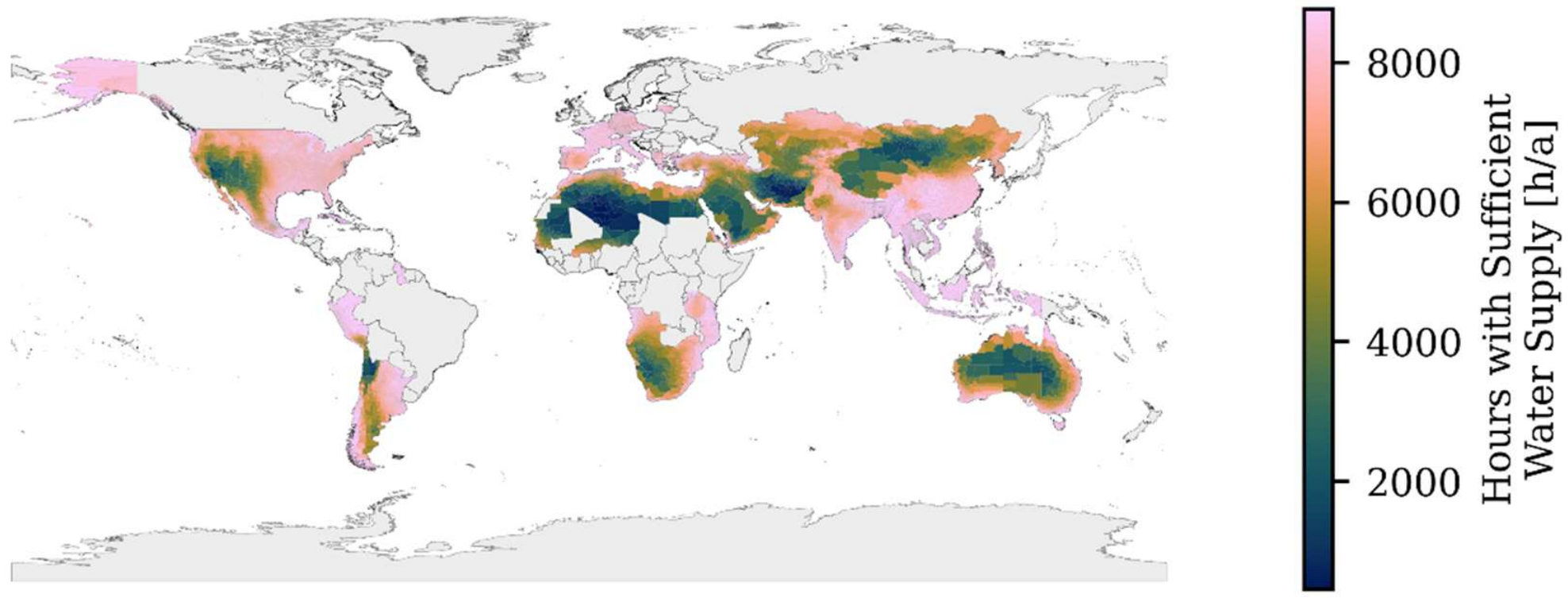

Supplementary Figure 8: Number of hours per year with sufficient (>0.841 t_{H2O} t_{CO2}^{-1}) water supply by the DAC plant. The region with the lowest number of hours (456 h a^{-1}) can be found in southern Algeria. Country shapes from GADM[56].

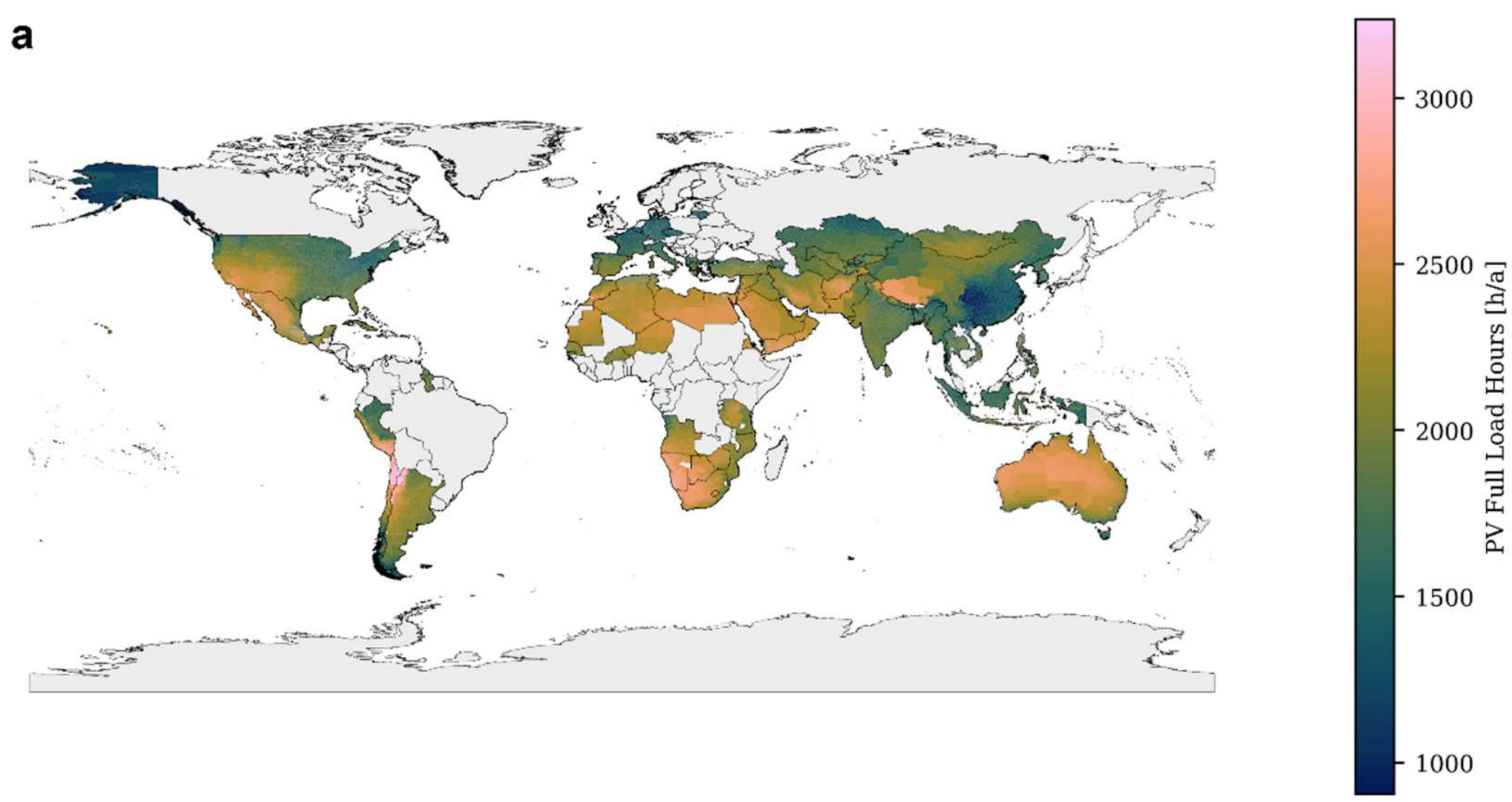

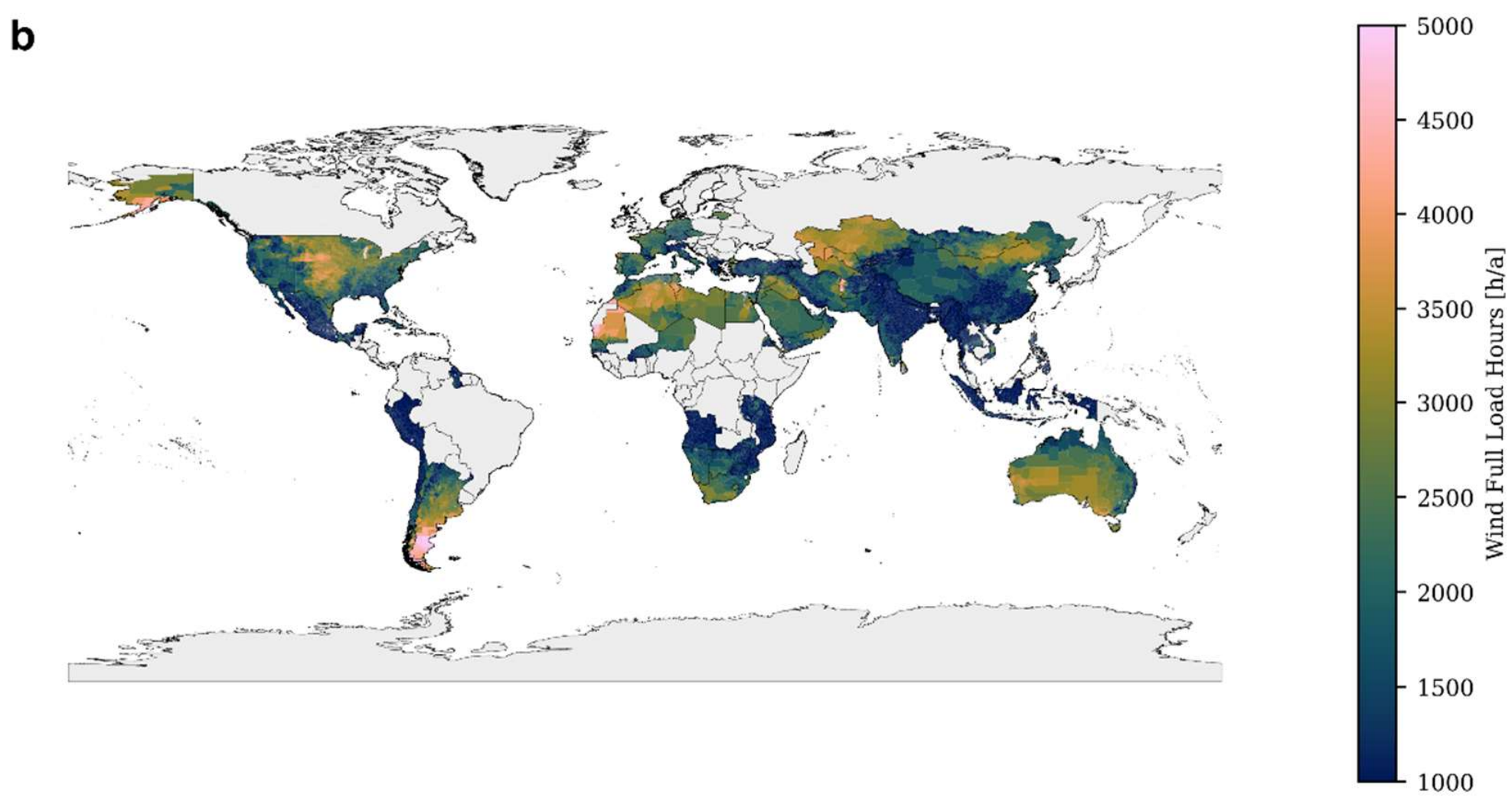

Supplementary Figure 9: Full load hours (FLH) of open-field photovoltaic (OFPV) systems (a) and wind turbines (b) in the analyzed regions. All data was clustered for each region, therefore the depicted data is

an average for all possible wind turbines / OFPV plants in the corresponding region and plants reaching higher FLH exist in each of the regions[57,58]. Country shapes from GADM[56].

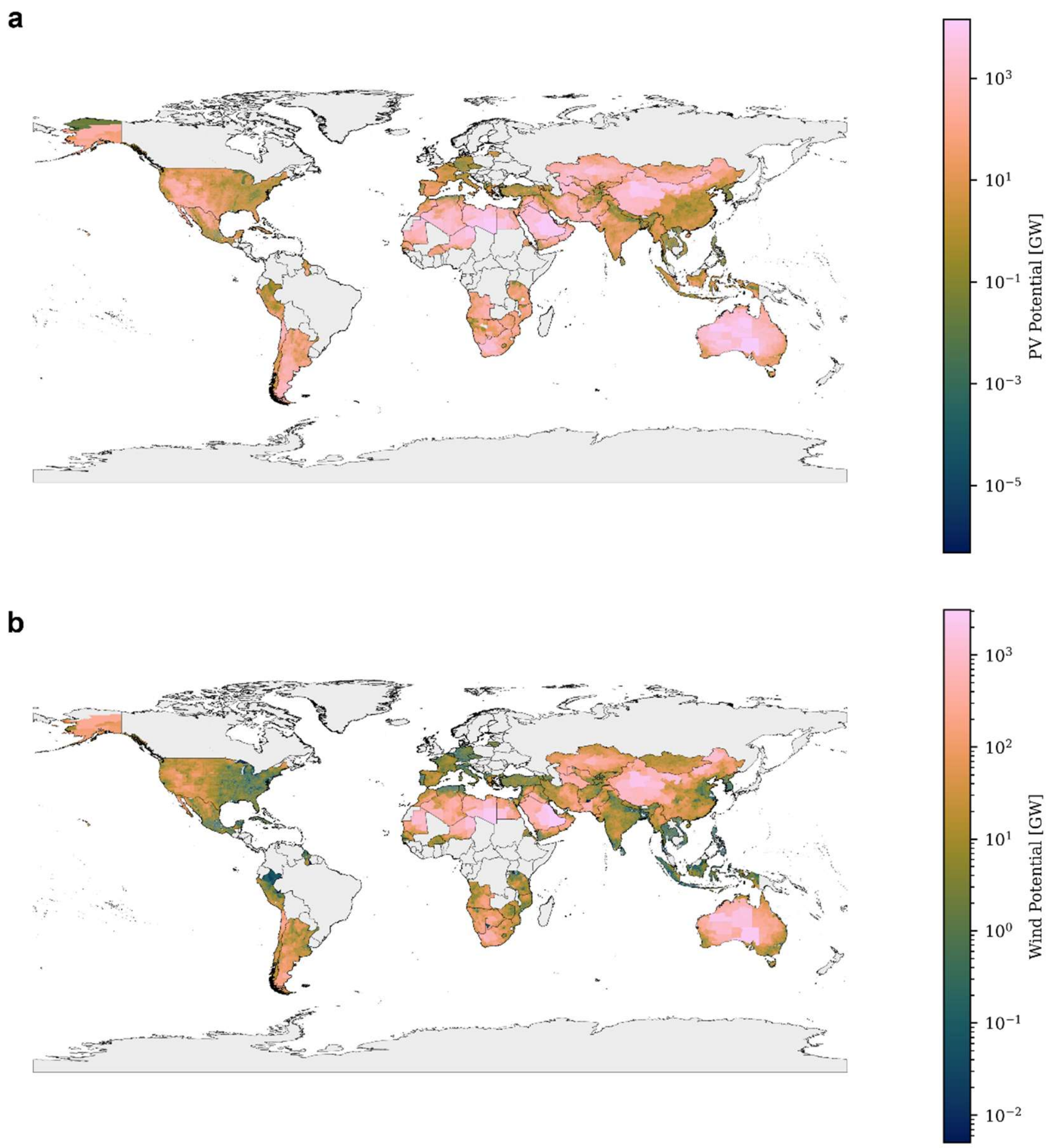

Supplementary Figure 10: Aggregated potential in all considered regions[57,58]. a. Open-field photovoltaic (OFPV) potential. b. Onshore wind potential. Country shapes from GADM[56].

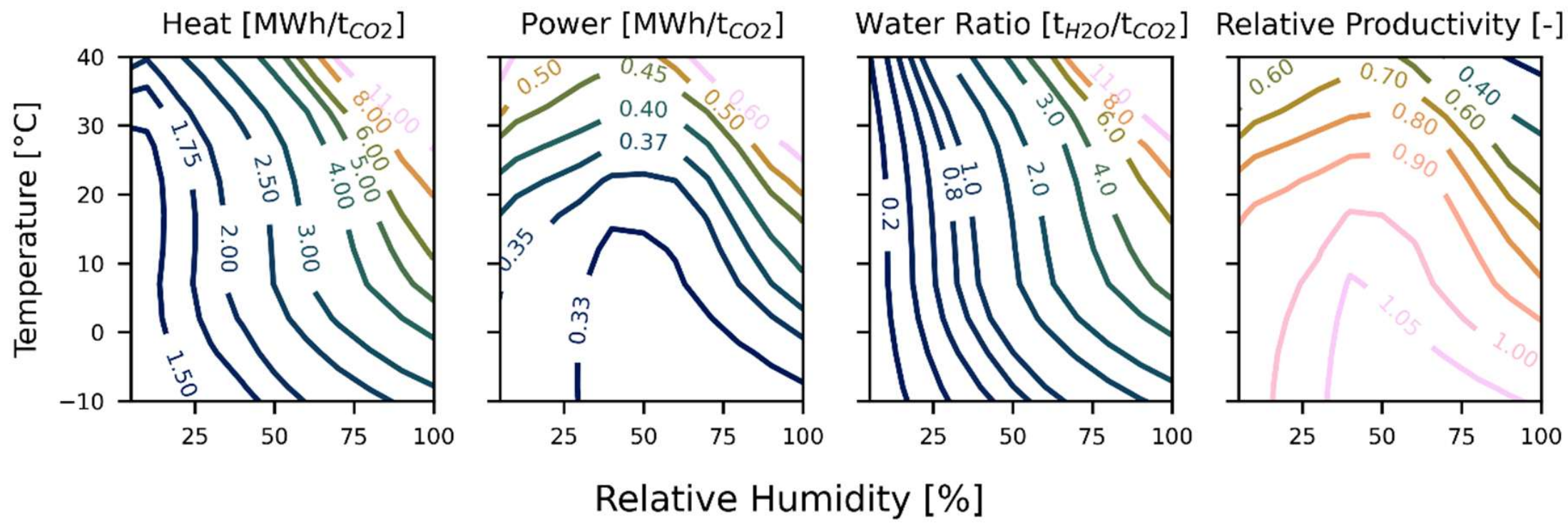

Supplementary Figure 11: Weather-dependent specific energy demand, water co-adsorption ratio and relative productivity from the alternative DAC model[59]. The data was scaled to incorporate future energy demand reductions as described in the main document. The temperature-humidity dependency was evaluated at standard conditions of 1 bar and 420 ppm CO_2 concentration.

Supplementary Note 2

Global Analyses

The global merit order curve (see Supplementary Figure 12) shows a total production potential close to 23 Gt a^{-1} at 10% of the technical potential. Supplying the projected methanol demand of 500 Mt a^{-1} by 2050[60] is feasible at marginal (average) cost of 656 (604) € t^{-1}. Regions with the lowest cost needed for this supply are located in Argentina, Australia, Chile, Egypt, Spain, Greece, Morocco, Mauretania, Portugal, the Philippines, USA, Yemen and South Africa. It should be noted that, since the regions considered are based on administrative boundaries and not equally sized, the production potential of 5 or 10% of the technical potential can differ severely. Therefore, lower production costs could be reached in multiple regions with low cost and high potential, i.e. in Australia.

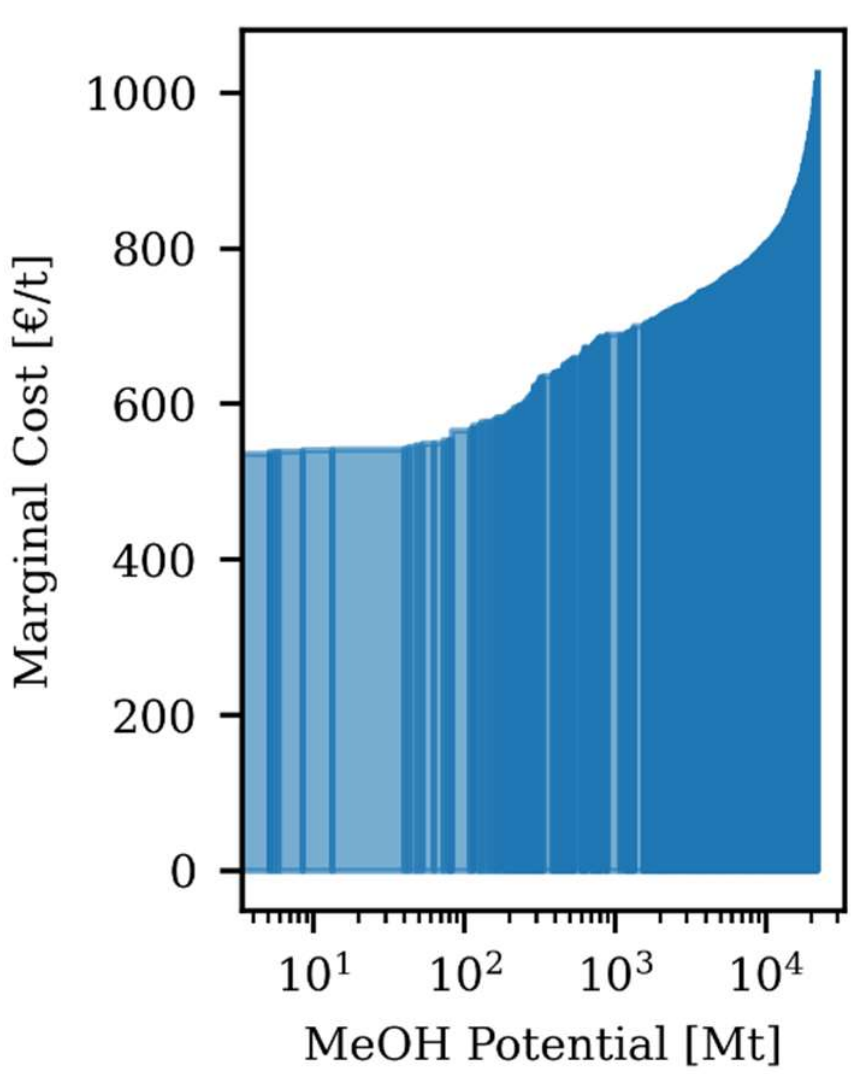

Supplementary Figure 12: Merit order aggregated for all analyzed regions. The total production potential is close to 23 Gt a^{-1} at utilization of 10% of the available technical potential from onshore wind and OFPV plants.

Supplementary Figure 13 shows the relationship between the share of OFPV on the total installed electricity generation capacity (wind + OFPV) and the oversizing ratio of the SOEC, as well as the resulting specific installed battery capacity. It is evident that both the SOEC oversizing as well as the specific installed battery capacity increase as the OFPV share exceeds 50%. Additionally, significant oversizing is also observed at low OFPV shares, especially in regions with unfavorable wind conditions.

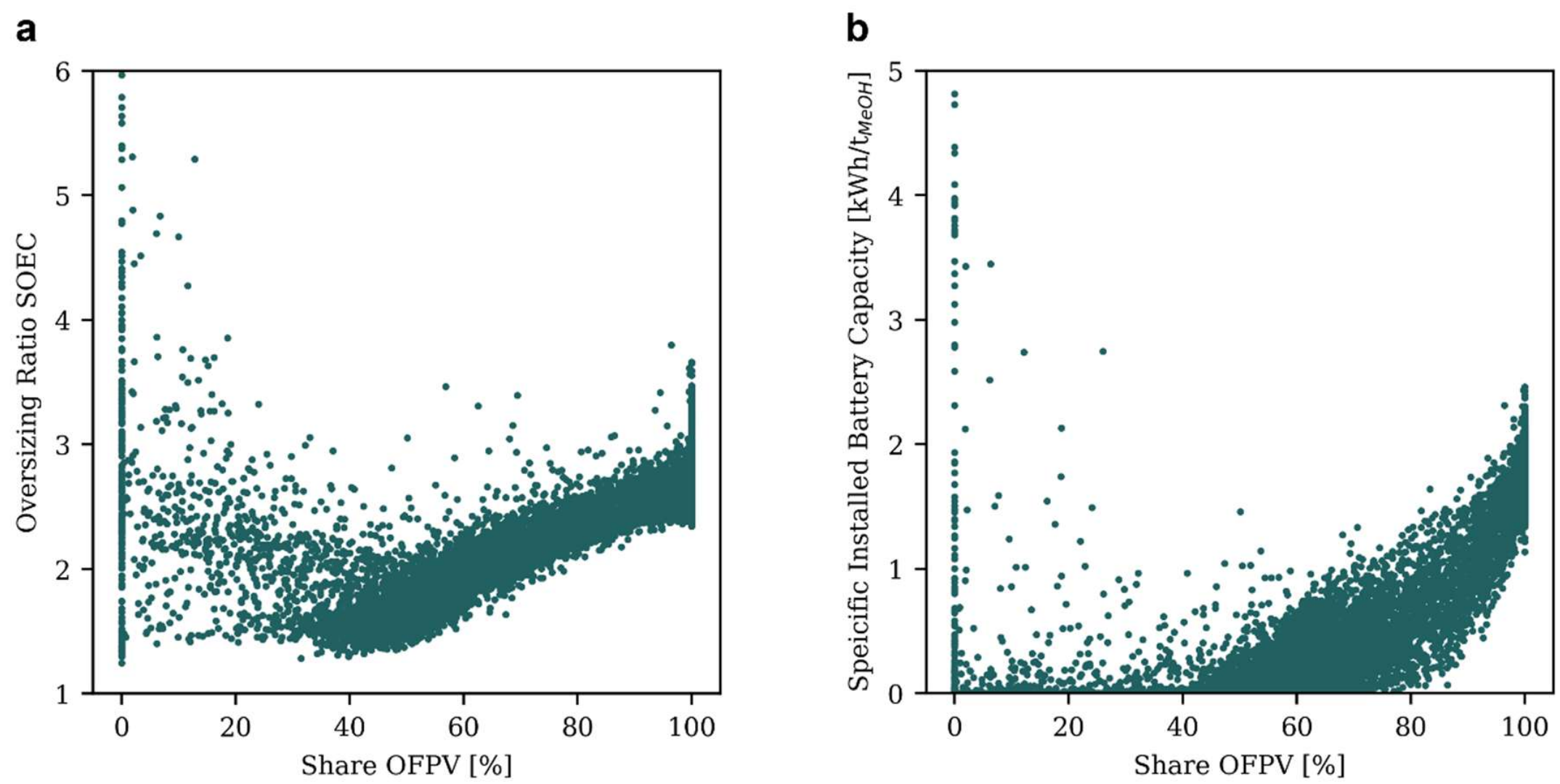

Supplementary Figure 13: Influence of the share of installed OFPV capacity. a. Resulting oversizing ratio of the SOEC. b. Installed specific battery capacity per produced ton of methanol.

Supplementary Figure 14 highlights the LCOM difference comparing the base and the unrestricted scenario with an additional free water source. Most of the 606 regions where no excess water production is observed in the base scenario are landlocked regions.

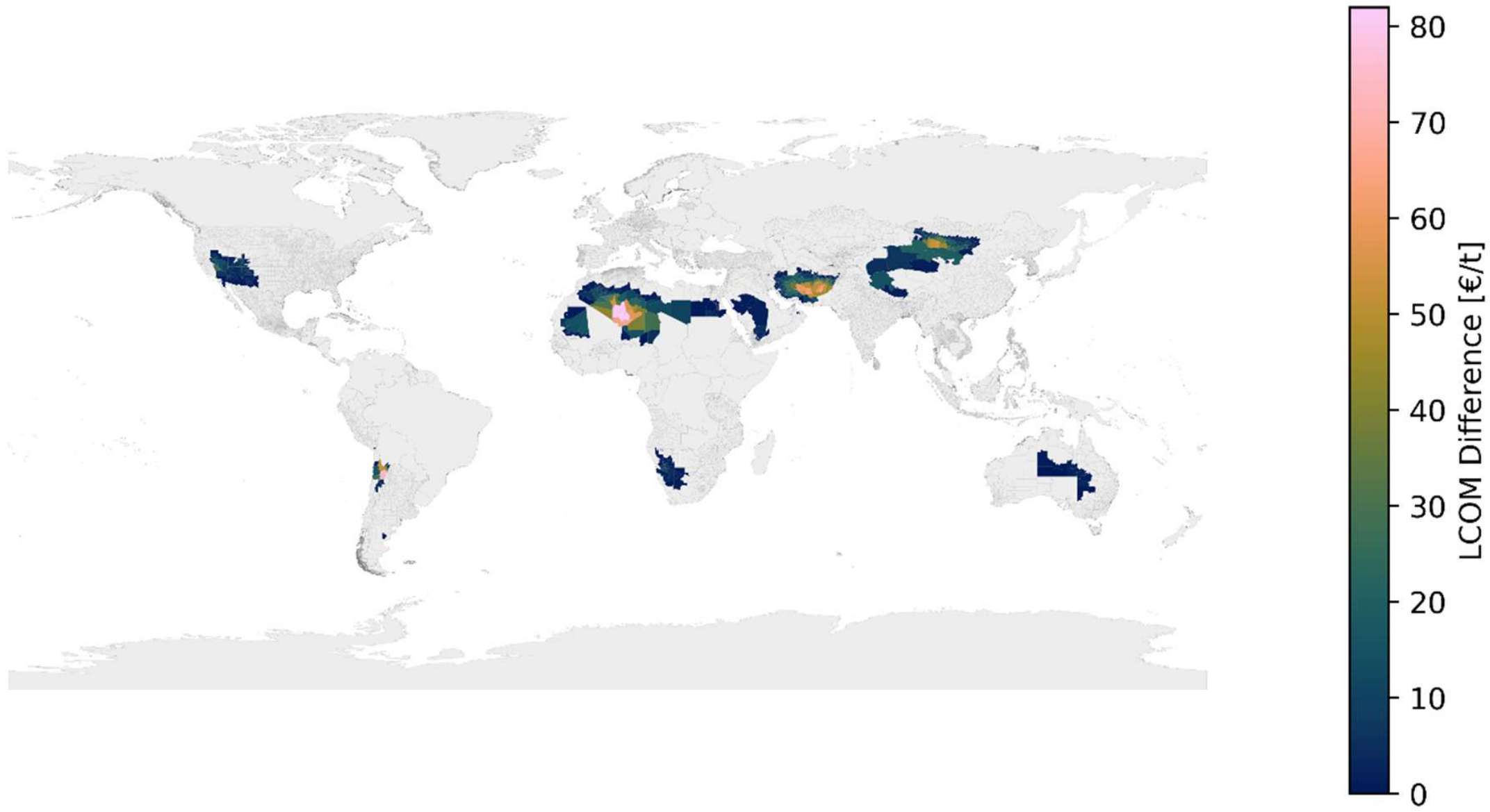

Supplementary Figure 14: LCOM Increase in the base scenario compared to the unrestricted scenario. Only the 606 regions with no excess water production in the base scenario are shown. Country shapes from GADM[56].

Supplementary Figure 15 shows the influence of ambient air temperature on the specific power demand per cooled waste heat. Higher ambient temperature leads to more needed power, as the fans must blow more air through the A-frame for heat transfer.

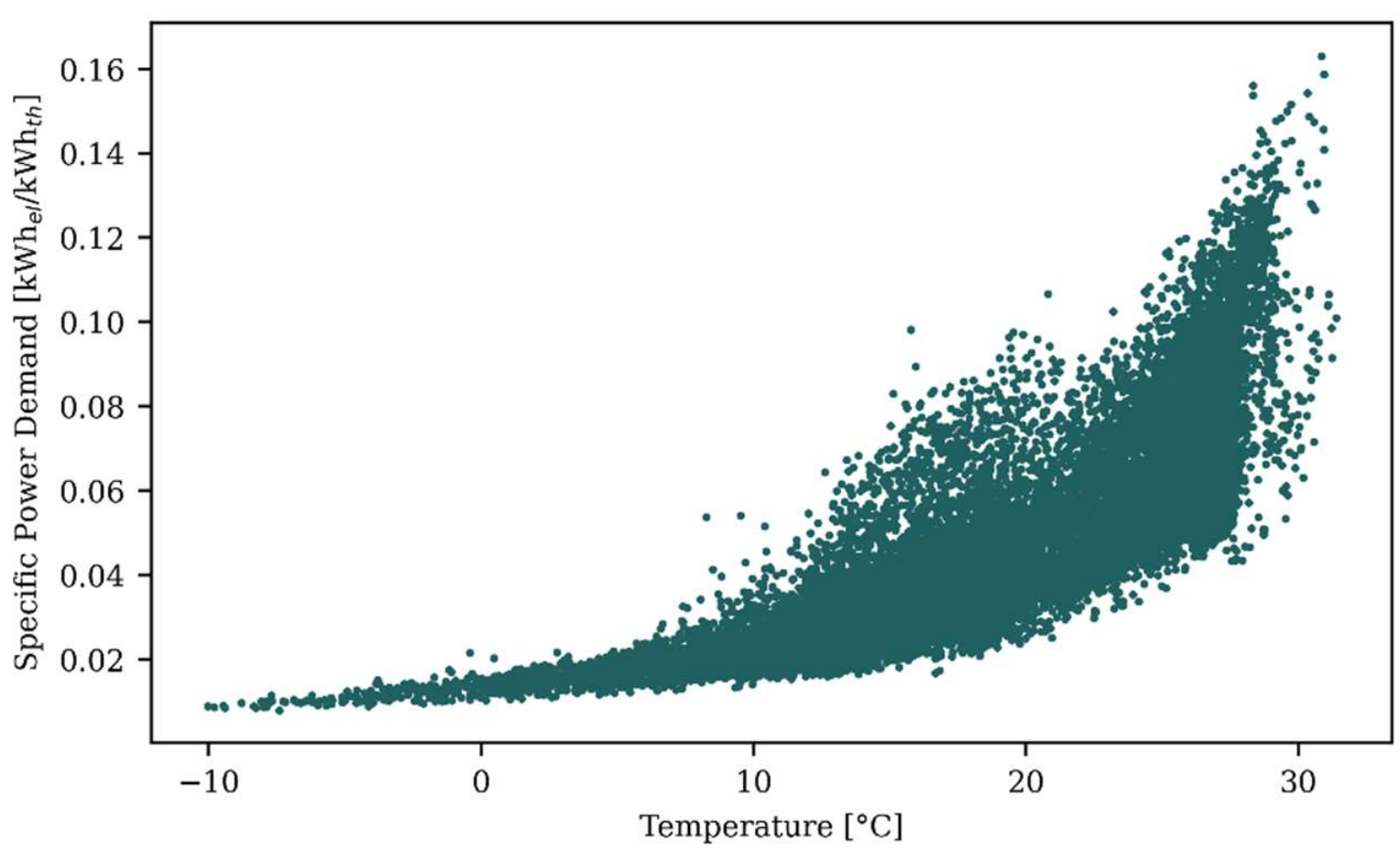

Supplementary Figure 15: Specific power demand of the air cooling system per cooled waste heat.

Supplementary Figure 16 illustrates the effect of a water purification step on the resulting LCOM and excess water production for the PEM-based process chain. For the assessment, conservative assumptions have been used. The capex of the water purification system are assumed to be 2000 € m^{-3} d with an electricity demand of 12 kWh m^{-3} and a water recovery rate of 75% based on Mendler et al.[61]. In general, the water purification system requires only about 0.2% of the total energy demand and the cost contribution is about 0.3-0.5%.

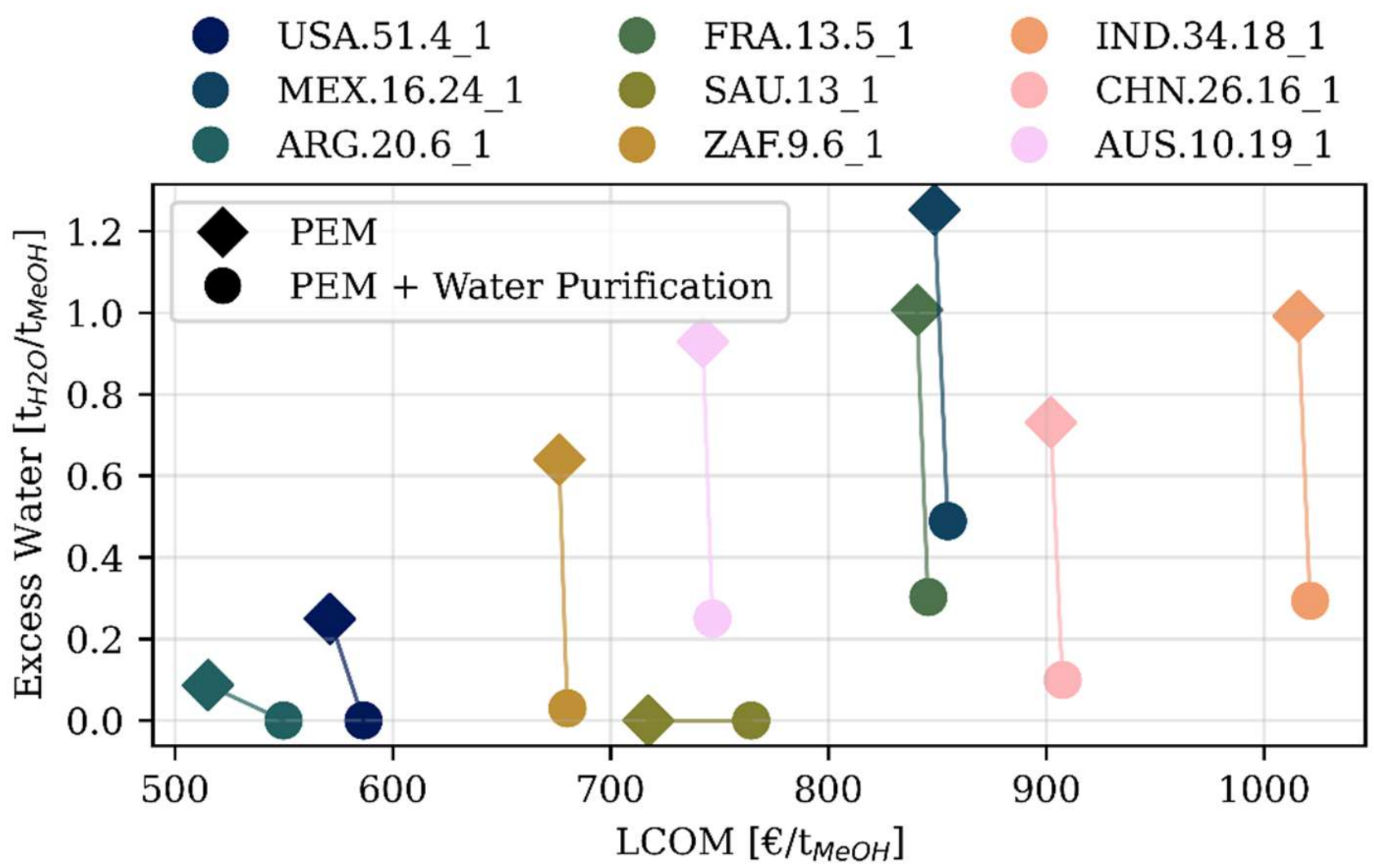

Supplementary Figure 16: Influence of a water purification step on resulting LCOM and excess water production for the PEM-based process chain in nine selected regions.

Operation in Selected Regions

Supplementary Figure 17 shows the operation of selected system components in three illustrative regions. It is observed that system operation generally follows wind and PV power generation and battery storage is only employed for increasing DAC operation. Supplementary Figure 18 shows the operation of the heat system in the same three regions. Generally, electric boilers are only operated during times of high power supply by OFPV or wind turbines.

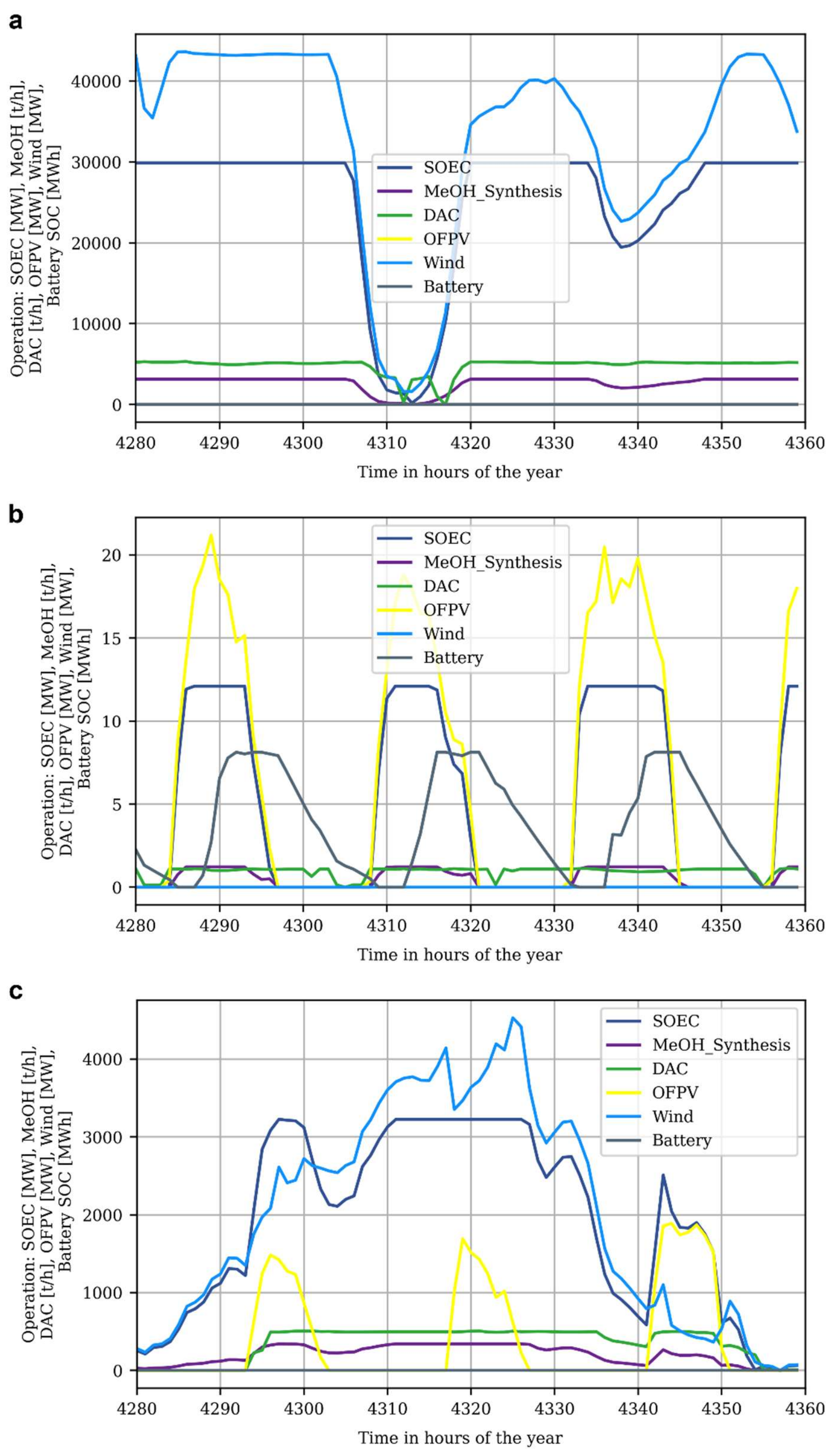

Supplementary Figure 17: Operation of some system components in three illustrative regions for 80 hours during the middle of the year. a. Operation in ARG.20.6_1 where a wind-based system is cost-optimal. b. Operation in MEX.16.24_1 where no wind potential is available. c. Operation in AUS.10.19_1 where a hybrid system results in the lowest cost.

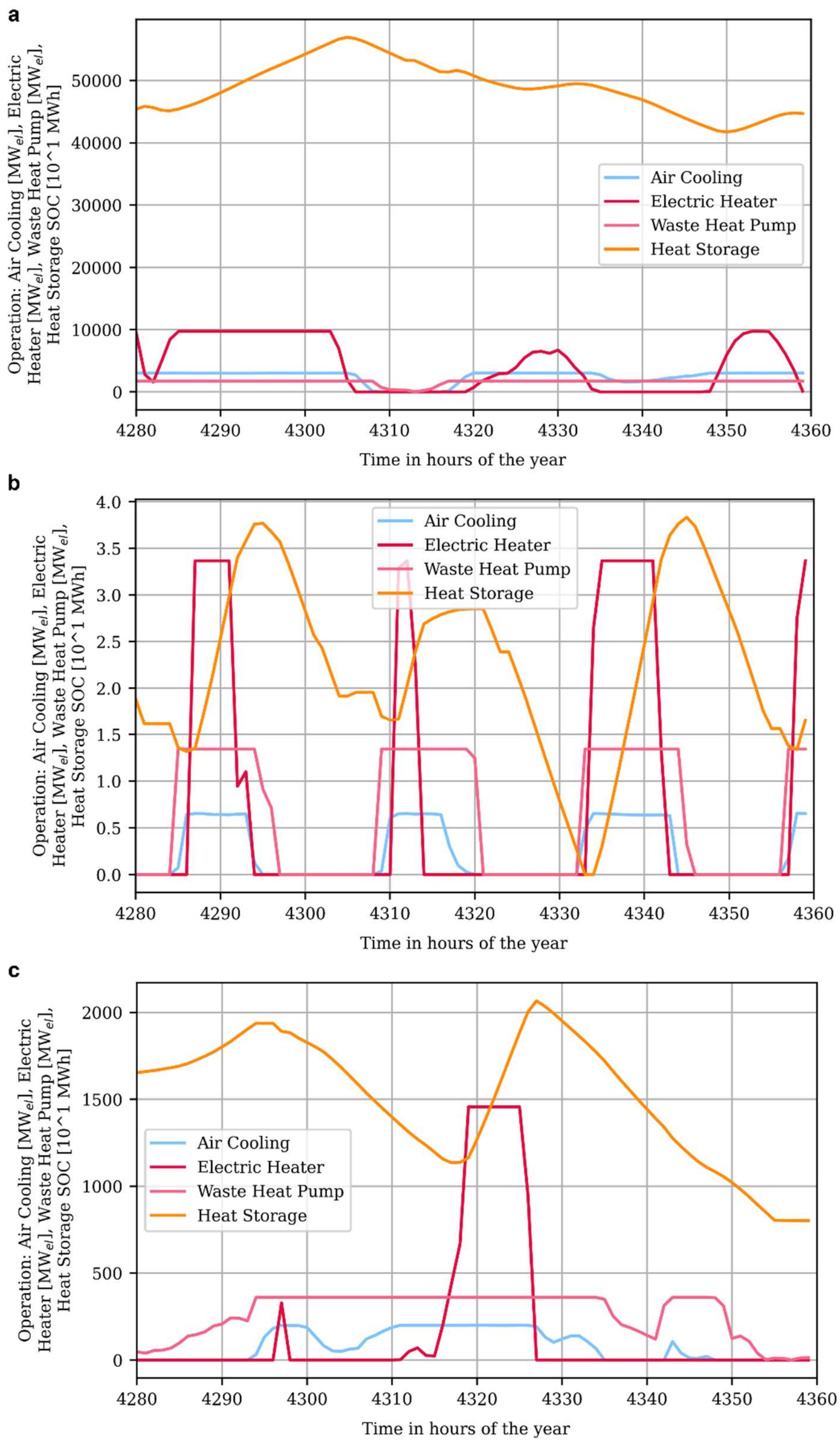

Supplementary Figure 18: Operation of the heat system in the same three regions as in Supplementary Figure 17. a. Operation in ARG.20.6_1 where a wind-based system is cost-optimal. b. Operation in MEX.16.24_1 where no wind potential is available. c. Operation in AUS.10.19_1 where a hybrid system results in the lowest cost.

Supplementary References

(1) Lucas, K. *Thermodynamik: Die Grundgesetze der Energie- und Stoffumwandlungen ; mit 20 Tabellen*, 5., korrigierte und erw. Aufl.; Springer-Lehrbuch; Springer: Berlin, Heidelberg, 2006.
(2) Wenzel, H.*; et al.* Towards water-conscious green hydrogen and methanol production: A techno-economic review. *Nexus* **2,** 100054 (2025).
(3) Gholizadeh, T.; Ghiasirad, H.; Skorek-Osikowska, A. Life cycle and techno-economic analyses of biofuels production via anaerobic digestion and amine scrubbing CO2 capture. *Energy Conversion and Management* **321,** 119066 (2024).
(4) Zhou, H.; Cao, A.; Meng, W.; Wang, D.; Li, G.; Yang, S. Process integration and analysis of coupling solid oxide electrolysis cell (SOEC) and CO2 to methanol. *Energy* **307,** 132652 (2024).
(5) Djettene, R.; Dubois, L.; Duprez, M.-E.; Weireld, G. de; Thomas, D. Integrated CO2 capture and conversion into methanol units: Assessing techno-economic and environmental aspects compared to CO2 into SNG alternative. *Journal of CO2 Utilization* **85,** 102879 (2024).
(6) León, D.; Amez, I.; Castells, B.; Ortega, M. F.; Bolonio, D. Techno-economic analysis of the production of synthetic fuels using CO2 generated by the cement industry and green hydrogen. *International Journal of Hydrogen Energy* **80,** 406–417 (2024).
(7) Li, Q.; Machida, H.; Feng, Z.; Norinaga, K. LNG Oxy-Fuel Power and Green Methanol Poly Generation Systems Associated with CO 2 Condensation Capture: Techno-Economic Environmental Assessment. *Energy Fuels* **38,** 13089–13103 (2024).
(8) Lozano Sanchez, E.; Paulsen, M. M.; Ferrari, F. A.; Pedersen, T. H. Integrated e-Methanol and Drop-in Fuels Hydrothermal Liquefaction Platform-Techno-Economic and GHG Emissions Assessment for Grid-Connected Plants under Flexible BECCU(S) Operation. *Industrial & engineering chemistry research* **63,** 7708–7726 (2024).
(9) Taslimi, M. S.; Khosravi, A.; Nugroho, Y. K.; Rytter, N. G. M. Optimization and analysis of methanol production from CO2 and solar-driven hydrogen production: A Danish case study. *International Journal of Hydrogen Energy* **69,** 466–476 (2024).
(10) Pratama, M. R.; Muthia, R.; Purwanto, W. W. Techno-economic and life cycle assessment of the integration of bioenergy with carbon capture and storage in the polygeneration system (BECCS-PS) for producing green electricity and methanol. *Carb Neutrality* **2** (2023).
(11) Markowitsch, C.; Lehner, M.; Maly, M. Comparison and techno-economic evaluation of process routes for lower olefin production via Fischer–Tropsch and methanol synthesis. *International Journal of Greenhouse Gas Control* **129,** 103985 (2023).
(12) Ong, C. W.; Lin, J.-X.; Tsai, M.-L.; Thoe, K. S.; Chen, C.-L. Techno-economic and carbon emission analyses of a methanol-based international renewable energy supply chain. *International Journal of Hydrogen Energy* **49,** 1572–1585 (2024).
(13) Hao, Q.*; et al.* Zero-energy penalty carbon capture and utilization system based on CLHG integrating SOFC for power and methanol cogeneration. *Energy Conversion and Management* **295,** 117658 (2023).
(14) Rahmat, Y.*; et al.* Techno-economic and exergy analysis of e-methanol production under fixed operating conditions in Germany. *Applied Energy* **351,** 121738 (2023).

(15) Palone, O.; Gagliardi, G. G.; Mechelli, M.; Cedola, L.; Borello, D. Techno-economic analysis of sustainable methanol and ammonia production by chemical looping hydrogen generation from waste plastic. *Energy Conversion and Management* **292,** 117389 (2023).
(16) Zhang, L.*; et al.* Carbon-neutral light olefins production for energy decarbonization: Process development and techno-econo-environmental analysis. *Chemical Engineering Journal* **471,** 144611 (2023).
(17) Gierse, M.; Kerschbaum, M.; Steinbach, B.; Sauer, J.; Salem, O. Optimized design and techno-economic analysis of novel DME production processes. *React. Chem. Eng.* **8,** 2826–2840 (2023).
(18) Khojasteh-Salkuyeh, Y.; Ashrafi, O.; Mostafavi, E.; Navarri, P. CO 2 utilization for methanol production; Optimal pathways with minimum GHG reduction cost. *Can J Chem Eng* **101,** 5446–5459 (2023).
(19) Haghighatjoo, F.; Rahimpour, M. R.; Farsi, M. Techno-economic and environmental assessment of CO2 conversion to methanol: Direct versus indirect conversion routes. *Chemical Engineering and Processing - Process Intensification* **184,** 109264 (2023).
(20) Chiou, H.-H.; Lee, C.-J.; Wen, B.-S.; Lin, J.-X.; Chen, C.-L.; Yu, B.-Y. Evaluation of alternative processes of methanol production from CO2: Design, optimization, control, techno-economic, and environmental analysis. *Fuel* **343,** 127856 (2023).
(21) Kanuri, S.*; et al.* Methanol synthesis from CO2 via hydrogenation route: Thermodynamics and process development with techno-economic feasibility analysis. *Korean J. Chem. Eng.* **40,** 810–823 (2023).
(22) Nyári, J.; Magdeldin, M.; Larmi, M.; Järvinen, M.; Santasalo-Aarnio, A. Techno-economic barriers of an industrial-scale methanol CCU-plant. *Journal of CO2 Utilization* **39,** 101166 (2020).
(23) Nizami, M.; Slamet; Purwanto, W. W. Solar PV based power-to-methanol via direct CO2 hydrogenation and H2O electrolysis: Techno-economic and environmental assessment. *Journal of CO2 Utilization* **65,** 102253 (2022).
(24) Su, C.; Wei, H.; Wang, Z.; Ayed, H.; Mouldi, A.; Shayesteh, A. A. Economic accounting and high-tech strategy for sustainable production: A case study of methanol production from CO2 hydrogenation. *International Journal of Hydrogen Energy* **47,** 25929–25944 (2022).
(25) Lacerda de Oliveira Campos, B.*; et al.* A Detailed Process and Techno-Economic Analysis of Methanol Synthesis from H2 and CO2 with Intermediate Condensation Steps. *Processes* **10,** 1535 (2022).
(26) Wang, D.*; et al.* Integrated Process for Producing Glycolic Acid from Carbon Dioxide Capture Coupling Green Hydrogen. *Processes* **10,** 1610 (2022).
(27) Del Arnaiz Pozo, C.; Cloete, S.; Jiménez Álvaro, Á. Techno-economic assessment of long-term methanol production from natural gas and renewables. *Energy Conversion and Management* **266,** 115785 (2022).
(28) Li, P.; Gong, S.; Li, C.; Liu, Z. Analysis of routes for electrochemical conversion of CO2 to methanol. *Clean Energy* **6,** 202–210 (2022).
(29) Cordero-Lanzac, T.*; et al.* A techno-economic and life cycle assessment for the production of green methanol from CO2: catalyst and process bottlenecks. *Journal of Energy Chemistry* **68,** 255–266 (2022).
(30) Lee, B.*; et al.* Economic Parity Analysis of Green Methanol Synthesis Using Water Electrolysis Based on Renewable Energy. *ACS Sustainable Chem. Eng.* **9,** 15807–15818 (2021).

(31) Gu, Y.; Wang, D.; Chen, Q.; Tang, Z. Techno-economic analysis of green methanol plant with optimal design of renewable hydrogen production: A case study in China. *International Journal of Hydrogen Energy* **47,** 5085–5100 (2022).
(32) Wu, T.-W.; Chien, I.-L. A novel energy-efficient process of converting CO2 to dimethyl ether with techno-economic and environmental evaluation. *Chemical Engineering Research and Design* **177,** 1–12 (2022).
(33) Khunathorncharoenwong, N.; Charoensuppanimit, P.; Assabumrungrat, S.; Kim-Lohsoontorn, P. Techno-economic analysis of alternative processes for alcohol-assisted methanol synthesis from carbon dioxide and hydrogen. *International Journal of Hydrogen Energy* **46,** 24591–24606 (2021).
(34) Battaglia, P.; Buffo, G.; Ferrero, D.; Santarelli, M.; Lanzini, A. Methanol synthesis through CO2 capture and hydrogenation: Thermal integration, energy performance and techno-economic assessment. *Journal of CO2 Utilization* **44,** 101407 (2021).
(35) Adnan, M. A.; Kibria, M. G. Comparative techno-economic and life-cycle assessment of power-to-methanol synthesis pathways. *Applied Energy* **278,** 115614 (2020).
(36) Zhang, J.; Li, Z.; Zhang, Z.; Feng, K.; Yan, B. Can thermocatalytic transformations of captured CO2 reduce CO2 emissions? *Applied Energy* **281,** 116076 (2021).
(37) Nyári, J.; Izbassarov, D.; Toldy, Á. I.; Vuorinen, V.; Santasalo-Aarnio, A. Choice of the kinetic model significantly affects the outcome of techno-economic assessments of CO2-based methanol synthesis. *Energy Conversion and Management* **271,** 116200 (2022).
(38) Atsbha, T. A.; Yoon, T.; Yoo, B.-H.; Lee, C.-J. Techno-Economic and Environmental Analysis for Direct Catalytic Conversion of CO2 to Methanol and Liquid/High-Calorie-SNG Fuels. *Catalysts* **11,** 687 (2021).
(39) Vo, C. H.; Mondelli, C.; Hamedi, H.; Pérez-Ramírez, J.; Farooq, S.; Karimi, I. A. Sustainability Assessment of Thermocatalytic Conversion of CO 2 to Transportation Fuels, Methanol, and 1-Propanol. *ACS Sustainable Chem. Eng.* **9,** 10591–10600 (2021).
(40) Cavalcanti, E. J.; Motta, H. P. Exergoeconomic analysis of a solar-powered/fuel assisted Rankine cycle for power generation. *Energy* **88,** 555–562 (2015).
(41) IEA. *The Future of Hydrogen: Seizing today's opportunities*, 2019. https://www.iea.org/reports/the-future-of-hydrogen.
(42) IRENA. *Hydrogen from renewable power: Technology outlook for the energy transition*, 2018.
(43) National Renewable Energy Laboratory. *2023 Annual Technology Baseline*, 2023. https://atb.nrel.gov/electricity/2023/technologies.
(44) Grosse, R.; Christopher, B.; Stefan, W.; Geyer, R.; Robbi, S. *Long term (2050) projections of techno-economic performance of large-scale heating and cooling in the EU*; Publications Office: Luxembourg, 2017.
(45) Ellersdorfer, P.; Omar, A.; Rider, I.; Daiyan, R.; Leslie, G. The hydrogen-water collision: Assessing water and cooling demands for large-scale green hydrogen production in a warming climate. *International Journal of Hydrogen Energy* **97,** 1002–1013 (2025).
(46) Department for Business, Energy and Industrial Strategy. *Shipping CO2 - UK cost estimation study*, 2018. https://www.gov.uk/government/publications/shipping-carbon-dioxide-co2-uk-cost-estimation-study.
(47) Deng, H.; Roussanaly, S.; Skaugen, G. Techno-economic analyses of CO2 liquefaction: Impact of product pressure and impurities. *International Journal of Refrigeration* **103,** 301–315 (2019).

(48) Cau, G.; Cocco, D.; Serra, F. Energy and cost analysis of small-size integrated coal gasification and syngas storage power plants. *Energy Conversion and Management* **56,** 121–129 (2012).
(49) Khan, M. A.; Young, C.; MacKinnon, C.; Layzell, D. *The Techno-Economics of Hydrogen Compression* No. 1; Transition Accelerator Technical Briefs, 2021. https://transitionaccelerator.ca/wp-content/uploads/2023/04/TA-Technical-Brief-1.1_TEEA-Hydrogen-Compression_PUBLISHED.pdf.
(50) Roman, J.; Wróblewski, R.; Klojzy-Karczmarczyk, B.; Ceran, B. Energetic, Economic and Environmental (3E) Analysis of a RES-Waste Gasification Plant with Syngas Storage Cooperation. *Energies* **16,** 2062 (2023).
(51) Stolten, D.*; et al. Neue Ziele auf alten Wegen? Strategien für eine treibhausgasneutrale Energieversorgung bis zum Jahr 2045*; Reihe Energie & Umwelt / Energy & Environment No. 577; Schriften des Forschungszentrums Jülich, 2022.
(52) IRENA. *Innovation outlook: Thermal energy storage*, 2020.
(53) Batchelor, C.; Fonseca, C.; Smits, S. *Life-cycle costs of rainwater harvesting systems*, 2011. https://www.ircwash.org/sites/default/files/Batchelor-2011-Lifecycle.pdf.
(54) Hinkley, J. T.; McNaughton, R. K.; Pye, J.; Saw, W.; Stechel, E. B. The challenges and opportunities for integration of solar syngas production with liquid fuel synthesis. *AIP Conference Proceedings (2016)*.
(55) Hersbach, H.*; et al.* The ERA5 global reanalysis. *Quart J Royal Meteoro Soc* **146,** 1999–2049 (2020).
(56) GADM. Database of global administrative areas. https://gadm.org/index.html.
(57) Winkler, C.*; et al.* Participatory mapping of local green hydrogen cost-potentials in Sub-Saharan Africa. *International Journal of Hydrogen Energy* **112,** 289–321 (2025).
(58) IEA. *Global Hydrogen Review 2024*, 2024. https://www.iea.org/reports/global-hydrogen-review-2024.
(59) Cai, X.; Coletti, M. A.; Sholl, D. S.; Allen-Dumas, M. R. Assessing Impacts of Atmospheric Conditions on Efficiency and Siting of Large-Scale Direct Air Capture Facilities. *JACS Au* **4,** 1883–1891 (2024).
(60) IRENA; Methanol Institute. *Innovation outlook: Renewable Methanol*, 2021. https://www.irena.org/-/media/Files/IRENA/Agency/Publication/2021/Jan/IRENA_Innovation_Renewable_Methanol_2021.pdf.
(61) Mendler, R.*; et al. Water Supply for Electrolysis Plants: HYPAT Working Paper*, 2024. https://hypat.de/hypat-wAssets/docs/new/publikationen/HYPAT_WP_Water-Supply-for-Electrolysis-Plants.pdf.
