# Supplementary material for: Direct air capture enables sustainable methanol production in water-scarce regions
Source: Nat Commun. 2026 Aug 24;17:8495. doi: 10.1038/s41467-026-76865-x (PMC13503827; doi:10.1038/s41467-026-76865-x)
Supplement: Supplementary file 2 [file 41467_2026_76865_MOESM2_ESM.pdf]

nature portfolio

Peer Review File

Direct air capture enables sustainable methanol production in water-scarce regions

Corresponding Author: Mr Henrik Wenzel

This file contains all reviewer reports in order by version, followed by all author rebuttals in order by version.

Version 0:

Reviewer comments:

Reviewer #1

(Remarks to the Author)
This paper reports an investigation on a novel methanol production process where water and CO2 feedstocks are from direct air capture. The contribution is significant, as methanol production costs in a substantial number of regions (>20,000) were studied. Key factors that may impact the performance of the novel process were also identified. The paper is well written. Here are some comments to be considered.

1. The title of the article makes a statement that exaggerates the outcomes/benefits of the process and is not 100% accurate. For a scientific paper, the title should be technically correct and not ambiguous. Hence, I don't think that the title is a good choice.

2. In results and discussions, words such as "favorable" and "suitable" were used. However, there is no discussion on why these costs or regions were considered "favorable" and "suitable."

3. The discussions on the effects of different parameters left me the impression that correlations were found from the plots and then the authors attempted to find reasons for these observed correlations. Therefore, I think that discussions on the limitation of drawing conclusions from these observations should be discussed in the discussion section or in the section "Limitations and Outlook"

4. The production scale in different regions was discussed. However, the techno-economic parameters were fixed values, and the economic and efficiency performances of scaling up/down the technologies were not considered. In addition, labor costs of all these different regions were not considered. These may lead to inaccurate conclusions.

5. The price of fossil-based methanol was mentioned in the "Discussion" section. I would suggest the authors mention it in the introduction or at the beginning of the results section to allow readers understand how the levelized costs were compared with the current methanol price.

Reviewer #2

(Remarks to the Author)
As communicated to the editor before accepting, I coincidentally reviewed this paper earlier for another journal (only first submission, not the revision). I am pasting my original review below for transparency. I am therefore looking at this new version from a second review round perspective.
From what I can see without a rebuttal, I think the authors have done a good work in tackling my earlier comments; however, some points require a bit more attention. More specifically:
- Concerns on the DAC model: the original model used by the authors is now public (ref [21] in the manuscript), but it is not peer reviewed. When quickly scanning through the preprint paper, some concerns remain: the model does not look validated, the DAC cycle is not reported, and Aspen Adsorption can be problematic, for example in closing the mass balance due the finite difference approach. However, authors have added a new section in the manuscript where they test the DAC-to-MeOH optimization model using a different DAC thermodynamic model (from the group of D. Sholl, now co-author of the submitted manuscript). The water-related results presented in the manuscript look robust to different DAC models, which is indeed good for the findings. However, there are significant differences in terms of energy and costs, which

authors duly recognize and discuss. The question that remains open is: which of the two models is more likely to reproduce the physical behavior of DAC? Or, which are the reasons behind this differences (assumptions, modelling choices, sorbent isotherms)? While authors do not enter into a detailed comparison, the outcomes of the DAC-to-MeOH optimization model still depend on how DAC behaves at varying ambient conditions. Therefore, I would like to see authors going a bit more in depth here.
- On a similar note, authors have not tackled my comment on water recovery: from my understanding, they assume that all water separated is released with CO2. This however is not necessarily true: it depends on the DAC cycle adopted and the kinetics of CO2 and H2O. This information is not available in the SI either. Authors should discuss this point and ideally test what happens when only part of the separated water can be recovered.

PREVIOUS REVISION:
I read with interest the manuscript submitted by Wenzel and coauthors, which investigates the (global) potential of producing methanol using as feedstock CO2 and water removed from the atmosphere using direct air capture. Authors use a fairly sophisticated modelling framework (Ethos.fine) to optimize the process in space (20’000 locations) and time (year with hourly discretization). I find the approach of evaluating a process that is highly dependent on time series data (renewables, direct air capture) using a time- and space-discretized model excellent. The topic, namely the production of green, net-zero methanol, is overall of interest, yet certainly not new in the scientific area of process and energy systems (authors do a rather fair work in citing several relevant studies, as Fasihi and Breyer). However, I believe the study does not meet the standards for publication in **** for the following key reasons.
- Relevance of the study. The key point of this study is to investigate methanol production in a process that is water autarchic, i.e. where water is supplied by the very DAC process itself. Apart from modelling issues, which I highlight below, I wonder if the proposed process solution is realistic or rather an academic exercise. The reason for this doubt is, that water supply for such an industrial process is likely to come from a very reasonable alternative source, seawater desalination, as far as the plant is conveniently located nearby the coast, which does not seem unrealistic given supply chain logics, offshore wind potential, and access to the coast for many of the locations considered. Seawater desalination, especially when using reverse osmosis membranes, is applied in thousands of plants worldwide at different scales. It consumes and costs a negligible fraction of the energy and CAPEX needed for DAC+SOEC, and it provides an independent, reliable clean water flow that does not depend on DAC operation. All these properties would be difficult to ignore for an industrial project developer. This point therefore reduces the relevance of the study to water distressed places that don’t have access to seawater. But then one may wonder how many cases exist, especially when considering that the DAC-based water supply is particularly convenient close to humid coastlines (Fig 4a).
- Modelling of DAC: my second, but probably largest concern arises from the modelling of DAC. All results presented in this paper concerning the DAC operation under weather varying conditions boil down to the question: is the DAC model correct? Unfortunately, authors refer to an own work that is currently in preparation. As it is not possible to answer this question, I believe the paper does not fit in ****. Points that are of particular concern in the model and that I could not find answer to include:
o Isotherms and kinetics: in their previous work [ref 8], authors used performance for weather dependent DAC from two 2022 works: Sendi et al. (One Earth 2022) and Wiegner et al. (ind.Eng.Chem.Res 2022). Both works use proper H2O and CO2 isotherms, but they were carried out at a time when no experimental data was available for mass transfer (linear driving force in adsorption models). Authors can check recent works from the groups of, e.g., Rajendran (U.Alberta), Pini/Petit (Imperial), Mazzotti (ETH Zurich), Lively (Georgia Tech) for an overview of the state of the art on this topic. A proper use of current scientific knowledge on the linear driving force is vital for predicting the performance of DAC at different ambient conditions (e.g. mass transfer is slower at colder temperature, water affects the kinetics). Eventually, mass transfer will tell when and what is adsorbed/desorbed.
o Cycle and production: following the previous point, authors assume that all water comes out with CO2 in the cycle. This is a critical assumption for water recovery. However, this is not necessarily the case. Part of the water will get out with nitrogen during pre-heating. Another part will come out later after CO2 is desorbed. Kinetics and cycle design will govern this behavior, and I am afraid this is not considered in this study – or at least it is not possible to evaluate what was done.
Eventually these last two points make the reviewer wondering if the excellent time- and space-discretize modelling of the process is in reality replicating the physical behavior of the separation. Without a clear answer to this point, I am afraid the work does not fit in **** in the present form. I would be open to reconsider my decision were the previous points be adequately addressed.

Reviewer #3

(Remarks to the Author)
the manuscript offers a compelling paradigm shift: utilizing DAC's water co-adsorption as the sole feedstock for downstream electrolysis and synthesis. The manuscript is well-organized and demonstrates high research value. However, several key aspects regarding dynamic operation, regional economic disparities, and equipment degradation require further justification and clarification before publication.
（1） The study assumes fully flexible operation of the SOEC model to match intermittent renewable energy generation. However, there is a known correlation between the lifespan of SOECs and the frequency of start-up/shut-down cycles. How do the authors account for the impact of SOEC replacement costs, driven by accelerated degradation, on the final LCOM?
（2） The air-cooling model assumes that operation is not possible at ambient temperatures above 35 °C. However, in arid regions highly suitable for this technology (e.g., the Middle East, Australia), summer daytime temperatures frequently exceed 40 °C. Under such conditions, what is the operational status of the downstream methanol synthesis plant? Does it shut down

entirely? Furthermore, how is the thermal inertia of the reactors managed during these extreme temperature periods?
(3) The study assumes that water captured via DAC can be directly utilized in the SOEC. Have the authors considered the impact of impurities typically present in such water (e.g., volatile organic compounds, atmospheric dust particles) on subsequent modules and production stages? The necessity of a water purification step would likely affect the overall system's energy efficiency and capital costs.
(4) In arid desert regions where this technology appears most promising, dust storms and poor air quality are common. Would these harsh conditions affect the fan efficiency and filter integrity of the DAC technology? Have the equipment maintenance costs associated with these severe environmental factors been incorporated into the economic model?
(5) The study calculates heat integration based on the total energy balance. However, the main system units operate on different dynamic profiles: the DAC operates on an intermittent/cyclic basis, whereas the SOEC and methanol synthesis ideally operate continuously. Is the proposed heat integration technically feasible without the support of large-capacity thermal energy storage?
(6) It is noted that in the green methanol synthesis unit, the purge gas is combusted to provide heat for upstream processes. Does this step generate significant CO_2 emissions? Has a carbon capture process been considered for this specific stream, or is this carbon loss accounted for in the overall system carbon balance and specific DAC capacity sizing?
(7) The sensitivity analysis indicates that a PEM-based system achieves a lower LCOM across all regions. Given this substantial economic advantage, why was the SOEC chosen as the primary technology over PEM in the main baseline assessment?
(8) A fixed discount rate of 8% is applied. However, the Weighted Average Cost of Capital (WACC) varies significantly between regions (e.g., the USA vs. Mauritania). Although mentioned in the discussion, presenting a base case with a uniform WACC obscures the real-world economic barriers present in developing nations.
(9) The calculated LCOM represents the cost strictly at the production site. Arid regions rich in wind and solar resources are often remote and far from deep-water ports or industrial hubs. How would the costs of new infrastructure (pipelines, rail, or transmission grids) impact these economic results?
(10) In regions with higher relative humidity, excess water production exceeds 2.5 $tH_2O/tMeOH$. Particularly in inland environments, have the environmental or infrastructure costs associated with the discharge, treatment, or storage of this massive volume of excess water been considered?
(11) The manuscript mentions that the ERA5 weather data has a spatial resolution of 0.25°×0.25°. Because microclimates in coastal or mountainous arid regions can vary drastically within a ~30 km grid, please discuss the potential margin of error introduced by this resolution on the DAC performance metrics.
(12) The manuscript mentions using buffer storage during times of insufficient ambient air water supply. What is the assumed maximum retention time for this water storage? Furthermore, how does the factor of evaporation loss impact the capacity sizing and economics of this storage?

Version 1:

Reviewer comments:

Reviewer #1

(Remarks to the Author)
The revised manuscript has addressed all my comments.

(Remarks on code availability)

Reviewer #2

(Remarks to the Author)
I would like to thank the authors for the detailed rebuttal and the associated revision of the comments received from all referees. As for my comments, I find the rebuttal and the changes in the text satisfactory. The manuscript can therefore be accepted for publication.

(Remarks on code availability)

Reviewer #3

(Remarks to the Author)
The authors have comprehensively addressed all concerns raised in the previous review. They supplemented sufficient supplementary data, clarified ambiguous descriptions, and reasonably responded to every point of the reviewers' comments point-by-point.
The novelty and significance of the research fit the scope and standards of Nature Communications, and the revised manuscript has substantially improved in readability, argumentation integrity and presentation quality. No remaining major or minor issues require further revision. I recommend the manuscript for acceptance.

(Remarks on code availability)

Open Access This Peer Review File is licensed under a Creative Commons Attribution 4.0 International License, which permits use, sharing, adaptation, distribution and reproduction in any medium or format, as long as you give appropriate credit to the original author(s) and the source, provide a link to the Creative Commons license, and indicate if changes were made.
In cases where reviewers are anonymous, credit should be given to 'Anonymous Referee' and the source.
The images or other third party material in this Peer Review File are included in the article's Creative Commons license, unless indicated otherwise in a credit line to the material. If material is not included in the article's Creative Commons license and your intended use is not permitted by statutory regulation or exceeds the permitted use, you will need to obtain permission directly from the copyright holder.
To view a copy of this license, visit https://creativecommons.org/licenses/by/4.0/

Dear Editor and Reviewers,

Thank you for your valuable comments and suggestions on our manuscript. We have substantially revised our work to ensure scientific rigor, a clear presentation of our findings and robustness of our conclusions. Thus, we have carefully addressed each of your points and have marked all changes in the manuscript. To better track changes, we have additionally included a comprehensive overview (see below) for a detailed breakdown of your comments, our responses, and the changes we've made.

Yours sincerely, on behalf of all the co-authors,

Henrik Wenzel

	**Comments**	**Authors response**	**Changes made, if any**
	**Reviewer #1:**		
	This paper reports an investigation on a novel methanol production process where water and CO2 feedstocks are from direct air capture. The contribution is significant, as methanol production costs in a substantial number of regions (>20,000) were studied. Key factors that may impact the performance of the novel process were also identified. The paper is well written. Here are some comments to be considered.	Thank you for your positive evaluation.	
1	The title of the article makes a statement that exaggerates the outcomes/benefits of the process and is not 100% accurate. For a scientific paper, the title should be technically correct and not ambiguous. Hence, I don't think that the title is a good choice.	Thank you. We do agree that the title was not 100% accurate, given that we have not analyzed hydrocarbons in general. Therefore, we have adapted it accordingly.	The new title of the manuscript is: "Direct air capture enables sustainable methanol production in water-scarce regions"<br><br>Accordingly, we have adapted the last sentence of the abstract.
2	In results and discussions, words such as "favorable" and "suitable" were used. However, there is no discussion on why these costs or regions were considered "favorable" and "suitable."	Thank you. In the initial version of the manuscript, we used the terms "favorable" and "suitable" interchangeably to describe regions with relatively low production costs or good conditions. We agree that this wording was imprecise. The terminology has therefore been revised throughout the manuscript to	We have adapted the wording on multiple (marked) parts of the manuscript. Additionally, we introduce the convention of "suitable" regions to refer to regions where DAC can supply sufficient water without adaptations in the section on "Water Supply and Imposed Constraints": "Therefore, these regions

		distinguish the two concepts more clearly: “favorable” now refers specifically to regions with comparatively low production costs, whereas “suitable” refers to regions where the water supply from DAC is sufficient to meet process requirements without additional water-related constraints.	are considered suitable for DAC-based production, as the water demand can be fully met through atmospheric capture without additional water-related constraints.”
3	The discussions on the effects of different parameters left me the impression that correlations were found from the plots and then the authors attempted to find reasons for these observed correlations. Therefore, I think that discussions on the limitation of drawing conclusions from these observations should be discussed in the discussion section or in the section "Limitations and Outlook"	Thank you. Indeed, we analyzed the results and then investigated the reasons which lead to the observed correlations. We do acknowledge that correlation does not necessarily imply causation. However, most of the observed effects are well understood and we do believe that the conclusions drawn are still valid. For the novel findings of our study, especially regarding the effect of weather on DAC or the total system, we have included a statement addressing this point.	We have included a statement into our results section where we analyze the effect of weather on DAC: “While the geospatial analysis reveals clear relationships between weather conditions, cost contributions and system efficiency, these observations should primarily be interpreted as correlations rather than direct causal relationships. The proposed explanations are based on the underlying process mechanisms represented in the model – however, multiple interdependent factors simultaneously influence optimal system design and operation, meaning that individual parameter effects cannot always be isolated clearly. This limitation should also be considered in the following assessment of water supply from DAC.”
4	The production scale in different regions was discussed. However, the techno-economic parameters were fixed values, and the economic and efficiency performances of scaling up/down the technologies were not considered. In addition, labor costs of all these different regions were not considered. These may lead to inaccurate conclusions.	Thank you. In this study, techno-economic parameters were intentionally kept constant across regions to isolate and evaluate the influence of climatic conditions and renewable resource availability on system performance. Consequently, regional differences in labor cost and potential scale-dependent variations were not explicitly considered. We acknowledge that economies of scale and regional economic factors may influence both cost and efficiency, and therefore the reported results should be interpreted as a comparative assessment of the feasibility of DAC-based feedstock provision across different regions.	A corresponding discussion has been added to the limitations section: “Besides technology specific limitations, our study applies uniform techno-economic assumptions across all regions to isolate the effects of weather conditions and renewable energy availability. Consequently, regional differences in labor costs, financing conditions and potential scale-dependent technology performance are not explicitly represented. In reality, economies of scale and regional economic conditions may affect both system cost and efficiency. Therefore, the presented results should primarily be interpreted as a comparative assessment of the feasibility of DAC-based feedstock provision across different regions.”

5	The price of fossil-based methanol was mentioned in the "Discussion" section. I would suggest the authors mention it in the introduction or at the beginning of the results section to allow readers understand how the levelized costs were compared with the current methanol price.	Thank you. We agree and now mention the price at the beginning of the introduction.	We have adapted the introduction to include: “Global demand has nearly doubled over the past decade, reaching an estimated 110 Mt/a by 2025 with market prices ranging between 300 and 600 $/tMeOH. Additionally, projections suggest that demand could exceed 500 Mt/a by 2050.”
	**Reviewer #2:**		
	As communicated to the editor before accepting, I coincidentally reviewed this paper earlier for another journal (only first submission, not the revision). I am pasting my original review below for transparency. I am therefore looking at this new version from a second review round perspective.<br>From what I can see without a rebuttal, I think the authors have done a good work in tackling my earlier comments; however, some points require a bit more attention. More specifically:	First of all, thank you very much for your comments from the first review round, as well as for taking the time to review our submission again. Thank you also for your positive evaluation of our revision efforts. We believe that your remaining concerns can be addressed accordingly (see replies below).	
1	Concerns on the DAC model: the original model used by the authors is now public (ref [21] in the manuscript), but it is not peer reviewed. When quickly scanning through the preprint paper, some concerns remain: the model does not look validated, the DAC cycle is not reported, and Aspen Adsorption can be problematic, for example in closing the mass balance due the finite difference approach.	Thank you. In the meantime, the paper on the DAC model is now peer-reviewed and published in “Energy Conversion and Management” (see updated ref 22 in manuscript). We believe that your main concerns are addressed that way, especially since the published version also contains supplementary information for further clarification. We acknowledge the need for additional research in the field of DAC process models to enable system level assessments as ours.	We have adapted the limitation section to highlight the need for further DAC research: “Our comparison shows that DAC model selection can substantially influence both system efficiency and cost. However, we do not consider one DAC model to be inherently superior to another, as the employed models represent different process designs, sorbent materials, and underlying adsorption isotherm datasets. To enable more reliable cost assessments, advanced DAC models that incorporate state-of-the-art research – i.e. on weather-dependent mass transfer coefficients and validated adsorption behavior – are needed. At present, however, only a limited number of studies provide the necessary performance data across a broad range of ambient conditions. We therefore emphasize the importance of evaluating DAC performance under diverse climatic settings and of publishing detailed process model data

			to enable independent integration into techno-economic assessments.”
2	However, authors have added a new section in the manuscript where they test the DAC-to-MeOH optimization model using a different DAC thermodynamic model (from the group of D. Sholl, now co-author of the submitted manuscript). The water-related results presented in the manuscript look robust to different DAC models, which is indeed good for the findings. However, there are significant differences in terms of energy and costs, which authors duly recognize and discuss. The question that remains open is: which of the two models is more likely to reproduce the physical behavior of DAC? Or, which are the reasons behind this differences (assumptions, modelling choices, sorbent isotherms)? While authors do not enter into a detailed comparison, the outcomes of the DAC-to-MeOH optimization model still depend on how DAC behaves at varying ambient conditions. Therefore, I would like to see authors going a bit more in depth here.	Thank you. We do agree that the substantial differences in terms of energy and cost need further discussion. However, since both models use different sorbents, we do not think that one is superior to the other, but rather that they reflect different materials and operational strategies.	We have included an in-depth discussion on the reasons for the differences observed:<br>“The pronounced differences between the two DAC models can largely be attributed to the substantially higher predicted water uptake and higher resulting heat demand of the alternative model compared to the base DAC model. The reasons for these deviations are manifold and cannot be attributed to a single factor, since both DAC models are based on different sorbents, adsorption formulations, and process assumptions. In particular, the alternative DAC model[29] employs humidity-dependent reaction mechanisms and adsorption kinetics for a proprietary amine-functionalized sorbent[29,32]. In contrast, the base scenario model[22] applies a co-adsorption model[33], where adsorbed water directly influences the equilibrium CO2 uptake of Lewatit[22]. In addition, the operational strategies of both studies differ considerably. Cai et al. evaluate DAC performance using fixed cycle times under all ambient conditions[29], whereas Jajjawi et al. optimize adsorption and desorption durations as a function of temperature and humidity to minimize the specific energy demand. As discussed by Cai et al., fixed cycle times under humid conditions lead to incomplete desorption steps, reducing effective CO2 productivity[29]. This results in higher water-to-CO2 ratios and substantially larger specific heat demands. The base model, in contrast, can partially compensate for unfavorable adsorption conditions through dynamic cycle adaptation, reflected by longer desorption durations under humid conditions[22], leading to a lower co-adsorption ratio and specific heat demand.<br>Overall, the observed differences reflect the sensitivity of DAC performance predictions to the

			underlying sorbent properties, co-adsorption formulations, and operational assumptions. Importantly, despite the considerable deviations in energy demand and LCOM under extreme climatic conditions, the central conclusion of this work remains robust: both DAC models consistently indicate that DAC-based water supply is sufficient for water-conscious methanol production across a wide range of regions and environmental conditions.”
3	On a similar note, authors have not tackled my comment on water recovery: from my understanding, they assume that all water separated is released with CO2. This however is not necessarily true: it depends on the DAC cycle adopted and the kinetics of CO2 and H2O. This information is not available in the SI either. Authors should discuss this point and ideally test what happens when only part of the separated water can be recovered.	Thank you. We would like to clarify that our study does not assume that all adsorbed water is necessarily recovered and released together with CO_2 independent of the DAC process design. Instead, the water utilized in our analysis corresponds specifically to the desorbed water reported in the DAC product stream of the underlying DAC models. Both DAC datasets employed in this study (Jajjawi and Cai) explicitly quantify the H_2O content in the desorbed product stream, which is subsequently used as an input for the downstream electrolysis and methanol synthesis process. We agree that water recovery strongly depends on the applied DAC cycle and kinetics. These effects are reflected in the underlying DAC process models and their reported product stream compositions. To avoid misunderstandings, we have revised the manuscript wording accordingly and want to highlight a core sentence in our methodology on the DAC model: “In the consecutive desorption phase, a combination of a partial vacuum and a temperature gradient serve as the driving force for desorption of the CO2/H2O mixture. The desorbed CO2 and H2O are then separated and can be utilized in downstream process steps.”<br><br>Given the high importance and uncertainty of water supply from DAC, we have additionally performed a	We adapted the wording from “water co-adsorption ratio” to “water supply ratio” or “desorption ratio” at multiple occasions throughout the manuscript to be more precise. We have added an additional clarification in the caption of Fig. 9: “The water ratio refers to the desorbed water in the product stream.”<br><br>We have additionally tested the effect of reduced DAC water supply and added a new subfigure to Fig. 8 to visualize the sensitivity analysis:<br>“Given the study’s focus on evaluating water supply from DAC under varying ambient conditions and the associated uncertainties, Figure 8B shows the effect of reduced or increased water-to-CO2 ratios on the resulting LCOM across multiple regions. In the region with the lowest average water ratio in the base scenario (ARG.17.15_1, 0.48 tH2O/tCO2), reducing the water supply ratio to 75% of the base case increases the LCOM by about 12% due to water constraints and the required system adaptations discussed above. In contrast, the humid region with the highest average water ratio (PER.17.3_1) remains unaffected even when the water ratio is reduced to 40% of the original value. Regions with moderate climates, such as DEU.1.1_1 in central Europe, are also insensitive to substantial reductions in the water ratio. Overall, most regions maintain sufficient water supply even under reduced water ratios, supporting

		sensitivity analysis to test the effect of a reduced water supply from DAC.	the potential of DAC-based water supply despite uncertainties in future developments.”
	**Reviewer #3:**		
	the manuscript offers a compelling paradigm shift: utilizing DAC's water co-adsorption as the sole feedstock for downstream electrolysis and synthesis. The manuscript is well-organized and demonstrates high research value. However, several key aspects regarding dynamic operation, regional economic disparities, and equipment degradation require further justification and clarification before publication.	Thank you for your positive evaluation. We agree that some of our assumptions and modeling choices require further clarification and justification, which we address in the responses below.<br>We would like to emphasize that our study applies a top-down energy system modeling approach aimed at assessing the weather-driven impacts on a global scale, rather than a detailed bottom-up process model. Consequently, some process-level aspects raised in the comments, while valid, lie beyond the scope and resolution of the present framework. Nevertheless, we performed additional model runs where feasible and expanded the discussion to further address these points. We believe that our energy system perspective provides a simplified yet holistic assessment by integrating technological, economic and climate dimensions within a consistent framework.	
1	The study assumes fully flexible operation of the SOEC model to match intermittent renewable energy generation. However, there is a known correlation between the lifespan of SOECs and the frequency of start-up/shut-down cycles. How do the authors account for the impact of SOEC replacement costs, driven by accelerated degradation, on the final LCOM?	Thank you. We agree that intermittent SOEC operation can lead to accelerated degradation due to increased start-up and shut-down cycling. However, the relationship between operating flexibility and degradation is highly complex and nonlinear and is therefore not explicitly represented in our linear optimization framework. Incorporating such nonlinear degradation behavior would substantially increase the computational complexity and render the global assessment conducted in this study infeasible. Since the primary focus of this work is the influence of DAC-derived water supply under varying ambient	We have clarified the limitation in our Discussion: “While our study provides a detailed system-level evaluation with a focus on DAC-enabled production, it does not account for the effects of intermittently switching electrolysis on and off. Frequent cold starts, in particular, could reduce SOEC lifetime due to accelerated degradation[45,46]. The flexible operation of the SOEC is therefore an optimistic assumption, and the resulting LCOM should be interpreted as not including potential additional replacement costs in regions with strongly intermittent operation. Future work should address these effects, for example by

		conditions, a detailed SOEC degradation analysis is beyond the scope of the present study. We have clarified this limitation in the revised manuscript.<br><br>Additionally, we performed a sensitivity analysis to evaluate the effect of increased operational expenditures (opex). While simplified, this approach provides an approximate representation of additional replacement and maintenance costs associated with accelerated degradation while maintaining the linearity of the optimization problem.	modeling the hot standby power requirements of SOEC systems or explicitly incorporating cold-start behavior."<br><br>We have performed a sensitivity analysis to evaluate the effect of increased opex of the SOEC:<br>"In addition to the capex sensitivities shown in Figure 8A, a separate sensitivity analysis was conducted to assess the influence of potentially increased maintenance and replacement requirements by increasing the annual opex of both DAC and SOEC systems from 4% in the base scenario to 6%. For SOEC systems, the increased opex can, for instance, be interpreted as a simplified representation of higher stack replacement costs resulting from intermittent operation and associated degradation. For DAC systems, it may reflect increased maintenance requirements under harsh environmental conditions, such as enhanced filter maintenance due to dust exposure. The results show a comparatively moderate impact on LCOM, with increases of approximately 1-2% for higher DAC opex and 3-4% for higher SOEC opex. While these effects are not negligible, they indicate that even increased maintenance and replacement requirements remain economically manageable within the investigated system configurations."
2	The air-cooling model assumes that operation is not possible at ambient temperatures above 35 °C. However, in arid regions highly suitable for this technology (e.g., the Middle East, Australia), summer daytime temperatures frequently exceed 40 °C. Under such conditions, what is the operational status of the downstream methanol synthesis plant? Does it shut down entirely? Furthermore, how is the thermal inertia of the reactors managed during these extreme temperature periods?	Thank you. Indeed, air cooling is not possible at elevated temperatures. However, the waste heat generated by the synthesis can be handled by a heat pump and either be stored or integrated into the system (see Fig.1). This is exactly what happens in hot regions, where a large share of the generated waste heat is integrated and air cooling is only used in periods with sufficiently low ambient temperature. Therefore, the synthesis does not need to shut down	No changes made.

		under such conditions. See also our paragraph "Air Cooling as a Water-conscious Opportunity":<br>"While these regions experience high average air temperatures (see SI Figure S 7A), which could suggest a need for increased air cooling capacity (see Methods), the opposite is observed. Instead, less waste heat is dissipated through air cooling, and a larger share is integrated into the process via a heat pump (see Figure 7B). This indicates that in regions with elevated temperatures, waste heat is preferentially upgraded and utilized rather than cooled, as the efficiency and cost-effectiveness of the air cooling system declines at higher temperatures (see SI section 1.3)."<br><br>Regarding the thermal inertia, we want to emphasize that the synthesis is not modeled with a detailed process model but rather as a black-box conversion unit, as common in energy system models. Therefore, inertia is not depicted in our model.	
3	The study assumes that water captured via DAC can be directly utilized in the SOEC. Have the authors considered the impact of impurities typically present in such water (e.g., volatile organic compounds, atmospheric dust particles) on subsequent modules and production stages? The necessity of a water purification step would likely affect the overall system's energy efficiency and capital costs.	Thank you. Yes, we have considered this, but the water from DAC is generally believed to be sufficiently pure, at least for SOEC. We have clarified this accordingly.<br><br>For the alternative PEM-based process chain this is, however, a very important aspect. Therefore, we have conducted an additional assessment including water purification.	We have clarified that the water from DAC is believed to be sufficiently pure for utilization in the SOEC in our methods section: "In the base assessment, SOEC technology is selected due to its ability to directly produce syngas and its lower water quality requirements, particularly compared to PEM electrolysis[65]. In addition, previous studies suggest that DAC products can be directly utilized in the SOEC[16] and that water recovered from DAC is of comparable purity to deionized water[66]."<br><br>For the alternative PEM-based process chain, we have conducted an additional sensitivity analysis to quantify the effect of a water purification step: "Additionally, PEM electrolysis requires ultra-pure water[35], necessitating additional purification steps.

			Although the associated energy demand and cost increase are relatively small - approximately 0.2% additional energy demand and 0.3-0.5% additional cost (see SI Figure S16) - the purification process introduces further water losses, which can increase the overall LCOM in arid and water-constrained regions by more than 10% (see SI Figure S 16). Combined with the higher cooling demand of PEM electrolysis, this process chain may therefore be less suitable for water-conscious production in hot and dry climates. Consequently, while PEM-based production appears promising at first glance, these aspects require further detailed investigation to fully evaluate its potential."
4	In arid desert regions where this technology appears most promising, dust storms and poor air quality are common. Would these harsh conditions affect the fan efficiency and filter integrity of the DAC technology? Have the equipment maintenance costs associated with these severe environmental factors been incorporated into the economic model?	Thank you. The harsh conditions would likely affect the DAC plant and lead to increased maintenance costs. To assess this effect, we have conducted an additional sensitivity analysis.	We conducted an additional sensitivity analysis to quantify the influence of increased maintenance cost of the DAC plant, showing that this is relevant but not overly significant: "In addition to the capex sensitivities shown in Figure 8A, a separate sensitivity analysis was conducted to assess the influence of potentially increased maintenance and replacement requirements by increasing the annual opex of both DAC and SOEC systems from 4% in the base scenario to 6%. For SOEC systems, the increased opex can, for instance, be interpreted as a simplified representation of higher stack replacement costs resulting from intermittent operation and associated degradation. For DAC systems, it may reflect increased maintenance requirements under harsh environmental conditions, such as enhanced filter maintenance due to dust exposure. The results show a comparatively moderate impact on LCOM, with increases of approximately 1-2% for higher DAC opex and 3-4% for higher SOEC opex. While these effects are not negligible, they indicate that even increased maintenance and replacement requirements remain

			economically manageable within the investigated system configurations.”
5	The study calculates heat integration based on the total energy balance. However, the main system units operate on different dynamic profiles: the DAC operates on an intermittent/cyclic basis, whereas the SOEC and methanol synthesis ideally operate continuously. Is the proposed heat integration technically feasible without the support of large-capacity thermal energy storage?	Thank you. The optimization model determines both the capacity of the necessary thermal energy storage as well as the hourly operation of the electrolysis and synthesis unit and the waste heat integration. In each time step, energy conservation is ensured, meaning that, if necessary, heat storage is used. The optimization indeed chooses to build relatively large heat storage to balance the operation.	We have clarified the usage of heat storage in our systems: “To balance heat supply and waste heat integration, relatively large heat storage is used (see SI Figure S 18).”
6	It is noted that in the green methanol synthesis unit, the purge gas is combusted to provide heat for upstream processes. Does this step generate significant CO_2 emissions? Has a carbon capture process been considered for this specific stream, or is this carbon loss accounted for in the overall system carbon balance and specific DAC capacity sizing?	Thank you. The carbon loss associated with purge gas combustion is accounted for in the overall system carbon balance and through the optimization-based sizing of the DAC unit. Consequently, the system does not result in net CO_2 emissions, as the required DAC capacity is adjusted accordingly to compensate for these losses.	We have added this explanation to the subsection on the methanol synthesis unit in the methods section: “The associated carbon loss is accounted for through the optimization-based sizing of the DAC unit, ensuring that the overall system does not result in net CO_2 emissions.”
7	The sensitivity analysis indicates that a PEM-based system achieves a lower LCOM across all regions. Given this substantial economic advantage, why was the SOEC chosen as the primary technology over PEM in the main baseline assessment?	Thank you. The SOEC was chosen since previous studies suggest that it can directly utilize the DAC products. Our sensitivity analysis on the alternative PEM-based process is only a first rough assessment without initially accounting for the necessary water purification in that case. We have added this and show that, under dry conditions, this leads to significantly higher costs (see answer to comment #3). Overall, further future work is needed to thoroughly address this.	We have revised our rationale for choosing the SOEC: “In the base assessment, SOEC technology is selected due to its ability to directly produce syngas and its lower water quality requirements, particularly compared to PEM electrolysis[35]. In addition, previous studies suggest that DAC products can be directly utilized in the SOEC[16] and that water recovered from DAC is of comparable purity to deionized water[66].”<br><br>We have conducted an additional assessment where we account for the water purification needed in the case of PEM electrolysis: see answer to your comment #3
8	A fixed discount rate of 8% is applied. However, the Weighted Average Cost of Capital (WACC) varies significantly between regions (e.g., the USA vs. Mauritania). Although mentioned in the discussion, presenting a base case with a uniform WACC obscures	Thank you. We do agree that the WACC varies significantly between regions and heavily influences the cost. We have illustrated that briefly in our sensitivity analysis. However, our study focuses on the influence of ambient/weather conditions and should be seen as a feasibility study or potential assessment	We have adapted the limitations section to further explain the rationale behind the assumption of uniform financing conditions:<br>“Besides technology specific limitations, our study applies uniform techno-economic assumptions across all regions to isolate the effects of weather conditions

	the real-world economic barriers present in developing nations.	for this novel system rather than as a final cost assessment.	and renewable energy availability. Consequently, regional differences in labor costs, financing conditions and potential scale-dependent technology performance are not explicitly represented. In reality, economies of scale and regional economic conditions may affect both system cost and efficiency. Therefore, the presented results should primarily be interpreted as a comparative assessment of the feasibility of DAC-based feedstock provision across different regions.”
9	The calculated LCOM represents the cost strictly at the production site. Arid regions rich in wind and solar resources are often remote and far from deep-water ports or industrial hubs. How would the costs of new infrastructure (pipelines, rail, or transmission grids) impact these economic results?	Thank you. While not inside the scope of our work, transportation is very relevant. A recent study (see ref 40) has investigated the transportation costs of methanol in detail. We have added this to our discussion.	We have added a new paragraph to our discussion: “Finally, although production costs are the primary cost driver, transportation costs must also be considered when matching supply and demand. A recent study shows that maritime shipping is a cost-effective option for methanol transport, adding approximately 23-41 €/tMeOH to methanol costs in 2050[40]. Additional costs for pipeline transport, for example between production sites and ports, are expected to be comparatively low, with reported costs of 2.3-3.5 €/MWh/1000km[40].”
10	In regions with higher relative humidity, excess water production exceeds 2.5 $tH_2O/tMeOH$. Particularly in inland environments, have the environmental or infrastructure costs associated with the discharge, treatment, or storage of this massive volume of excess water been considered?	Thank you. The present study does not explicitly model the infrastructure, treatment, or discharge costs associated with excess water streams. However, the additional sensitivity analysis on water purification indicates that water treatment processes only have a comparatively minor impact on the overall LCOM. This suggests that potential treatment requirements prior to discharge are also unlikely to substantially alter the economic results. Nevertheless, site-specific environmental impacts and water management strategies should be investigated in more detail in future studies.	No changes made.
11	The manuscript mentions that the ERA5 weather data has a spatial resolution of 0.25°×0.25°. Because microclimates in coastal or mountainous arid regions can vary drastically within a ~30 km grid, please	Thank you. The DAC timeseries for energy demand and water supply are generally averages of the individual regions. Quantifying the margin of error for microclimates is very challenging, given that the	We have acknowledged the limitation and impact of potential microclimates:<br>“While the weather data used in this study is globally available at hourly resolution, potential effects of local

	discuss the potential margin of error introduced by this resolution on the DAC performance metrics.	weather data would need to be known. However, we agree that this is an important aspect for exact placement, i.e. in future local siting studies.	microclimates, particularly in mountainous or coastal regions, cannot be captured due to the spatial resolution of the dataset. Such effects may, however, become relevant when determining the exact placement of DAC facilities within individual regions.”
12	The manuscript mentions using buffer storage during times of insufficient ambient air water supply. What is the assumed maximum retention time for this water storage? Furthermore, how does the factor of evaporation loss impact the capacity sizing and economics of this storage?	Thank you. In the present study, no explicit upper limit was imposed on the retention time of the water storage. Instead, the optimization model determines the economically optimal storage capacity and duration endogenously based on the temporal mismatch between water supply and demand. Evaporation losses were not explicitly considered, as closed storage tanks were assumed for the water storage system. Under this assumption, evaporation losses are expected to be negligible and therefore were not included in the assessment.	We have clarified our assumptions in Table S1: “No upper limit is imposed on the retention time, and evaporation losses are not considered, assuming closed storage tanks.”
